# Development of Styryl‐Modified 3,4‐Dihydropyrimidin‐2(1*H*)‐ones as Potential Antitumor Agents

**DOI:** 10.1002/cmdc.202501073

**Published:** 2026-04-07

**Authors:** Konstantinos Panagoulias, Woonghee Kim, Murat Ozdemir, Busra Turan, Adil Mardinoglu, Hasan Turkez, Daniela Trisciuzzi, Orazio Nicolotti, Antonio Di Stefano, Stamatia Vassiliou, Ivana Cacciatore

**Affiliations:** ^1^ Laboratory of Organic Chemistry Department of Chemistry National and Kapodistrian University of Athens Athens Greece; ^2^ Science for Life Laboratory KTH‐Royal Institute of Technology Stockholm Sweden; ^3^ Trustlife Laboratories Drug Research & Development Center Istanbul Turkey; ^4^ Centre for Host‐Microbiome Interactions Faculty of Dentistry, Oral & Craniofacial Sciences King's College London London UK; ^5^ Department of Medical Biology Faculty of Medicine Atatürk University Erzurum Turkey; ^6^ Department of Pharmacy, Pharmaceutical Sciences University of Bari “Aldo Moro” Bari Italy; ^7^ Department of Pharmacy “G. D’Annunzio” University of Chieti‐Pescara Chieti Scalo (Chieti) Italy

**Keywords:** Biginelli reaction, dihydropyrimidine, HeLa cells, MCF‐7 cells, Monastrol

## Abstract

Monastrol, a DHPM‐based Eg5 inhibitor with well‐known antiproliferative activity but limited therapeutic potential due to poor solubility and low bioavailability, was selected as the lead compound for the design of styryl‐modified 3,4‐dihydropyrimidin‐2(1*H*)‐ones with an improved pharmaceutical profile. Twelve derivatives (**10–21**) were synthesized via the Biginelli reaction and evaluated for cytotoxicity in HeLa and MCF‐7 cells. Styryl derivatives **16** and **17** emerged as the most active. In HeLa cells, derivatives **17** (IC50 = 1.3 µM) and **16** (IC50 = 3.7 µM) were approximately 85‐fold and 30‐fold more potent than monastrol (IC50 = 111 µM), respectively. In MCF‐7 cells, derivatives **16** and **17** displayed 18‐ to 20‐fold higher potency than monastrol, respectively. Biological results also indicate that styryl derivatives **16** and **17** induce apoptosis in both HeLa and MCF‐7 cells. In HeLa cells, activation of caspase‐8, ‐9, and ‐3 suggests the involvement of both intrinsic and extrinsic pathways. In contrast, in MCF‐7 cells, the increased expression of p53 and p21, together with PARP cleavage, suggests a p53‐dependent apoptotic response. Derivatives **16** and **17** emerged as promising Eg5 inhibitors from docking studies, but their poor aqueous solubility (0.2–0.7 µM), despite high biological stability, highlights the need for formulation strategies to improve drug‐like properties.

## Graphical abstract

Styryl‐modified 3,4‐dihydropyrimidin‐2(1H)‐ones (DHPMs), designed from the Eg5 inhibitor monastrol, show markedly enhanced antiproliferative activity in HeLa and MCF‐7 cancer cells. Lead compounds **16** and **17** trigger apoptosis through cell‐line‐specific pathways and downregulate c‐Myc, emerging as promising anticancer candidates.

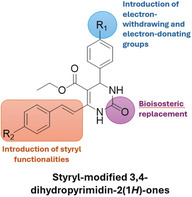

## 1 Introduction

Monastrol was identified for the first time by Mayer et al. in 1999 as an antimitotic agent [1]. It is a small molecule—containing the dihydropyrimidine (DHPM) scaffold—that blocks cells in mitosis by inhibiting Eg5, a member of the kinesin‐5 family, acting as an inhibitor of microtubule polymerization. Inhibition of Eg5 leads to the activation of the spindle assembly checkpoint, causing mitotic cells to arrest in the G2/M phase and consequently undergo cell death [2]. The antiproliferative activity of monastrol against lung, breast, ovarian, glioma, and melanoma cells is well documented in the literature [3, 4, 5].

Although monastrol has shown significant biological activity, its poor solubility, short half‐life, and limited bioavailability have restricted its progress as a therapeutic candidate. To overcome these limitations, several derivatives were synthesized by modifying the substituent on the main dihydropyrimidine (DHPM) scaffold of monastrol [6, 7]. Previous studies indicated that the N3‐phenyl substitution generally enhances cytotoxic activity compared to monastrol. Notably, the presence of electronegative moieties at the meta and para positions enhances the interaction with the active site of Eg5, thereby improving cytotoxic activity. The hydroxyl group on the phenyl ring at C6 enhances the antitumor activity. The replacement of the ethyl ester at C5 with aryl substituents led to improved antiproliferative activities of novel derivatives. Monastrol–fatty acid conjugates were developed by El‐Hamamsy et al. [8], showing potential antitumor activity against C6 rat glioma cells, with IC_50_ values in the range of 5–7 µM, compared to monastrol (IC_50_ = 20 µM). The substitution at C4 was investigated by Wazalwar et al., who discovered novel inhibitors of the epidermal growth factor receptor tyrosine kinase [9].

**FIGURE 1:**
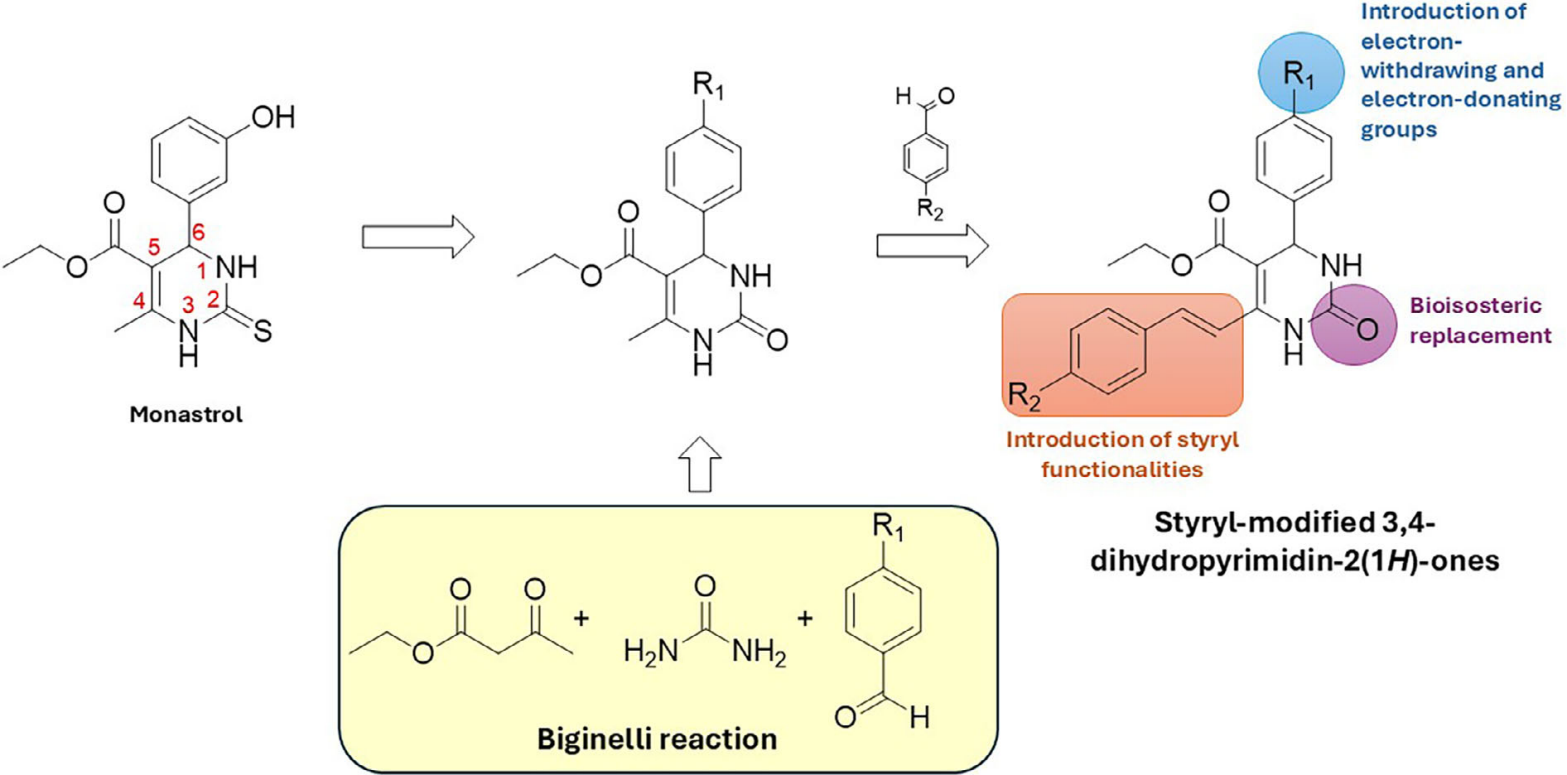
Designed and synthesized styryl‐modified 3,4‐dihydropyrimidin‐2(1*H*)‐ones via the Biginelli reaction.

Based on these findings, the aim of this work was to design and synthesize styryl‐modified 3,4‐dihydropyrimidin‐2(1*H*)‐ones with improved metabolic stability and antiproliferative activity—through structural modifications at the C2, C4, and C6 positions of the DHPM scaffold—compare to monastrol. Initially, a bioisosteric replacement of the sulfur atom at C2 in monastrol was carried out by introducing an oxygen atom. The bioisosteric replacement of sulfur with oxygen increases the molecule's polarity and chemical stability, while reducing its lipophilicity. The main modifications were performed at position C‐4 and C‐6 of the DHPM scaffold by the introduction of styryl moieties and a substituted phenyl ring with electron‐withdrawing and electron‐donating groups, respectively (Figure 1).

For the synthesis of styryl derivatives **10–21**, the Biginelli reaction was used. It is a multicomponent reaction that involves the acid‐catalyzed cyclocondensation between a β‐ketoester, an aldehyde and (thio)urea to give 3,4‐dihydropyrimidine‐2(1*H*)‐(thi)ones DHPMs (Figure 1) [10]. It is a simple, selective and atom‐economic reaction leading to structurally variable derivatives by modifying one or more of the three components. Therefore, it has become a useful tool for organic chemists to synthesize derivatives with interesting biological properties. Thus, 12 styryl derivatives (**10–21**) were synthesized, and cell viability assays were performed on HeLa and MCF‐7 cell lines to evaluate their cytotoxicity. The most active derivatives (**16** and **17**) were further investigated to elucidate their mechanisms of action in HeLa and MCF‐7 cells. The physicochemical properties of compounds **16** and **17** (such as solubility and lipophilicity), as well as their stability in plasma and in simulated gastric and intestinal fluids, were also examined.

## 2 Results and Discussion

### 2.1 Chemistry

**SCHEME 1:**
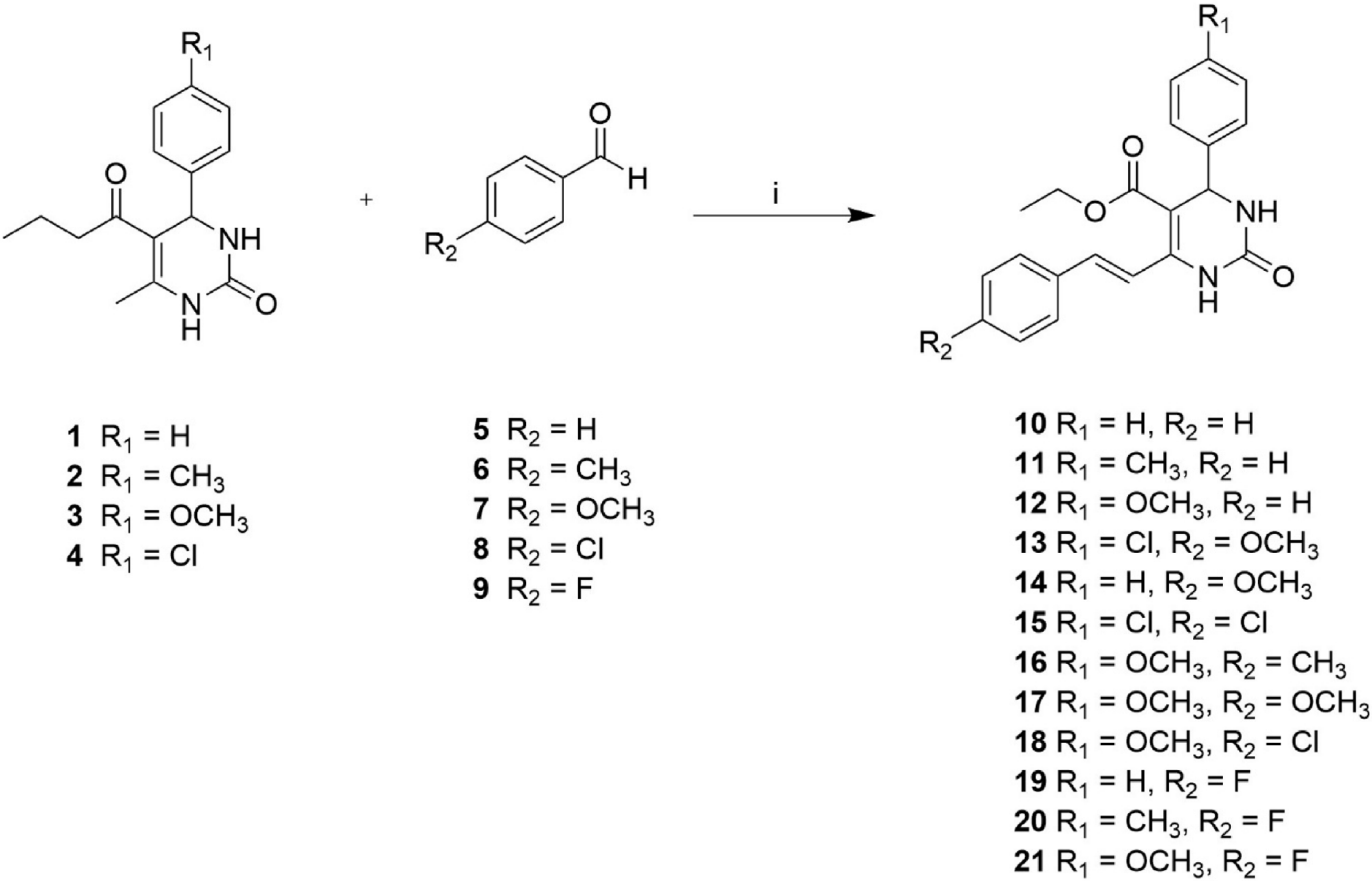
Reagents and conditions: (i) *p*‐toluene sulfonic acid (PTSA), xylene, 110°C, 2–5 days.

As reported in literature, 3,4‐dihydropyrimidin‐2(1*H*)‐ones (DHPMs) **1–4** were obtained using the Biginelli cyclocondensation reaction [11, 12]. A reaction between suitable aldehydes, ethyl acetoacetate, and urea in the presence of a catalytic amount of iron (III) chloride hexahydrate and a few drops of 36% w/v HCl provided oxygen‐containing derivatives **1–4** in good yields. Olefination of the methyl group was achieved by a vinylogous aldol condensation reaction of dihydropyrimidin‐2(1*H*)‐ones **1–4** with aldehydes **5–9** and catalytic *p*‐toluene sulfonic acid (PTSA) in boiling xylene to give styryl 3,4‐dihydropyrimidin‐2(1*H*)‐ones **10–21** in medium yields and exclusively as *E*‐isomers (Scheme 1) [13]. All compounds were characterized by NMR and MS spectroscopy.

### 2.2 Biological Studies

### 2.3 Cell Viability Assays on HeLa and MCF‐7 Cell Lines

HeLa (cervical adenocarcinoma) and MCF‐7 (human breast cancer) cell lines were chosen as in vitro experimental models since they overexpress the Eg5 enzyme, the main target of monastrol [14]. According to literature data, monastrol shows IC50 values of 88 and 111 µM in HeLa and MCF‐7 cell lines, respectively [15]. Thus, all styryl derivatives **10–21** were evaluated for their cytotoxicity against HeLa and MCF‐7 cell lines using the MTT assay.

**FIGURE 2:**
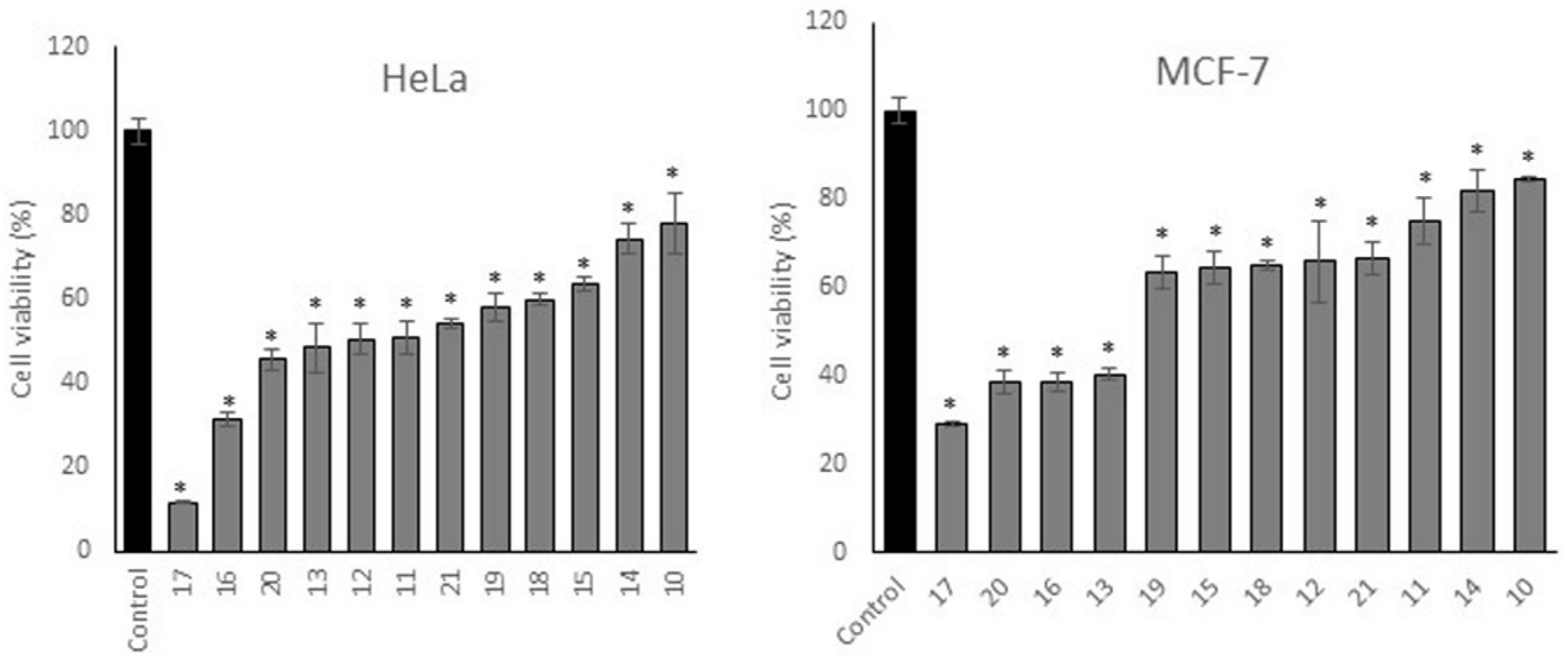
Screening of styryl derivatives **10–21** for anticancer activity in HeLa and MCF‐7 cell lines. HeLa (cervical cancer) and MCF‐7 (breast cancer) cells (10 000 cells/well) were treated with styryl derivatives **10–21**. Cell viability was assessed using the MTT assay after 48 h of exposure at 10 µM. Data are presented as mean ± SD. *p < 0.05.

Cells (10,000/well) were treated with styryl derivatives **10–21** at a concentration of 10 µM for 48 h (Figure 2). Styryl derivatives **10–21** showed a good inhibitory effect on both cell lines. Among them, derivative **17** demonstrated the most potent activity, reducing cell viability to 11.6% in HeLa cells and 29.1% in MCF‐7 cells. The second most active compound against HeLa was derivative **16**, which decreased cell viability to 31.2%. On MCF‐7 cells, derivatives **20** (38.5%) and **16** (38.6%) also showed strong cytotoxic effects.

### 2.4 Comparative Cytotoxicity Evaluation of 16 and 17 With Docetaxel

**FIGURE 3:**
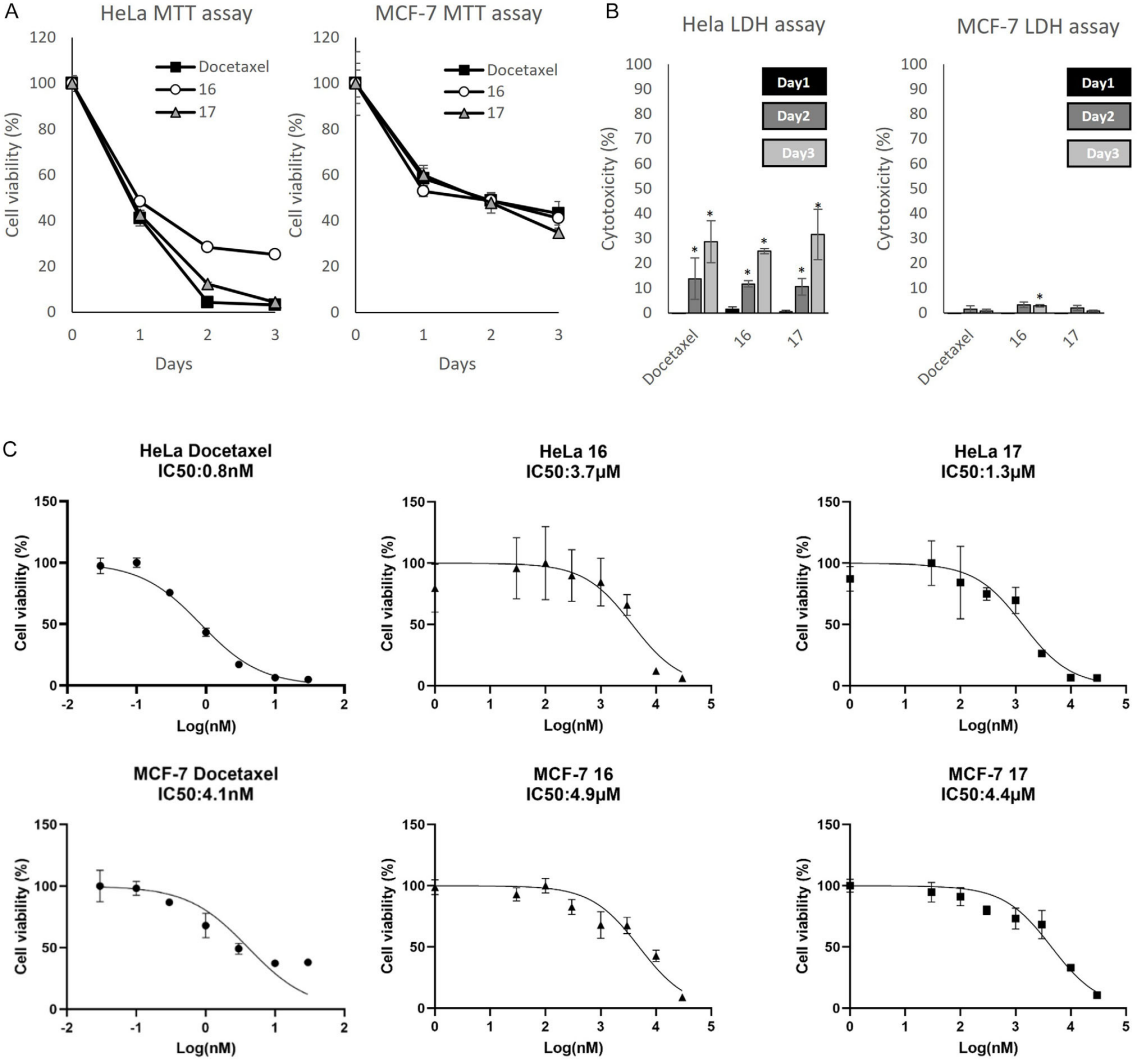
Comparative cytotoxicity evaluation of docetaxel and styryl derivatives **16–17**, including time‐dependent cell viability and cytotoxicity assays, as well as dose‐dependent IC_50_ determination in HeLa and MCF‐7 cell lines. (A) Time‐dependent cell viability and (B) cytotoxicity were measured over 3 days following treatment with 10 µM compounds (10 000 cells/well). (C) Dose‐dependent IC_50_ values were determined after 3 days of treatment. Data are presented as mean ± SD. *p < 0.05.

Styryl derivatives **16–17** were identified as hit compounds during the initial biological screening. To further investigate their antitumor effect, we performed time‐ and dose‐dependent studies (Figure 3). Cell viability and cytotoxicity (LDH assay) were monitored for 3 days following treatment of 10 000 cells with 10 µM compounds (Figure 3A). Docetaxel, a microtubule‐targeting agent in cancer chemotherapy, was employed as a reference compound for biological assays. On HeLa cells, styryl derivative **17** reduced viability to 42.6%, 12.4%, and 4.3% on days 1, 2, and 3, respectively, whereas docetaxel decreased viability to 41.1%, 4.3%, and 3.2% over the same period. On MCF‐7 cells, styryl derivative **17** showed a stronger inhibitory effect on cell viability (34.7%) compared to docetaxel (43.2%) on day 3. Time‐dependent cytotoxicity assays revealed that ddocetaxel and styryl derivatives **16–17** increased cytotoxicity. However, cytotoxic effects were less pronounced than the corresponding inhibition of cell viability. For instance, styryl derivative **17** reduced cell viability to 4.3% on day 3 in HeLa cells, yet cytotoxicity reached only 31.5%. Similarly, docetaxel lowered viability to 3.2% on day 3 but induced only 28.6% cytotoxicity. On MCF‐7 cells, compounds showed minimal cytotoxicity: Docetaxel 0.8%, derivative **16** 2.8%, and derivative **17** 0.8% at day 3. The IC_50_ values from dose–response viability assays were 0.8 nM (HeLa) and 4.1 nM (MCF‐7) for docetaxel, 3.7 µM (HeLa) and 4.9 µM (MCF‐7) for derivative **16**, and 1.3 µM (HeLa) and 4.4 µM (MCF‐7) for derivative **17**.

These findings suggest that the antitumor effects of derivatives **16–17** may resemble those of docetaxel, exerting strong inhibition of cell viability not primarily through necrosis, but rather via induction of apoptosis and/or cell cycle arrest. Based on IC_50_ values obtained in HeLa cells, styryl derivatives **17** (1.3 µM) and **16** (3.7 µM) are approximately 85‐fold and 30‐fold more potent than monastrol (111 µM), respectively [14]. On the other hand, both derivatives **16** and **17** showed lower IC_50_ values compared to monastrol (IC_50_ = 88 µM) in MCF‐7 cells, being approximately 18‐fold and 20‐fold more potent, respectively.

### 2.5 Styryl Derivatives 16–17 Induce Apoptotic Cell Death Through Different Mechanisms

**FIGURE 4:**
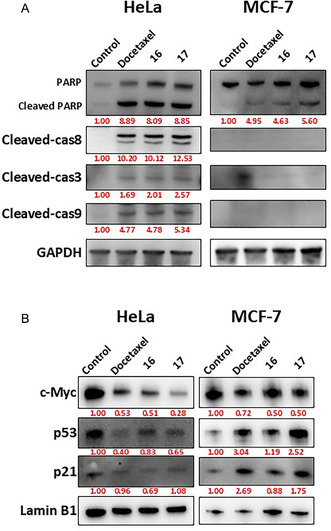
Western blot analysis of docetaxel and styryl derivatives **16–17** in HeLa and MCF‐7 cell lines. (A) Whole‐cell lysates were analyzed for apoptosis‐related proteins after 2 days of treatment with 10 µM compounds. (B) Nuclear extracts were examined for oncogenic and tumor suppressor proteins, including p53, c‐Myc, and p21. Band intensities were quantified using the ImageJ software.

The ability of the compounds to induce apoptosis was evaluated by western blot analysis (Figure 4). Cleaved PARP, a marker of programed cell death, was strongly increased in HeLa whole cell lysates by docetaxel (8.89‐fold), derivative **16** (8.09‐fold), and derivative **17** (8.85‐fold), and in MCF‐7 lysates by docetaxel (4.95‐fold), styryl derivative **16** (4.63‐fold), and derivative **17** (5.60‐fold). In HeLa cells, cleaved caspase‐8, caspase‐9, and caspase‐3, key proteins of the extrinsic and intrinsic apoptosis pathways, were highly expressed, indicating activation of both apoptosis cascades (Figure 4A). Nuclear protein levels of c‐Myc, p53, and p21 were also assessed (Figure 4B). The oncogenic transcription factor c‐Myc was significantly downregulated in both HeLa and MCF‐7 cells. In contrast, p53 and p21, crucial regulators of apoptosis and cell cycle arrest, showed cell type–specific responses: (a) in HeLa cells, docetaxel (0.4 and 0.96‐fold), derivative **16** (0.51 and 0.69‐fold), and derivative **17** (0.65 and 1.08‐fold) decreased or did not significantly alter p53 and p21 levels and( b) in MCF‐7 cells, docetaxel (3.04 and 2.52‐fold), derivative **16** (1.19 and 0.88‐fold), and derivative **17** (2.52 and 1.75‐fold) increased p53 and p21 expression. Notably, derivative **17** affected nuclear p53 and p21 differently from derivative **16** but showed effects like docetaxel.

These results indicate that styryl derivatives **16** and **17** induce apoptosis in both HeLa and MCF‐7 cells, though through different molecular mechanisms. In HeLa cells, activation of caspase‐8, ‐9, and ‐3 suggests the involvement of both intrinsic and extrinsic pathways, occurring through a p53‐independent mechanism. In contrast, in MCF‐7 cells, the increased expression of p53 and p21, together with PARP cleavage, suggests a p53‐dependent apoptotic response. The consistent downregulation of c‐Myc in both cell lines further supports the pro‐apoptotic role of these derivatives. Notably, derivative **17** exhibited an apoptotic profile similar to docetaxel and more pronounced than monastrol, highlighting it as the most promising candidate for further mechanistic and pharmacological investigations.

Considering biological results, a preliminary structure–activity relationship (SAR) analysis of all styryl derivatives **10–21** suggests that the presence of a methoxy group on the aryl ring at C6 enhances cytotoxicity in HeLa and MCF‐7 cells, as evidenced by the high potency of derivatives **16** and **17**. Moreover, the introduction of an additional methoxy substituent in the para position of the styryl moiety at C4 further strengthens the antitumor potential of derivative **17** compared to derivative **16**, which bears a methyl group at the same position. In fact, based on IC_50_ values obtained in HeLa cells, styryl derivative **17** (1.3 µM) was in 55‐fold more potent than **16** (3.7 µM). This simple structural difference between **16** and **17**—differing only by the para substituent on the aromatic ring (methyl in **16** and methoxy in **17**)—suggests that the presence of an electron‐donating and weakly hydrogen‐bond–accepting methoxy group enhances biological activity. This conclusion is further supported by the observation that styryl derivative **10**, which lacks substituents on both aryl rings, is devoid of cytotoxicity in either cell line. The introduction of halogen atoms in styryl derivatives **15**, **18**, **19**, and **21** did not result in significant cytotoxicity in the experimental models used for the biological assays.

### 2.6 Physicochemical Characterization and Stability Studies

Styryl derivatives **16–17**, which displayed the most relevant biological activities, were evaluated for their physicochemical properties—water solubility and lipophilicity—and stability in standard fluids such as SGF, SIF, and plasma [16, 17]. According to the USP classification, both compounds under investigation, with solubility values of 0.2 and 0.7 µM, can be classified as practically insoluble substances (Table 1). Although compound **17** exhibits a slightly higher solubility (approximately 3.5‐fold greater than compound **16**), its oral bioavailability would still be severely limited. Styryl derivative **17** possesses a value of water solubility similar to monastrol (0.67 µM, value calculated using Advanced Chemistry Development (ACD/Labs) Software (1994–2025 ACD/Labs). In terms of lipophilicity, styryl derivative **17** has a Log D of 4.04, while derivative **16** exceeds 4.5, indicating high lipophilicity (Table 2). This may enhance cell membrane permeation and antitumor potential, but also suggests poor solubility, as evidenced by solubility values reported in Table 1. Stability studies showed that both styryl derivatives exhibit half‐lives (*t*_1__/__2_) greater than 350 min in simulated gastric fluid (SGF), simulated intestinal fluid (SIF), and plasma, indicating considerable chemical stability under physiological‐like conditions (Table 3). Both compounds have a molecular weight around 400 Da, which is favorable for intestinal absorption according to Lipinski's rules. Taken together, these findings suggest that while styryl derivatives **16–17** are chemically stable and lipophilic enough to permeate cellular membranes, their extremely low solubility represents a major limitation that would need to be addressed through formulation strategies or structural optimization to improve pharmacokinetic performance and therapeutic applicability.

**TABLE 1:** Kinetic solubility data of styryl derivatives **16–17** in PBS pH 7.4.

Compound	Solubility, μM
Progesterone (positive control)	22.98
Diclofenac (positive control)	229.52
**16**	0.27
**17**	0.73

**TABLE 2:** LogD data of styryl derivatives **16–17** in *n*‐Octanol /PBS pH 7.4.

Compound	Log *D*
Nicardipine (positive control)	4,09
**16**	>4.5
**17**	4.04

**TABLE 3:** Stability of styryl derivatives **16–17** in plasma, SGF, and SIF.

Compound	Buffer	Remaining percentages, %		*t*_1/2_, min
		0 min	120 min	
Positive control (propantheline)	Human plasma (pH 8.28)	100	0.04	10.63
**16**		100	109.12	>372.67
**17**		100	98.83	>372.67
Positive control (erythromycin)	SGF (pH 1.48)	100	5.32	28.35
**16**		100	97.12	>372.67
**17**		100	101.87	>372.67
Positive control (chlorambucil)	SIF (pH 6.87)	100	1.79	20.67
**16**		100	113.99	>372.67
**17**		100	106.96	>372.67

### 2.7 Molecular Docking Studies

**FIGURE 5:**
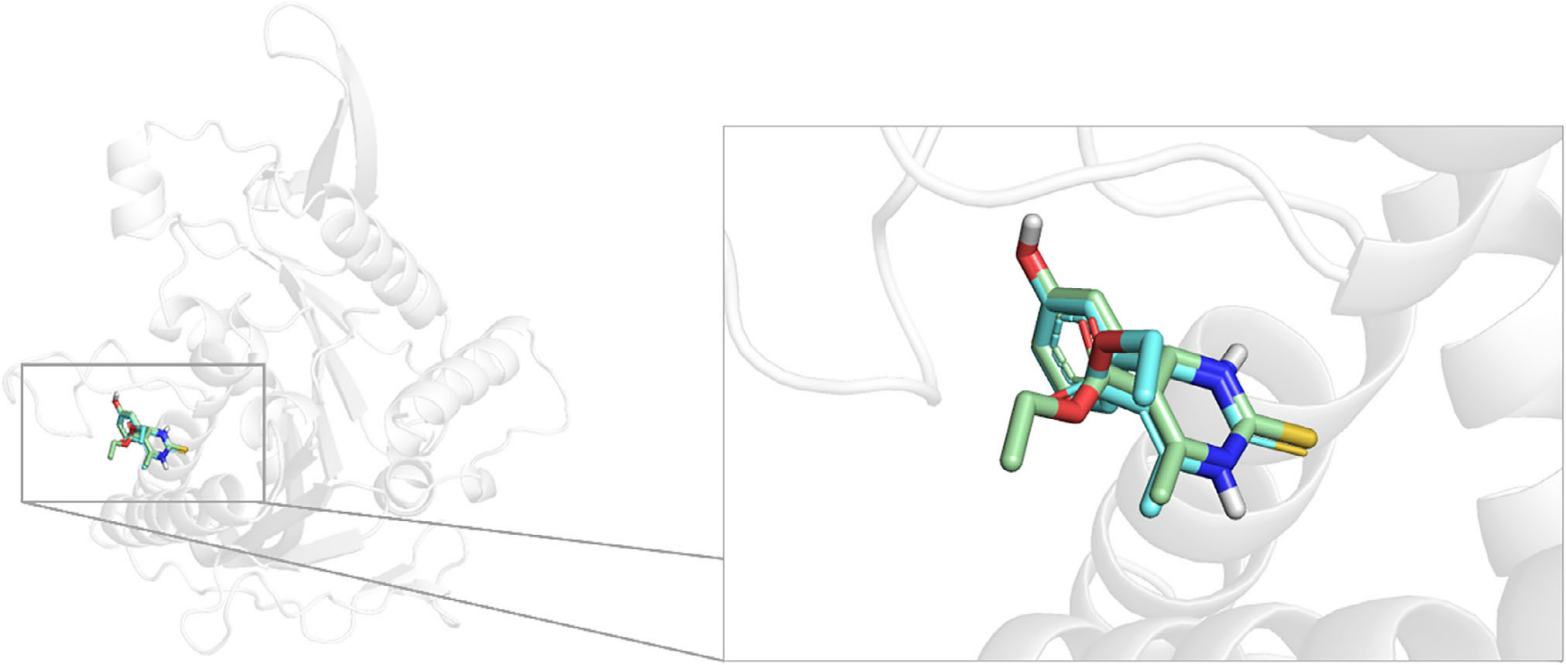
Zoomed‐in view at Eg5 binding site of X‐ray (PDB 1Q0B) solved and top‐scored docking pose of the cognate ligand monastrol depicted in green and cyan sticks, respectively. For the sake of clarity, only the polar hydrogen atoms are reported, whereas the Eg5 is shown as gray cartoon.

Molecular docking simulations were carried out on **16** and **17** toward Eg5 by employing MZDock software [18]. As reported in the Methods, the docking protocol was first calibrated by docking back the cognate ligand monastrol (PDB entry 1Q0B [19]) into its native binding site. Notably, the cognate ligand successfully reproduced its experimental orientation, returning a root‐mean‐square deviation (RMSD) as low as 1.225 Å accounted over all heavy atoms, thereby confirming the reliability of the procedure. The superimposition of the X‐ray solved and the top‐scored docking poses of the cognate ligand monastrol is shown in Figure 5.

**FIGURE 6:**
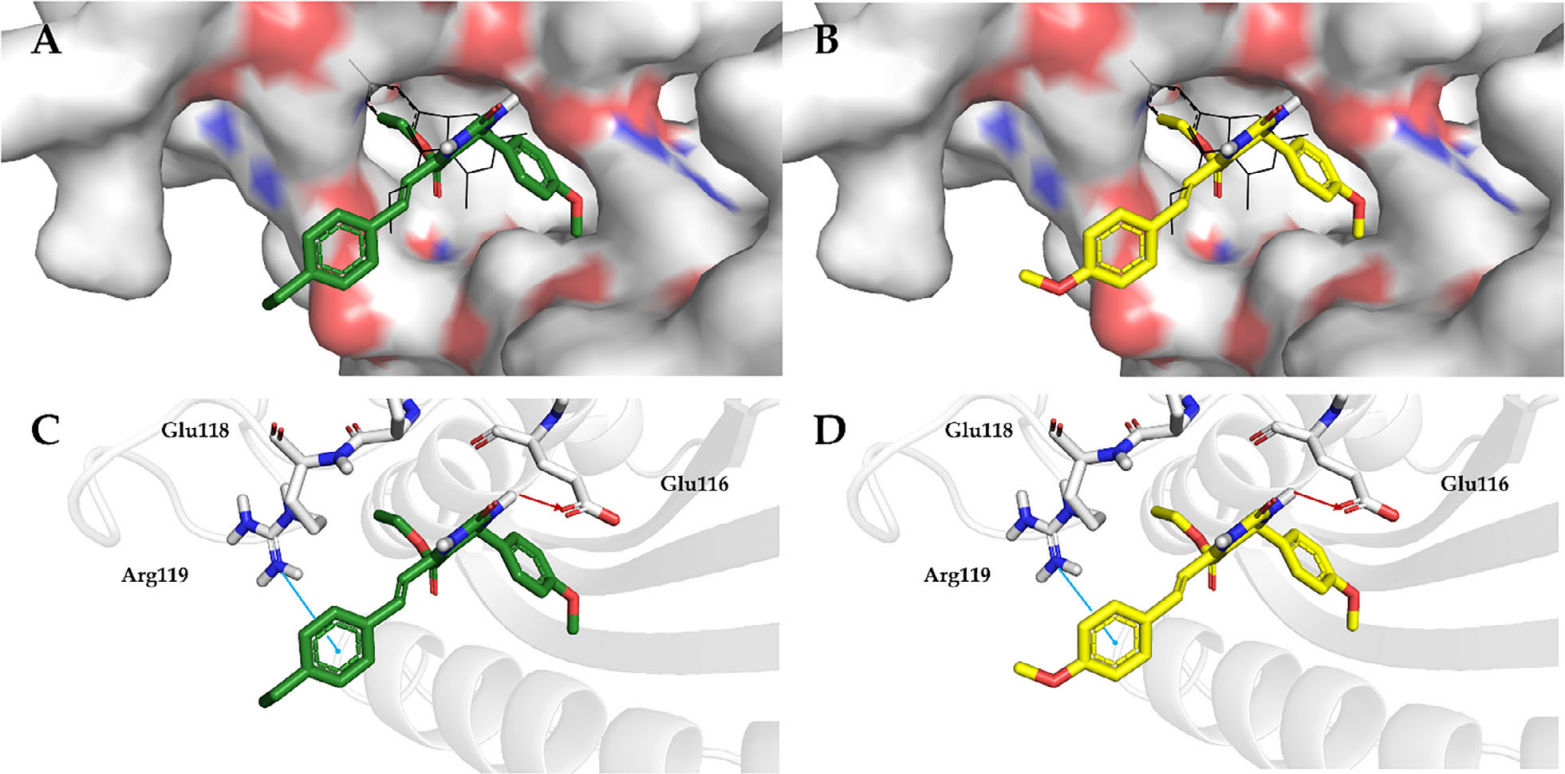
(A, B) Top‐scored docking poses within the Eg5 binding site of **16** and **17.** (C, D) Key interactions with the most relevant residues. Hydrogen bonds and cation‐π interactions are represented in red and cyan lines. Ligands are rendered as sticks, whereas the protein is represented as a cartoon. Specifically, the monastrol is depicted in black lines. Residues located within 5 Å are highlighted as a colored surface. For clarity, only polar hydrogen atoms are displayed.

The key interactions of the top‐scored docking poses of **16** and **17** with the relevant residues of the Eg5 binding site are depicted in Figure 6, providing structural insights into the predicted binding mode and supporting its potential as effective binders.

As shown in Figure 6, the docking poses of both compounds were well accommodated within the Eg5 binding pocket and showed good overlap with the cognate ligand monastrol. This is consistent with the favorable docking scores obtained for **16** and **17** (−7.7 and −7.6 kcal/mol, respectively), which are comparable to the docking score of monastrol (−8.1 kcal/mol), suggesting that both derivatives can adopt stable binding orientations within the site. Notably, we observed that top‐scored poses of **16** and **17** maintained a similar hydrogen bond interaction formed by the nitrogen atom at position 1 of monastrol and Glu116 of Eg5. Moreover, both compounds formed cation–π interactions with the backbone of Arg119 at a distance of 5.6 Å. These interactions are critical for engagement within this region and support the potential of these derivatives as Eg5 binders. The enhanced antiproliferative activity of derivatives **16** and **17** may be attributed to the nature and substitution pattern of the methoxy and hydroxy groups on the aromatic rings. Compared with monastrol, which bears a single hydroxy group on the aromatic ring at position P6, the introduction of methoxy substituents in derivatives **16** and **17** increases aromatic electron density and lipophilicity, thereby favoring stronger interactions within the Eg5 binding pocket. Moreover, replacement of the ethyl ester moiety at P4 in native monastrol with a styryl group further enhances hydrophobic interactions with the binding site. These observations are consistent with docking results, which identified derivatives **16** and **17** as the most promising Eg5 inhibitors within the series.

## 3 Conclusion

In conclusion, the results of this study demonstrate that structural modification of the DHPM scaffold through the introduction of styryl moieties significantly enhances the antiproliferative activity of monastrol derivatives. Styryl derivatives **16** and **17** emerged as the most potent, showing markedly higher cytotoxicity than monastrol in both HeLa and MCF‐7 cells. Biological studies revealed that these derivatives induce apoptosis through distinct, cell line‐specific pathways: HeLa cells undergo p53‐independent apoptosis via activation of both intrinsic and extrinsic caspase pathways, while MCF‐7 cells display a p53‐dependent response accompanied by PARP cleavage and upregulation of p21. Notably, derivative **17** exhibited an apoptotic profile comparable to that of docetaxel and superior to monastrol, highlighting its promise as a lead candidate for further pharmacological investigations. Taken together, the docking results highlight compounds **16** and **17** as promising Eg5 inhibitors, owing to their favorable binding energies and conservation of critical interactions observed for monastrol. Although derivatives **16** and **17** show excellent stability in biological fluids, their limited aqueous solubility may restrict bioavailability, suggesting that future work should focus on formulation strategies or delivery systems to optimize their drug‐like properties. Overall, styryl‐modified DHPMs represent a promising class of antitumor agents with enhanced potency and well‐characterized mechanisms of action.

## 4 Experimental

Reagents were purchased from Aldrich and TCI Chemicals and were of analytical grade. Reactions were monitored by thin‐layer chromatography (TLC) carried out on silica gel plates (Macherey Nagel SIL G UV_254_) and visualized under UV light. Purification of the compounds was performed by filtration or by column chromatography. Column chromatography was carried out on silica gel (Merck, 70−230 mesh) using appropriate solvents. PE = petroleum ether (40°C–60°C). DCM = dichloromethane, EA = ethyl acetate. ^1^H, ^19^F, ^13^C, and 2D‐NMR spectra were recorded on a Bruker 400 MHz Avance spectrometer (Ettlingen, Germany). ^13^C NMR spectra are fully proton decoupled. Electrospray ionization (ESI) mass spectra were obtained on an MSQ Surveyor mass spectrometer (Thermo Scientific, Bremen, Germany) using direct sample injection in the positive or negative mode. High‐resolution mass spectrometry (HRMS) spectra were acquired on a 4800 MALDI‐TOF mass spectrometer (Applied Biosystems, Foster City, US) in positive reflection mode over an m/z range of 100−700. All synthesized compounds gave satisfactory NMR spectra and HRMS data.

### 4.1 General Method for the Synthesis of 10–21

To a suspension of derivatives **1–4** (0.5 mmol) in xylene (10 mL), aromatic aldehyde **5–9** (1 mmol) and catalytic pTSA were added, and the reaction mixture was refluxed at 110°C for 2–5 days. The progress of the reaction was monitored by TLC. Upon completion, the reaction mixture was concentrated in vacuo, extracted with DCM/H_2_O, and the organic phase was dried over Na_2_SO_4_. The solvent was removed under reduced pressure, and the crude product was purified by column chromatography using PE/AcOEt as the eluent.

#### 4.1.1 Ethyl (E)‐2‐oxo‐4‐phenyl‐6‐styryl‐1,2,3,4‐tetrahydropyrimidine‐5‐carboxylate (10)

Following the general procedure, derivative **1** (156 mg, 0.6 mmol) and benzaldehyde **5** (122 μL, 1.2 mmol) were reacted for 5 days to afford styryl derivative **10** as white solid (134 mg, 48% yield). Rf = 0.52 (PE/EA 4:6). ^1^H NMR (400 MHz, DMSO‐*d*_6_) *δ* 9.22 (s, 1H), 7.93 (d, *J* = 16.7 Hz, 1H), 7.86 (s, 1H), 7.54–7.23 (m, 11H), 5.26 (d, *J* = 2.9 Hz, 1H), 4.07 (q, *J* = 7.1 Hz, 2H), 1.15 (t, *J* = 7.1 Hz, 3H). ^13^C NMR (101 MHz, DMSO‐*d*_6_) *δ* 165.20, 152.51, 144.63, 144.34, 135.99, 134.81, 129.11, 129.00, 128.53, 127.47, 127.15, 126.28, 119.60, 102.01, 59.83, 53.98, 14.06. Spectroscopic data are in accordance with the literature [13]. MS (ESI) calculated for (C_21_H_21_N_2_O_3_)^+^ (M + H)^+^ m/z = 349.15, found m/z = 349.18. HRMS m/z calculated for (C_21_H_21_N_2_O_3_)^+^ (M + H)^+^ 349.1547, found 349.1548.

#### 4.1.2 Ethyl (E)‐2‐oxo‐6‐styryl‐4‐(p‐tolyl)‐1,2,3,4‐tetrahydropyrimidine‐5‐carboxylate (11)

Following the general procedure, derivative **2** (220 mg, 0.8 mmol) and benzaldehyde **5** (163 μL, 1.6 mmol) were reacted for 2 days to afford styryl derivative **11** as a white solid (101 mg, 35% yield). Rf = 0.73 (PE/EA, 4:6). ^1^H NMR (400 MHz, DMSO‐*d*_6_) *δ* 9.18 (s, 1H), 7.93 (d, *J* = 16.6 Hz, 1H), 7.81 (s, 1H), 7.56–7.28 (m, 6H), 7.19–7.12 (m, 4H), 5.22 (d, *J* = 3.4 Hz, 1H), 4.06 (q, *J* = 7.1, 2H), 2.27 (s, 3H), 1.16 (t, *J* = 7.1 Hz, 3H). ^13^C NMR (101 MHz, DMSO‐*d*_6_) *δ* 165.23, 152.55, 144.46, 141.41, 136.64, 136.02, 134.71, 129.10, 129.04, 129.01, 127.15, 126.20, 119.62, 102.20, 59.82, 53.68, 20.68, 14.09. MS (ESI) calculated for (C_22_H_23_N_2_O_3_)^+^ (M + H)^+^ m/z = 363.16 found m/z = 363.21. HRMS m/z calculated for (C_22_H_23_N_2_O_3_)^+^ (M + H)^+^ 363,1703, found 363,1704.

#### 4.1.3 Ethyl (E)‐4‐(4‐methoxyphenyl)‐2‐oxo‐6‐styryl‐1,2,3,4‐tetrahydropyrimidine‐5‐carboxylate (12)

Following the general procedure, derivative **3** (174 mg, 0.6 mmol) and benzaldehyde **5** (122 μL, 1.2 mmol) were reacted for 2 days to afford styryl derivative **12** as white solid (99 mg, 44% yield). Rf = 0.50 (PE/EA 4:6). ^1^H NMR (400 MHz, DMSO‐*d*_6_) *δ* 9.19 (s, 1H), 7.93 (d, *J* = 16.0 Hz, 1H), 7.80 (s, 1H), 7.55–7.27 (m, 6H), 7.20 (d, *J* = 8.3 Hz, 2H), 6.90 (d, *J* = 8.1 Hz, 2H), 5.20 (s, 1H), 4.06 (q, *J* = 7.0 Hz, 2H), 3.72 (s, 3H), 1.16 (t, *J* = 7.0 Hz, 3H). ^13^C NMR (101 MHz, DMSO‐*d*_6_) *δ* 165.23, 158.59, 152.51, 144.32, 136.48, 136.03, 134.66, 129.08, 129.01, 127.46, 127.13, 119.63, 113.85, 102.35, 59.80, 55.09, 53.38, 14.10. MS (ESI) calculated for (C_22_H_23_N_2_O_4_)^+^ (M + H)^+^ m/z = 379.16, found m/z = 379.26. HRMS m/z calculated for (C_22_H_23_N_2_O_4_)^+^ (M + H)^+^ 379.1652, found 379.1654.

#### 4.1.4 Ethyl (E)‐4‐(4‐chlorophenyl)‐6‐(4‐methoxystyryl)‐2‐oxo‐1,2,3,4‐tetrahydropyrimidine‐5‐carboxylate (13)

Following the general procedure, derivative **4** (236 mg, 0.8 mmol) and anisaldehyde **7** (195 μL, 1.6 mmol) were reacted for 2 days to afford styryl derivative **13** as a white solid (101 mg, 31% yield). Rf = 0.48 (PE/EA 4:6). ^1^H NMR (400 MHz, DMSO‐*d*_6_) *δ* 9.21 (s, 1H), 7.88 (s, 1H), 7.81 (d, *J* = 16.6 Hz, 1H), 7.50–7.39 (m, 5H), 7.29 (d, *J* = 8.2 Hz, 2H), 6.99 (d, *J* = 8.3 Hz, 2H), 5.24 (d, *J* = 3.5 Hz, 1H), 4.06 (q, *J* = 7.0 Hz, 2H), 3.79 (s, 3H), 1.15 (t, *J* = 7.1 Hz, 3H). ^13^C NMR (101 MHz, DMSO‐*d*_6_) *δ* 165.15, 160.18, 152.37, 145.36, 143.38, 134.83, 131.93, 128.73, 128.52, 128.50, 128.20, 117.02, 114.52, 100.64, 59.79, 55.28, 53.36, 14.09. MS (ESI) calculated for (C_22_H_22_ClN_2_O_4_)^+^ (M + H)^+^ m/z = 413.12, found m/z = 413.22. HRMS m/z calculated for (C_22_H_22_ClN_2_O_4_)^+^ (M + H)^+^ 413.1263, found 413.1265.

#### 4.1.5 Ethyl (E)‐6‐(4‐methoxystyryl)‐2‐oxo‐4‐phenyl‐1,2,3,4‐tetrahydropyrimidine‐5‐carboxylate (14)

Following the general procedure, derivative **1** (156 mg, 0.6 mmol) and anisaldehyde **7** (146 μL, 1.2 mmol) were reacted for 3 days to afford styryl derivative **14** as a white solid (90 mg, 40% yield). Rf = 0.44 (PE/EA 4:6). ^1^H NMR (400 MHz, DMSO‐*d*_6_) *δ* 9.15 (s, 1H), 7.82 (d, *J* = 16.7 Hz, 2H), 7.49–7.23 (m, 8H), 6.99 (d, *J* = 8.3 Hz, 2H), 5.25 (d, *J* = 3.4 Hz, 1H), 4.06 (q, *J* = 7.0 Hz, 2H), 3.79 (s, 3H), 1.15 (t, *J* = 7.1 Hz, 3H). ^13^C NMR (101 MHz, DMSO‐*d*_6_) *δ* 165.28, 160.14, 152.56, 145.05, 144.45, 134.59, 128.70, 128.56, 128.50, 127.41, 126.26, 117.14, 114.51, 101.18, 59.73, 55.27, 53.92, 14.09. MS (ESI) calculated for (C_22_H_23_N_2_O_4_)^+^ (M + H)^+^ m/z = 379.16 found m/z = 379.16. HRMS m/z calculated for (C_22_H_23_N_2_O_4_)^+^ (M + H)^+^ 379.1652, found 379.1652.

#### 4.1.6 Ethyl (E)‐4‐(4‐chlorophenyl)‐6‐(4‐chlorostyryl)‐2‐oxo‐1,2,3,4‐tetrahydropyrimidine‐5‐carboxylate (15)

Following the general procedure, derivative **4** (236 mg, 0.8 mmol), and 4‐chloro benzaldehyde **8** (225 mg, 1.6 mmol) were reacted for 5 days to afford styryl derivative **15** as a white solid (110 mg, 33% yield). Rf = 0.66 (PE/EA 4:6). ^1^H NMR (400 MHz, DMSO‐*d*_6_) *δ* 9.27 (s, 1H), 7.95–7.87 (m, 2H), 7.56–7.27 (m, 9H), 5.25 (d, *J* = 3.4 Hz, 1H), 4.06 (q, *J* = 7.1 Hz, 2H), 1.14 (t, *J* = 7.0 Hz, 3H). ^13^C NMR (101 MHz, DMSO‐*d*_6_) *δ* 165.02, 152.26, 144.73, 143.21, 134.87, 133.66, 133.55, 132.03, 129.08, 128.82, 128.56, 128.23, 120.31, 101.76, 59.94, 53.43, 14.05. Spectroscopic data are in accordance with the literature [20]. MS (ESI) calculated for (C_22_H_17_Cl_2_N_2_O_3_)^−^ (M‐H)^−^ m/z = 415.07 found m/z = 415.04 (^35^Cl). HRMS m/z calculated for C_21_H_19_Cl_2_N_2_O_3_^+^ (M + H)^+^ 417.0767, found 417.0766.

#### 4.1.7 Ethyl (E)‐4‐(4‐methoxyphenyl)‐6‐(4‐methylstyryl)‐2‐oxo‐1,2,3,4‐tetrahydropyrimidine‐5‐carboxylate (16)

Following the general procedure, derivative **3** (233 mg, 0.8 mmol) and *p*‐tolualdehyde **6** (189 μL, 1.6 mmol) were reacted for 3 days to afford styryl derivative **16** as a white solid (90 mg, 29% yield). Rf = 0.60 (PE/EA 4:6). ^1^H NMR (400 MHz, DMSO‐*d*_6_) *δ* 9.15 (s, 1H), 7.89 (d, *J* = 16.6 Hz, 1H), 7.78 (s, 1H), 7.48–7.36 (m, 3H), 7.27–7.15 (m, 4H), 6.89 (d, *J* = 8.2 Hz, 2H), 5.20 (s, 1H), 4.06 (q, *J* = 7,1 Hz, 2H), 3.72 (s, 3H), 2.32 (s, 3H), 1.15 (t, *J* = 7.1 Hz, 3H). ^13^C NMR (101 MHz, DMSO‐*d*_6_) *δ* 165.27, 158.59, 152.54, 144.52, 138.83, 136.53, 134.65, 133.28, 129.63, 127.46, 127.12, 118.57, 113.85, 102.02, 59.77, 55.11, 53.36, 20.96, 14.11. MS (ESI) calculated for (C_23_H_25_N_2_O_4_)^+^ (M + H)^+^ m/z = 393.17, found m/z = 393.16. HRMS m/z calculated for (C_23_H_25_N_2_O_4_)^+^ (M + H)^+^ 393.1809, found 393.1807.

#### 4.1.8 Ethyl (E)‐4‐(4‐methoxyphenyl)‐6‐(4‐methoxystyryl)‐2‐oxo‐1,2,3,4‐tetrahydropyrimidine‐5‐carboxylate (17)

Following the general procedure, derivative **3** (192 mg, 0.6 mmol) and anisaldehyde **7** (147 μL, 1,2 mmol) were reacted for 3 days to afford styryl derivative **17** as a white solid (97 mg, 30% yield). Rf = 0.45 (PE/EA 4:6). ^1^H NMR (400 MHz, DMSO‐*d*_6_) *δ* 9.11 (s, 1H), 7.81 (d, *J* = 16.7 Hz, 1H), 7.77 (s, 1H), 7.49–7.38 (m, 3H), 7.19 (d, *J* = 8.4 Hz, 2H), 6.99 (d, *J* = 8.4 Hz, 2H), 6.89 (d, *J* = 8.4 Hz, 2H), 5.19 (d, *J* = 3.4 Hz, 1H), 4.07 (q, *J* = 7.0 Hz, 2H), 3.79 (s, 3H), 3.72 (s, 3H), 1.16 (t, *J* = 7.0 Hz, 3H). ^13^C NMR (101 MHz, DMSO‐*d*_6_) *δ* 165.31, 160.12, 158.56, 152.57, 144.75, 136.59, 134.44, 128.67, 128.60, 127.44, 117.18, 114.50, 113.82, 101.52, 59.70, 55.27, 55.09, 53.33, 14.13. MS (ESI) calculated for (C_23_H_25_N_2_O_5_)^+^ (M + H)^+^ m/z = 409.17, found m/z = 409.27. HRMS m/z calculated for (C_23_H_25_N_2_O_5_)^+^ (M + H)^+^ 409.1758, found 409.1760.

#### 4.1.9 Ethyl (E)‐6‐(4‐chlorostyryl)‐4‐(4‐methoxyphenyl)‐2‐oxo‐1,2,3,4‐tetrahydropyrimidine‐5‐carboxylate (18)

Following the general procedure, derivative **3** (233 mg, 0.8 mmol) and 4‐chloro benzaldehyde **8** (225 mg, 1,2 mmol) were reacted for 3 days to afford styryl derivative **18** as a white solid (90 mg, 27% yield). Rf = 0.44 (PE/EA 4:6). ^1^H NMR (400 MHz, DMSO‐*d*_6_) *δ* 9.18 (s, 1H), 7.92 (d, *J* = 16.7 Hz, 1H), 7.80 (s, 1H), 7.57–7.38 (m, 5H), 7.19 (d, *J* = 8.3 Hz, 2H), 6.90 (d, *J* = 8.3 Hz, 2H), 5.20 (d, *J* = 3.4 Hz, 1H), 4.06 (q, *J* = 6.8 Hz, 2H), 3.72 (s, 3H), 1.15 (t, *J* = 7.0 Hz, 3H). ^13^C NMR (101 MHz, DMSO‐*d*_6_) *δ* 165.20, 158.63, 152.48, 144.10, 136.43, 134.97, 133.47, 133.30, 129.08, 128.79, 127.49, 120.46, 113.88, 102.67, 59.87, 55.12, 53.42, 14.09. MS (ESI) calculated for (C_22_H_20_ClN_2_O_4_)^−^ (M‐H)^−^ m/z = 411.12, found m/z = 411.16. HRMS m/z calculated for C_22_H_22_ClN_2_O_4_^+^ (M + H)^+^ 413.1263, found 413.1264.

#### 4.1.10 Ethyl (E)‐6‐(4‐fluorostyryl)‐2‐oxo‐4‐phenyl‐1,2,3,4‐tetrahydropyrimidine‐5‐carboxylate (19)

Following the general procedure, derivative **1** (208 mg, 0.8 mmol) and 4‐fluoro benzaldehyde **9** (172 μL, 1,6 mmol) were reacted for 3 days to afford styryl derivative **19** as a white solid (104 mg, 36% yield). Rf = 0.55 (PE/EA 4:6). ^1^H NMR (400 MHz, DMSO‐*d*_6_) *δ* 9.20 (s, 1H), 7.90 – 7.82 (m, 2H), 7.60–7.53 (m, 2H), 7.45 (d, *J* = 16.7 Hz, 1H), 7.38 – 7.22 (m, 7H), 5.26 (d, *J* = 3.4 Hz, 1H), 4.06 (q, *J* = 7.1 Hz, 2H), 1.15 (t, *J* = 7.1 Hz, 3H). ^13^C NMR (101 MHz, DMSO‐*d*_6_) *δ* 165.20, 162.50 (d, *J* = 246.9 Hz), 152.51, 144.59, 144.33, 133.62, 132.60 (d, *J* = 3.0 Hz), 129.22 (d, *J* = 8.5 Hz), 128.55, 127.50, 126.29, 119.57 (d, *J* = 1.6 Hz), 116.02 (d, *J* = 21.8 Hz), 101.99, 59.85, 53.99, 14.07. ^19^F NMR (377 MHz, DMSO‐*d*_6_) *δ* −112.03. MS (ESI) calculated for (C_21_H_20_FN_2_O_3_)^+^ (M + H)^+^ m/z = 367.14, found m/z = 367.15. HRMS m/z calculated for (C_21_H_20_FN_2_O_3_)^+^ (M + H)^+^ 367.1452, found 367.1452

#### 4.1.11 Ethyl (E)‐6‐(4‐fluorostyryl)‐2‐oxo‐4‐(p‐tolyl)‐1,2,3,4‐tetrahydropyrimidine‐5‐carboxylate (20)

Following the general method procedure, derivative **2** (220 mg, 0.8 mmol) and 4‐fluoro benzaldehyde **9** (172 μL, 1,6 mmol) were reacted for 3 days to afford styryl derivative **20** as a white solid (115 mg, 38% yield). Rf = 0.61 (PE/EA 4:6). ^1^H NMR (400 MHz, DMSO‐*d*_6_) *δ* 9.16 (s, 1H), 7.86 (d, *J* = 16.6 Hz, 1H), 7.81 (s, 1H), 7.60–7.53 (m, 2H), 7.44 (d, *J* = 16.7 Hz, 1H), 7.30–7.23 (m, 2H), 7.19–7.11 (m, 4H), 5.21 (d, *J* = 3.4 Hz, 1H), 4.06 (q, *J* = 6.9 Hz, 2H), 2.27 (s, 3H), 1.15 (t, *J* = 7.0 Hz, 3H). ^13^C NMR (101 MHz, DMSO‐*d*_6_) *δ* 165.20, 162.47 (d, *J* = 246.8 Hz), 152.52, 144.40, 141.39, 136.64, 133.50, 132.61 (d, *J* = 3.1 Hz), 129.19 (d, *J* = 8.4 Hz), 129.03, 126.19, 119.57 (d, *J* = 1.9 Hz), 116.00 (d, *J* = 21.8 Hz), 102.15, 59.81, 53.67, 20.67, 14.09. ^19^F NMR (377 MHz, DMSO‐*d*_6_) *δ* −112.08. MS (ESI) calculated for (C_22_H_20_FN_2_O_3_)^−^ (M‐H)^−^ m/z = 379.15, found m/z = 379.29. HRMS m/z calculated for C_22_H_22_FN_2_O_3_^+^ (M + H)^+^ 381.1609, found 381.1610.

#### 4.1.12 Ethyl (E)‐6‐(4‐fluorostyryl)‐4‐(4‐methoxyphenyl)‐2‐oxo‐1,2,3,4‐tetrahydropyrimidine‐5‐carboxylate (21)

Following the general procedure, derivative **3** (232 mg, 0.8 mmol) and 4‐fluoro benzaldehyde **9** (172 μL, 1,6 mmol) were reacted for 3 days to afford styryl derivative **21** as a white solid (70 mg, 22% yield). Rf = 0.55 (PE/EA 4:6). ^1^H NMR (400 MHz, DMSO‐*d*_6_) *δ* 9.15 (s, 1H), 7.86 (d, *J* = 16.7 Hz, 1H), 7.79 (s, 1H), 7.60–7.52 (m, 2H), 7.44 (d, *J* = 16.7 Hz, 1H), 7.31–7.23 (m, 2H), 7.19 (d, *J* = 8.4 Hz, 2H), 6.90 (d, *J* = 8.3 Hz, 2H), 5.20 (d, *J* = 3.4 Hz, 1H), 4.06 (q, *J* = 7.0 Hz, 2H), 3.72 (s, 3H), 1.15 (t, *J* = 7.1 Hz, 3H). ^13^C NMR (101 MHz, DMSO‐*d*_6_) *δ* 165.21, 162.46 (d, *J* = 246.8 Hz), 158.59, 152.48, 144.25, 136.46, 133.45, 132.62 (d, *J* = 3.2 Hz), 129.17 (d, *J* = 8.5 Hz), 127.45, 119.58 (d, *J* = 1.5 Hz), 115.99 (d, *J* = 21.8 Hz), 113.84, 102.31, 59.79, 55.09, 53.38, 14.08. ^19^F NMR (377 MHz, DMSO‐*d*_6_) *δ* −112.10. MS (ESI) calculated for (C_22_H_20_FN_2_O_4_)^−^ (M‐H)^−^ m/z = 395.15, found m/z = 395.19. HRMS m/z calculated for C_22_H_22_FN_2_O_4_^+^ (M + H)^+^ 397.1558, found 397.1559.

### 4.2 Cell Culture, Cell Viability and Cytotoxicity Assays

HeLa human cervix cancer cell line and MCF‐7 human breast cancer cell line were maintained and used with DMEM high glucose (D0822, Sigma–Aldrich) supplemented with 10% fetal bovine serum (F7524, Sigma–Aldrich), 1% P/S (P4333, Sigma–Aldrich). 10,000 cells per well of HeLa and MCF‐7 cells were seeded into a 96‐well plate as triplicate wells for MTT assay and LDH assay [21].

Cell viability was measured by MTT assay (M6494, ThermoFisher) and cytotoxicity was measured by LDH assay kit (ab65393, Abcam) by following the manufacturer's instructions. Optical density (O.D) had been enumerated with a microplate reader (Hidex Sense Meta Plus).

#### 4.2.1 Western Blot Analysis

HeLa and MCF‐7 cells were seeded into a 6well plate at 500 000 cells per well. After 2 days of 10 µM styryl derivative treatment, lysates were harvested with CelLytic M (C2978, Sigma–Aldrich). 20 µg lysate was prepared with 2x Laemmli Sample Buffer (1610737, Biorad). SDS PAGE were performed using Mini‐PROTEAN TGX Precast Gels (Bio‐Rad) and transferred using Trans‐Blot Turbo Transfer System (Bio‐Rad). Primary antibody, PARP (95425, Cell signaling), Caspase 3 (ab32042, Abcam), GAPDH (ab8245, Abcam), p53 (ab32389, Abcam), p21 (ab109199, Abcam), c‐Myc (ab32072, Abcam), β‐actin (ab8227, Abcam), lamin and α‐tubulin (ab7291, Abcam) were blotted for overnight and Goat Anti‐Rabbit HRP (ab205718) and goat antimouse IgG‐HRP (sc2005, Santa Cruz Biotechnology, Inc.) were blotted for secondary antibody 1 h. ImageQuantTMLAS 500 (29‐0050‐63, GE) has been used to detect protein bands.

### 4.3 Evaluation of Physico‐Chemical Properties

#### 4.3.1 Material and Methods

Diclofenac, progesterone, nicardipine, erythromycin, chlorambucil, and desipramine HCl were obtained from Sigma (St. Louis, MO, USA). Pepsin and trypsin were purchased from Sigma and Gibco, respectively. Ammonium formate, zinc sulfate, NaH_2_PO_4_ · H_2_O, Na_2_HPO_4_ · 7H_2_O, HCl, NaOH, formic acid, and 1‐octanol were supplied by Merck (Darmstadt, Germany). DMSO, ethanol, acetonitrile, and methanol (LC–MS grade) were purchased from Merck and Isolab (Germany). All reagents were of analytical grade. Analyses were performed using an Agilent 1290 UPLC coupled with an Agilent 6475 triple quadrupole MS and ZORBAX RRHD C18 analytical columns. Supporting equipment included an Eppendorf Thermomixer Comfort incubator, Heidolph vortex and magnetic stirrer, DKsonic water bath, Mettler Toledo SD20KIT pH meter, Nüve NF800R refrigerated centrifuge, Radwag MYA 5.5Y microbalance, and Sartorius Entris 224I‐1S balance. Standard laboratory glassware, Eppendorf pipettes, PTFE‐L filters (0.22 μm), and storage units (Arçelik K78550NE refrigerator/freezer and B Medical Systems U501 ultralow freezer) were used throughout.

#### 4.3.2 Shake‐Flask Aqueous Solubility Assay

Calibration standards were prepared by serial dilution of 10 mM DMSO stock solutions to final concentrations of 100–0.1 μM. Standards were diluted with PBS and an internal standard solution (IS: 100 ng/mL desipramine in H_2_O/acetonitrile, 1:1, v/v), centrifuged (10 000 rpm, 15 min, RT), filtered (0.22 μm PTFE‐L), and analyzed by UPLC–MS/MS (injection volume: 5 μL). For kinetic solubility testing, 15 μL of 10 mM stock solution of each compound was mixed with 485 μL PBS (100 mM, pH 7.4) and incubated at 25°C with stirring (1100 rpm, 2 h). Samples were centrifuged and filtered as described above, diluted with IS‐1 (1 µg/mL desipramine in acetonitrile), and injected into the UPLC–MS/MS system. The procedure follows established shake‐flask protocols [22, 23]. Progesterone and diclofenac were included as positive controls for poorly and highly soluble compounds, respectively. All experiments were conducted in duplicate, and concentrations were determined from the calibration curve. Kinetic solubility was reported as the concentration of free drug dissolved in PBS.

#### 4.3.3 Lipophilicity (LogD) Determination

LogD determination was performed using 1 mM test solutions prepared from 10 mM stock solutions in a classic *n*‐octanol/PBS (1:1, v/v) shake‐flask system. Samples were equilibrated at 25°C with shaking (2000 rpm) for 2 h, followed by centrifugation to separate the aqueous (PBS) and organic (*n*‐octanol) phases. Aliquots from both phases were serially diluted with an internal standard solution (IS; 100 ng/mL desipramine in H_2_O/acetonitrile, 1:1, v/v) and analyzed by UPLC–MS/MS (injection volume 5 µL). LogD values were calculated based on the relative peak areas of the analyte in the octanol and PBS phases, corrected for the corresponding dilution factors, according to the following equation where DF = Diluition factor [24]:

$$\text{Log}\text{D}=\mathrm{Log}\left(\frac{\text{AREAOct }\times \text{DFOct}}{\text{AREABuf }\times \text{DFBuf}}\right)$$

All experiments were conducted in duplicate (*n* = 2), and dilution factors were included in the calculations. Nicardipine was employed as a positive control during the assay.

#### 4.3.4 In Vitro Stability Studies

In vitro stability experiments in SGF, SIF, and plasma were performed with minor modifications to previously reported methods [25]. SGF and SIF media were prepared according to USP guidelines (United States Pharmacopeial Convention, 2017). SGF was obtained by dissolving 0.5 g of pepsin (≥250 units/mg) in dilute HCl (pH 1.5) to a final volume of 50 mL in a volumetric flask, whereas SIF was prepared by dissolving 0.5 g of trypsin (1:250) in phosphate‐buffered saline (PBS, pH 6.8) to a final volume of 50 mL. Commercially available human plasma was used for plasma stability studies. SGF, SIF, and plasma samples were aliquoted as 199 µL into 2 mL Eppendorf tubes. A 1 mM test solution was prepared by diluting the 10 mM stock solution with DMSO. Stability studies were carried out at two points (0 and 120 min). Aliquots of plasma/SGF/SIF were preincubated at 37°C for 15 min on a heated shaker, after which 1 µL of the 1 mM test solution was added to each tube. For the 0 min samples, 600 µL of ice‐cold IS‐1 solution (1 µg/mL desipramine in acetonitrile) was added, followed by mixing at 2000 rpm for 10 min and centrifugation at 10 000 rpm for 30 min at + 4°C. From the resulting supernatant, 100 µL was transferred into 1000 µL of IS‐2 solution (100 ng/mL desipramine in water/acetonitrile, 1:1), mixed for 30 s, and analyzed by UPLC‐MS/MS. For the 120 min samples, incubation was performed at 37°C without agitation for 120 min, after which the same processing steps as described for the 0 min samples were applied. All experiments were performed in duplicate (*n* = 2). Propantheline was used as a positive control for plasma stability studies, erythromycin for SGF studies, and chlorambucil for SIF studies.

### 4.4 Molecular Docking Studies

The 3D X‐ray structure of kinesin‐like protein KIF11 (i.e., Eg5) was retrained from the Protein Data Bank (PDB) (entry 1Q0B [19], resolution 1.90 Å). MZDock software pipeline was employed for: (a) energy minimization and refinement of the protein structure; (b) energy minimization of the **16** and **17**; (c) redocking studies on monastrol for setting the coordinates of the grid box; (d) molecular docking analysis with standard parameters; and (e) inspection of the main interactions of the protein‐ligand docking complexes [18]. The protein was thus prepared by adding polar hydrogens, Kollman charges, fixing missing atoms and removing all water molecules. The 3D conformation of **16** and **17** were processed in order to properly generate possible stereoisomers and protonation states at a physiological pH equal to 7.4. Energy minimization was applied by using MMFF94 force fields. The binding site grid box was set up on the centroid of the cocrystal ligand monastrol with a buffer space of 4 Å. Finally, docking simulations were carried out by using Smina‐Vina scoring function available in MzDOCK. Specifically, the standard docking protocol with default settings has been employed (i.e., num_modes and exhaustiveness set to 9 and 8, respectively). The in silico protocol was tested by redocking the cognate ligand monastrol into its corresponding binding site. Pymol is used as a molecular visualization tool as well as to analyze molecular interactions at the binding site.

### 4.5 Statistical Analysis

Statistical differences in cell viability between compound treatment groups and the control group were evaluated using Student's *t*‐test. Data are presented as mean ± standard deviation, and error bars in the graphs represent these values. *p*‐values below 0.05 were considered statistically significant and indicated by an asterisk (*).

## Supporting Information

The authors have cited additional references within the Supporting Inmation [12]. Additional supporting information can be found online in the Supporting Information section. **Supporting** **Fig. S1**: ^1^H NMR of **1** in DMSO‐d6. **Supporting** **Fig. S2**: ^13^C NMR of **1** in DMSO‐d6. **Supporting** **Fig. S3**: ESI‐MS of **1**. **Supporting** **Fig. S4**: **^1^** H NMR of **2** in DMSO‐d6. **Supporting** **Fig. S5**: ^13^C NMR of **2** in DMSO‐d6. **Supporting** **Fig. S6**: ESI‐MS of **2**. **Supporting** **Fig. S7**: ^1^H NMR of **3** in DMSO‐d6. **Supporting** **Fig. S8**: ^13^C NMR of **3** in DMSO‐d6. **Supporting** **Fig. S9**: 2D NMR and assignment of 3. **Supporting** **Fig. S10**: ESI‐MS of **3**. **Supporting** **Fig. S11**: ^1^H NMR of **4** in DMSO‐d6. **Supporting** **Fig. S12**: ^13^C NMR of **4** in DMSO‐d6. **Supporting** **Fig. S13**: ESI‐MS of **4**. **Supporting** **Fig. S14**: ^1^H NMR of **10** in DMSO‐d6. **Supporting** **Fig. S15**: ^13^C NMR of **10** in DMSO‐d6. **Supporting** **Fig. S16**: ESI‐MS of **10**. **Supporting** **Fig. S17**: ^1^H NMR of **11** in DMSO‐d6. **Supporting** **Fig. S18**: ^13^C NMR of **11** in DMSO‐d6. **Supporting** **Fig. S19**: ESI‐MS of **11**. **Supporting** **Fig. S20**: ^1^H NMR of **12** in DMSO‐d6. **Supporting** **Fig. S21**: ^13^C NMR of **12** in DMSO‐d6. **Supporting** **Fig. S22**: ESI‐MS of **12**. **Supporting** **Fig. S23**: ^1^H NMR of **13** in DMSO‐d6. **Supporting** **Fig. S24**: ^13^C NMR of **13** in DMSO‐d6. **Supporting** **Fig. S25**: ESI‐MS of **13. Supporting** **Fig. S26**: ^1^H NMR of **14** in DMSO‐d6. **Supporting** **Fig. S27**: ^13^C NMR of **14** in DMSO‐d6. **Supporting** **Fig. S28**: ESI‐MS of **14. Supporting** **Fig. S29**: ^1^H NMR of **15** in DMSO‐d6. **Supporting** **Fig. S30**: ^13^C NMR of **15** in DMSO‐d6. **Supporting** **Fig. S31**: ESI‐MS of **15. Supporting** **Fig. S32**: ^1^H NMR of **16** in DMSO‐d6. **Supporting** **Fig. S33**: ^13^C NMR of **16** in DMSO‐d6. **Supporting** **Fig. S34**: ESI‐MS of **16. Supporting** **Fig. S35**: ^1^H NMR of **17** in DMSO‐d6. **Supporting** **Fig. S36**: ^13^C NMR of **17** in DMSO‐d6. **Supporting** **Fig. S37**: ESI‐MS of **17. Supporting** **Fig. S38**: ^1^H NMR of **18** in DMSO‐d6. **Supporting** **Fig. S39**: ^13^C NMR of **18** in DMSO‐d6. **Supporting** **Fig. S40**: ESI‐MS of **18. Supporting** **Fig. S41**: ^1^H NMR of **19** in DMSO‐d6. **Supporting** **Fig. S42**: ^13^C NMR of **19** in DMSO‐d6. **Supporting** **Fig. S43**: ^19^F NMR of **19** in DMSO‐d6. **Supporting** **Fig. S44**: ESI‐MS of **19**. **Supporting** **Fig. S45**: ^1^H NMR of **20** in DMSO‐d6. **Supporting** **Fig. S46**: ^13^C NMR of **20** in DMSO‐d6. **Supporting** **Fig. S47**: ^19^F NMR of **20** in DMSO‐d6. **Supporting** **Fig. S48**: ESI‐MS of **20. Supporting** **Fig. S49**: ^1^H NMR of **21** in DMSO‐d6. **Supporting** **Fig. S50**: ^13^C NMR of **21** in DMSO‐d6. **Supporting** **Fig. S51**: ^19^F NMR of **21** in DMSO‐d6. **Supporting** **Fig. S52**: ESI‐MS of **21** **.**

## Funding

The funding was provided by Italian Ministry of Research (FABR2017).

## Conflicts of Interest

The authors declare no conflicts of interest.

## Supporting information

Supplementary Material

## Data Availability

The data that support the findings of this study are available from the corresponding author upon reasonable request
