# Supplementary material for: Development of Styryl‐Modified 3,4‐Dihydropyrimidin‐2(1H)‐ones as Potential Antitumor Agents
Source: ChemMedChem. 2026 Apr 7;21(7):e202501073. doi: 10.1002/cmdc.202501073 (PMC13056349; doi:10.1002/cmdc.202501073)
Supplement: Supplementary file 1 — Supplementary Material [file CMDC-21-e202501073-s001.zip › Supporting Information 1_3_02_2026.pdf]

SUPPORTING INFORMATION 1

Development of Styryl-modified 3,4-Dihydropyrimidin-2(1*H*)-ones as Potential Antitumor Agents

*Konstantinos Panagoulias,[a] Dr. Woonghee Kim,[b] Dr. Murat Ozdemir,[c] Dr. Busra Turan,[c] Prof. Adil Mardinoglu,[b, d] Prof. Hasan Turkez,[e] Daniela Trisciuzzi,[f] Orazio Nicolotti,[f] Prof. Antonio Di Stefano,[g] Prof. Stamatia Vassiliou**[a] and Prof. Ivana Cacciatore,* [g]*

[a] K. Panagoulias, Prof. Stamatia Vassiliou**
Laboratory of Organic Chemistry, Department of Chemistry
National and Kapodistrian University of Athens
Athens, GREECE
E-mail: kostas.panagoulias@protonmail.com, svassiliou@chem.uoa.gr

[b] Dr. Woonghee Kim, Prof. Adil Mardinoglu
Science for Life Laboratory
KTH-Royal Institute of Technology,
SE-17121 Stockholm, SWEDEN
E-mail: woonghee.kim@scilifelab.se, adilm@scilifelab.se

[c] Dr. Murat Ozdemir, Dr. Busra Turan
Trustlife Laboratories
Drug Research & Development Center
34774, Istanbul, TURKEY
E-mail: murat.ozdemir@trustlifelabs.com, busra.turan@trustlifelabs.com

[d] Prof. Adil Mardinoglu
Centre for Host-Microbiome Interactions
Faculty of Dentistry, Oral & Craniofacial Sciences
King's College London
London SE1 9RT, UNITED KINGDOM
E-mail: adilm@scilifelab.se

[e] Prof. Hasan Turkez
Department of Medical Biology, Faculty of Medicine
Atatürk University
Erzurum, TURKEY
E-mail: hasan.turkez@gmail.com

[f] Prof. Daniela Trisciuzzi, Prof. Orazio Nicolotti
Department of Pharmacy, Pharmaceutical Sciences,
University of Bari "Aldo Moro"

Bari, ITALY
E-mail: daniela.trisciuzzi@uniba.it, orazio.nicolotti@uniba.it

[g] Prof. Antonio Di Stefano, Prof. Ivana Cacciatore*
Department of Pharmacy
"G. D'Annunzio" University of Chieti-Pescara
66100, Chieti Scalo (Chieti), ITALY
E-mail: antonio.distefano@unich.it, ivana.cacciatore@unich.it

NMR and MS spectra of 1-12

General method for the synthesis of 3,4-dihydropyrimidin-2(1*H*)-ones (1-4), A

To a stirred solution of the appropriate aldehyde (10 mmol) in abs EtOH (20 mL), ethyl acetoacetate (1.27 mL, 10 mmol), urea (0.90 g, 15 mmol), cat. $FeCl_3.6H_2O$ (0.676 g, 2.5 mmol) and conc. HCl (1-2 drops) were added. The reaction mixture was heated to 75° C for 5 hours. The reaction mixture was poured onto 100 g ice/water and the precipitate was stirred for 2 hours. The precipitate was filtered and washed with H_2O and 95% EtOH. The solid was stirred in EtOH (20 mL) at 60°C for 1 hour and filtered. The solid product was dried over P_2O_5.

Ethyl-6-methyl-4-phenyl-2-oxo-1,2,3,4-tetrahydropyrimidine-5-carboxylate, 1

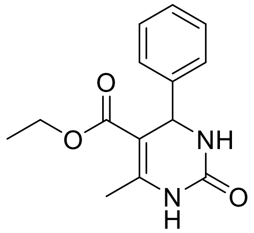

$C_{14}H_{16}N_2O_3$
Mol. Wt.: 260,2884

Following the general method **A** using benzaldehyde (1 mL, 10 mmol), compound **1** was obtained as white solid in 60% yield. Rf = 0.62 (PE/EA 1:1).

^{1}H NMR (400 MHz, DMSO-*d*6) δ 9.18 (s, 1H), 7.73 (s, 1H), 7.42 – 7.13 (m, 5H), 5.15 (s, 1H), 3.98 (q, *J* = 7.3 Hz, 2H), 2.25 (s, 3H), 1.09 (t, *J* = 7.1 Hz, 3H).

^{13}C NMR (101 MHz, DMSO-*d*6) δ 165.35, 152.15, 148.37, 144.88, 128.40, 127.28, 126.26, 99.27, 59.20, 53.97, 17.79, 14.08.

Spectroscopic data are in accordance with the literature[11].

MS (ESI) calculated for $C_{14}H_{17}O_3N_2^+$ $(M+H)^+$ m/z = 261.12 found m/z = 261.24.

HRMS m/z calculated for $C_{14}H_{17}N_2O_3^+$ [M+H] 261.1234, found 261.1234

Ethyl-6-methyl-4-(4-methylphenyl)-2-oxo-1,2,3,4-tetrahydropyrimidine-5-carboxylate, 2

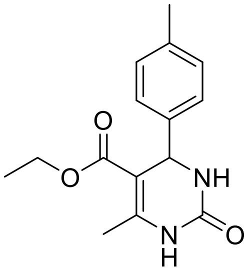

$C_{15}H_{18}N_2O_3$
Mol. Wt.: 274.32

Following the general method **A** using p-tolualdehyde (1.18 mL, 10 mmol) compound **2** was obtained as white solid in 64% yield. Rf = 0.63 (PE/EA 1:1).

^{1}H NMR (400 MHz, DMSO-d_6) δ 9.14 (s, 1H), 7.68 (s, 1H), 7.12 (s, 4H), 5.11 (s, 1H), 3.98 (q, J = 7.0 Hz, 2H), 2.26 (s, 3H), 2.24 (s, 3H), 1.10 (t, J = 7.0 Hz, 3H).

^{13}C NMR (101 MHz, DMSO-d_6) δ 165.37, 152.19, 148.16, 141.97, 136.37, 128.90, 126.15, 99.42, 59.16, 53.63, 20.65, 17.76, 14.10.

Spectroscopic data are in accordance with the literature [24].

MS (ESI) calculated for $C_{15}H_{19}N_2O_3^+$ $(M+H)^+$ m/z = 275.13 found m/z = 275.24.

HRMS m/z calculated for $C_{15}H_{19}N_2O_3^+$ [M+H] 275.1390, found 275.1391

Ethyl-6-methyl-4-(4-methoxyphenyl)-2-oxo-1,2,3,4-tetrahydropyrimidine-5-carboxylate, 3

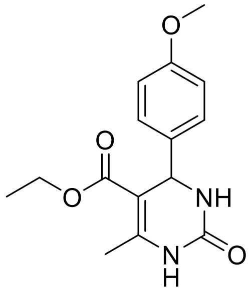

$C_{15}H_{18}N_2O_4$
Mol. Wt.: 290.31

Following the general method **A** using anisaldehyde (1.22 mL, 10 mmol) compound **3** was obtained as white solid in 64% yield. Rf = 0.65 (PE/EA 1:1).

^{1}H NMR (400 MHz, DMSO-d_6) δ 9.14 (s, 1H), 7.66 (s, 1H), 7.15 (d, J = 8.2 Hz, 2H), 6.87 (d, J = 8.2 Hz, 2H), 5.09 (s, 1H), 3.98 (q, J = 7.1 Hz, 2H), 3.72 (s, 3H), 2.24 (s, 3H), 1.10 (t, J = 7.1 Hz, 3H).

^{13}C NMR (101 MHz, DMSO-d_6) δ 165.39, 158.46, 152.18, 148.02, 137.07, 127.41, 113.71, 99.59, 59.16, 55.06, 53.35, 17.77, 14.11.

MS (ESI) calculated for $C_{15}H_{19}N_2O_4^+$ $(M+H)^+$ m/z = 291.13 found m/z = 291.25.

HRMS m/z calculated for $C_{15}H_{19}N_2O_4^+$ [M+H] 291.1339, found 291.1339

Ethyl-6-methyl-4-(4-chlorophenyl)-2-oxo-1,2,3,4-tetrahydropyrimidine-5-carboxylate, 4

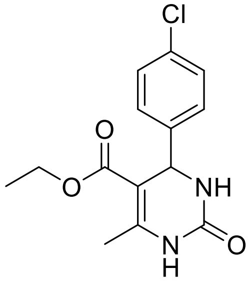

$C_{14}H_{15}ClN_2O_3$
Mol. Wt.: 294.73

Following the general method **A** using 4-chlorobenzaldehyde (2.11 g, 15 mmol) compound **4** was obtained as white solid in 70% yield.

Rf = 0.46 (PE/EA 6:4).

^{1}H NMR (400 MHz, DMSO-d_6) δ 9.24 (s, 1H), 7.77 (s, 1H), 7.39 (d, J = 8.1 Hz, 2H), 7.24 (d, J = 8.2 Hz, 2H), 5.14 (d, J = 3.4 Hz, 1H), 3.98 (q, J = 7.1 Hz, 2H), 2.25 (s, 3H), 1.09 (t, J = 7.1 Hz, 3H).

^{13}C NMR (101 MHz, DMSO-d_6) δ 165.21, 151.94, 148.73, 143.80, 131.78, 128.40, 128.19, 98.83, 59.26, 53.42, 17.80, 14.07.

Spectroscopic data are in accordance with the literature [24].

MS (ESI) calculated for $C_{14}H_{16}ClN_2O_3^+$ $(M+H)^+$ m/z = 295.08 found m/z = 295.27.

HRMS m/z calculated for $C_{14}H_{16}ClN_2O_3^+$ [M+H] 295.0844, found 295.0845

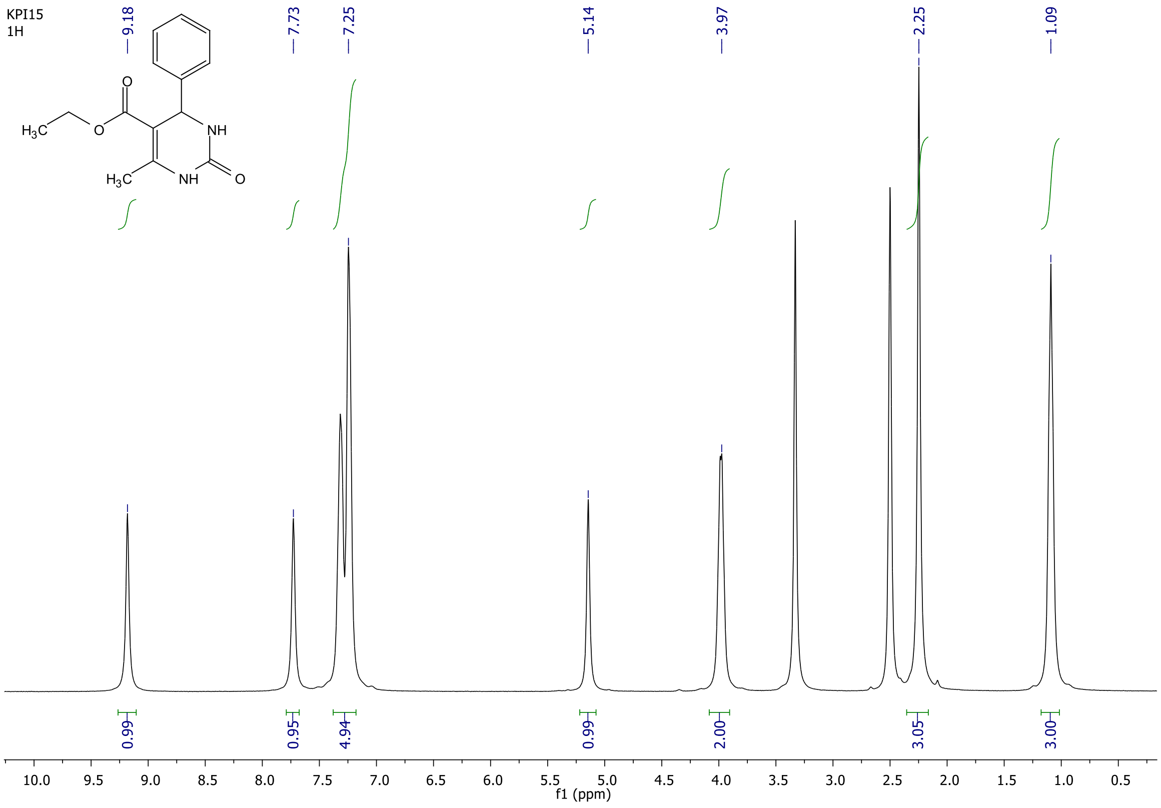

Figure 1. ^{1}H NMR of **1** in DMSO-d6

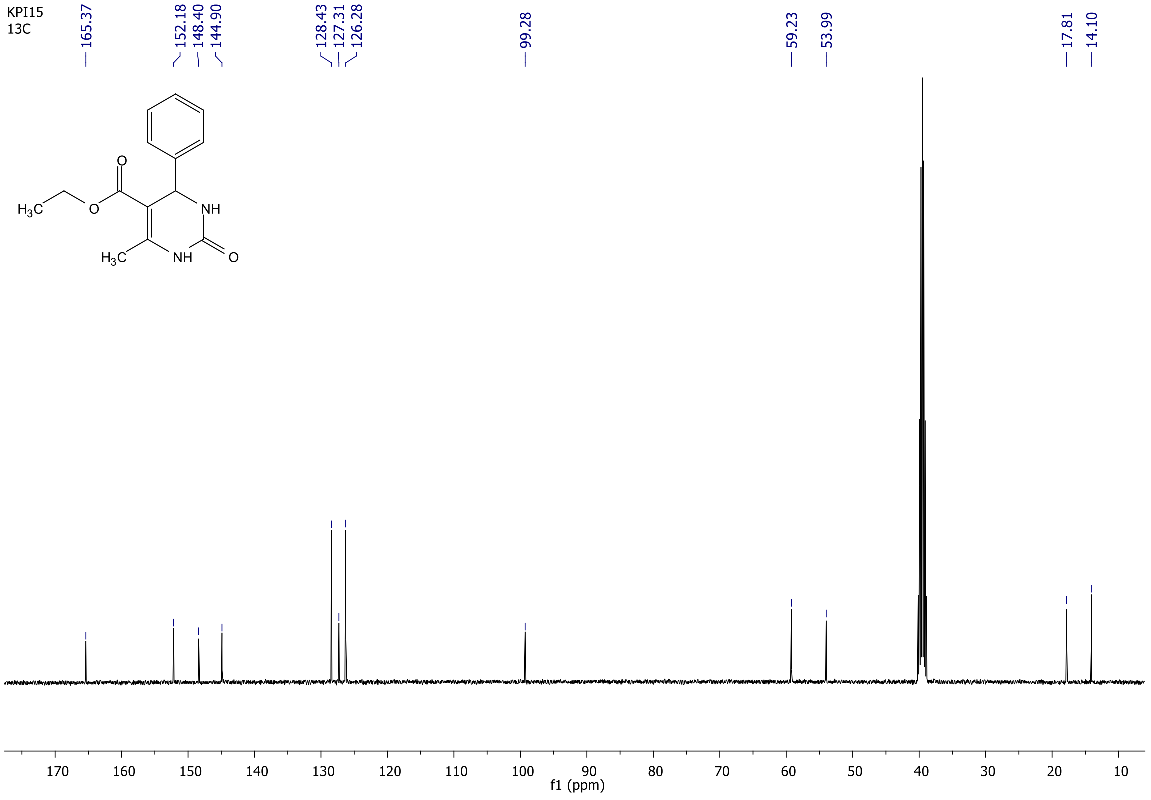

Figure 2. ^{13}C NMR of **1** in DMSO-d6

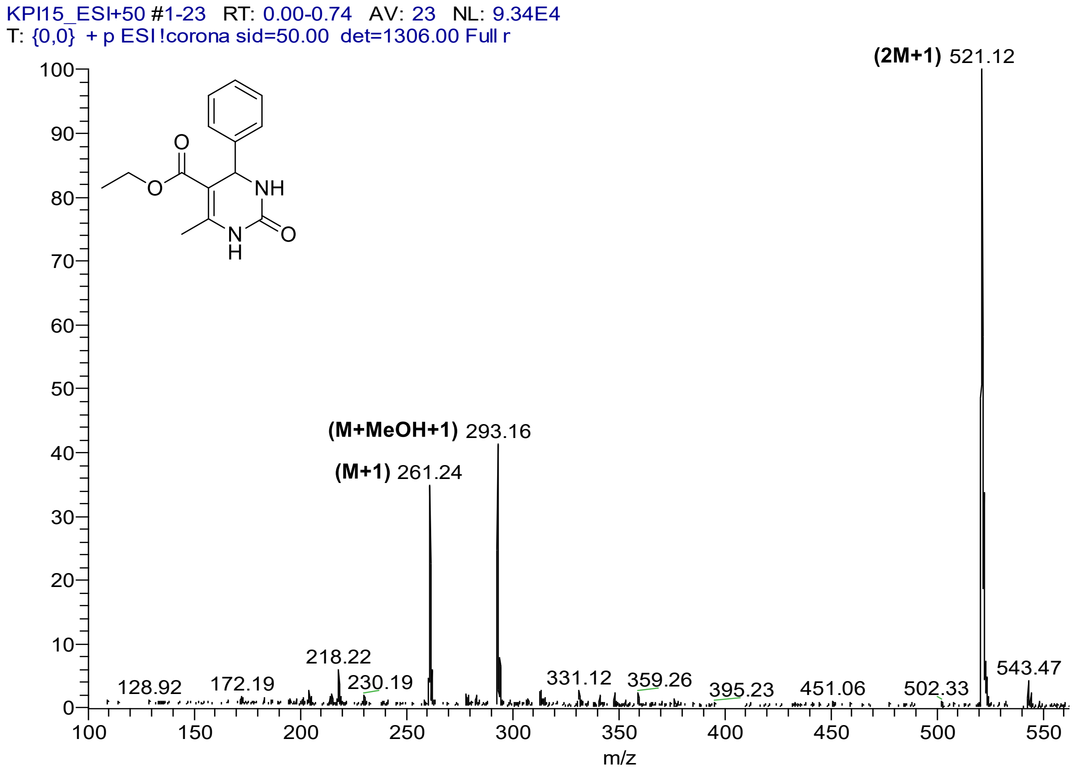

Figure 3. ESI-MS of **1.**

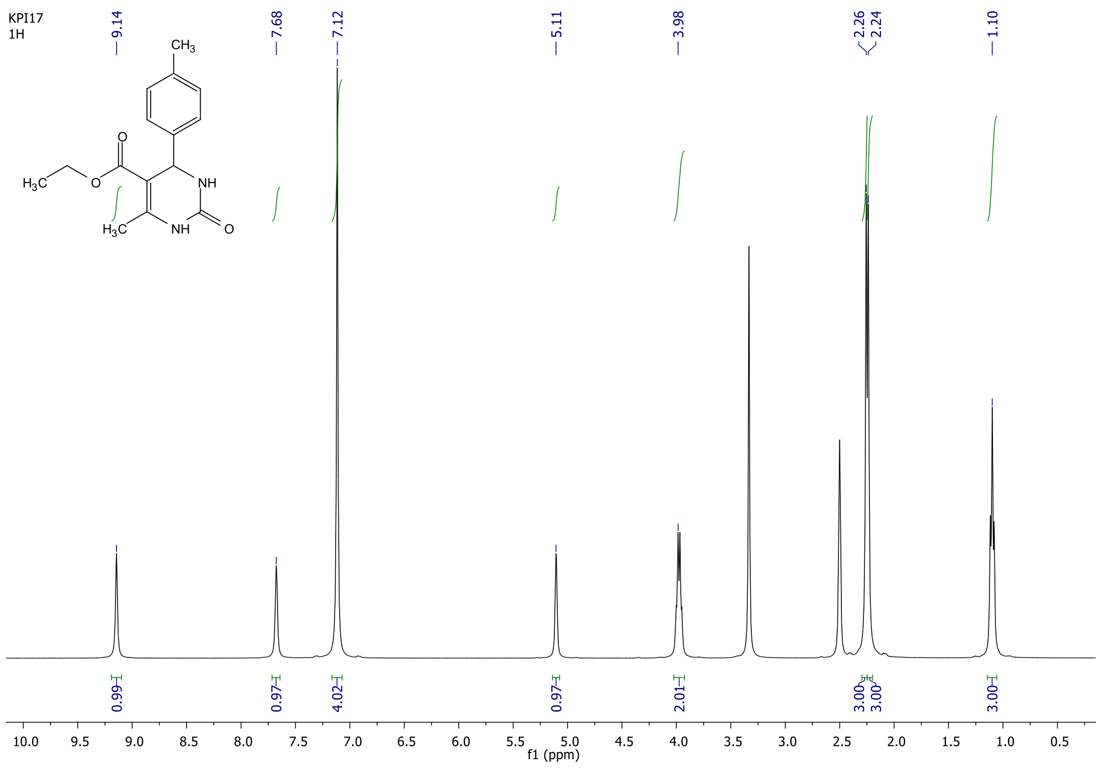

Figure 4. ^{1}H NMR of **2** in DMSO-d6

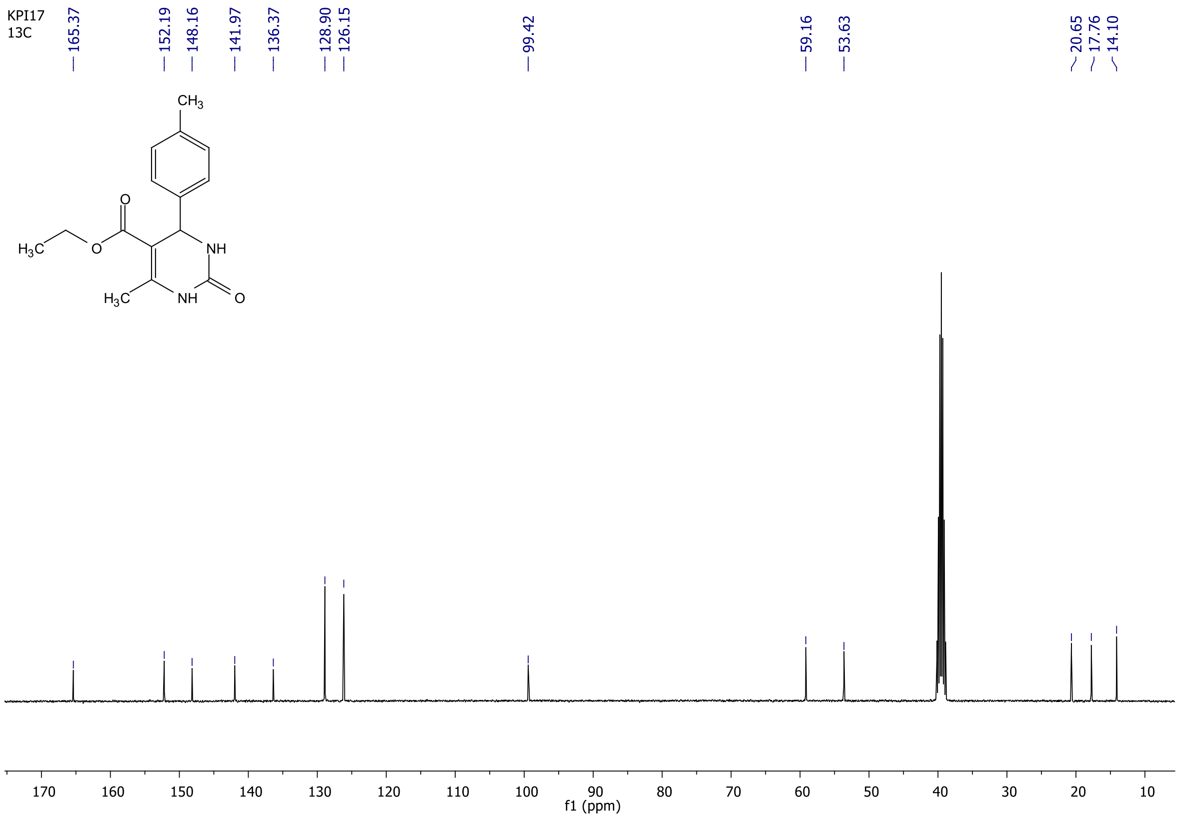

Figure 5. ^{13}C NMR of **2** in DMSO-d6

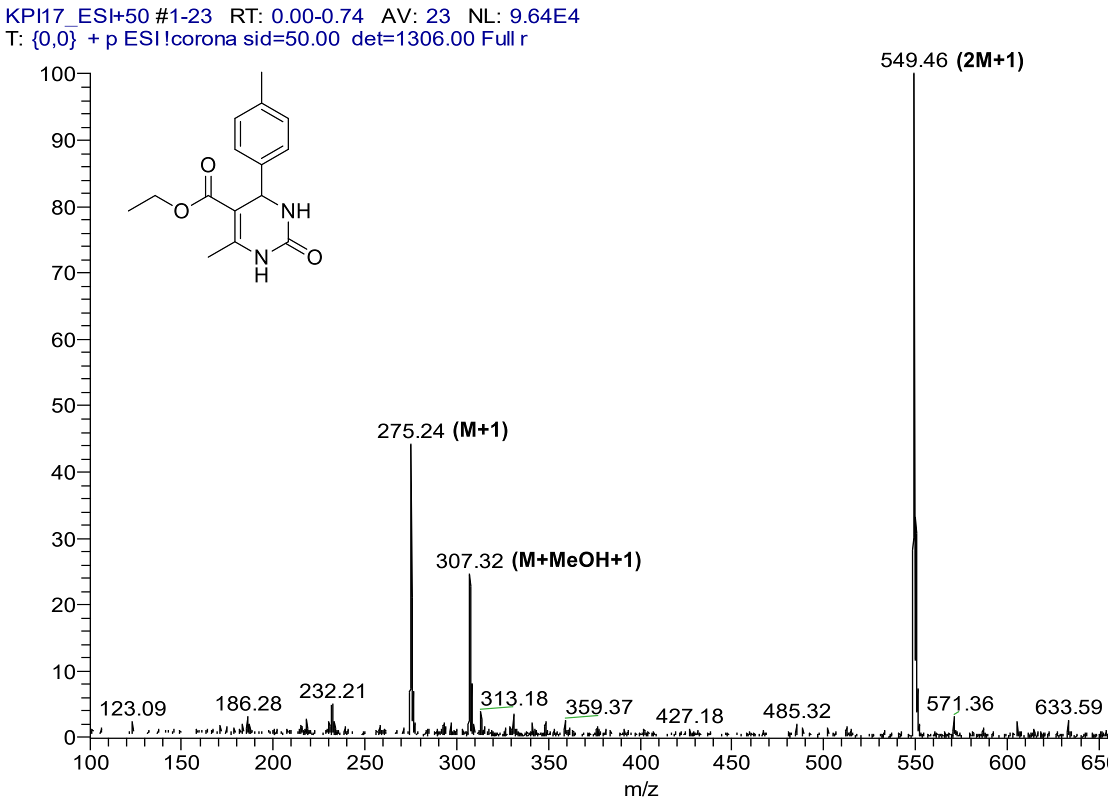

Figure 6. ESI-MS of **2**

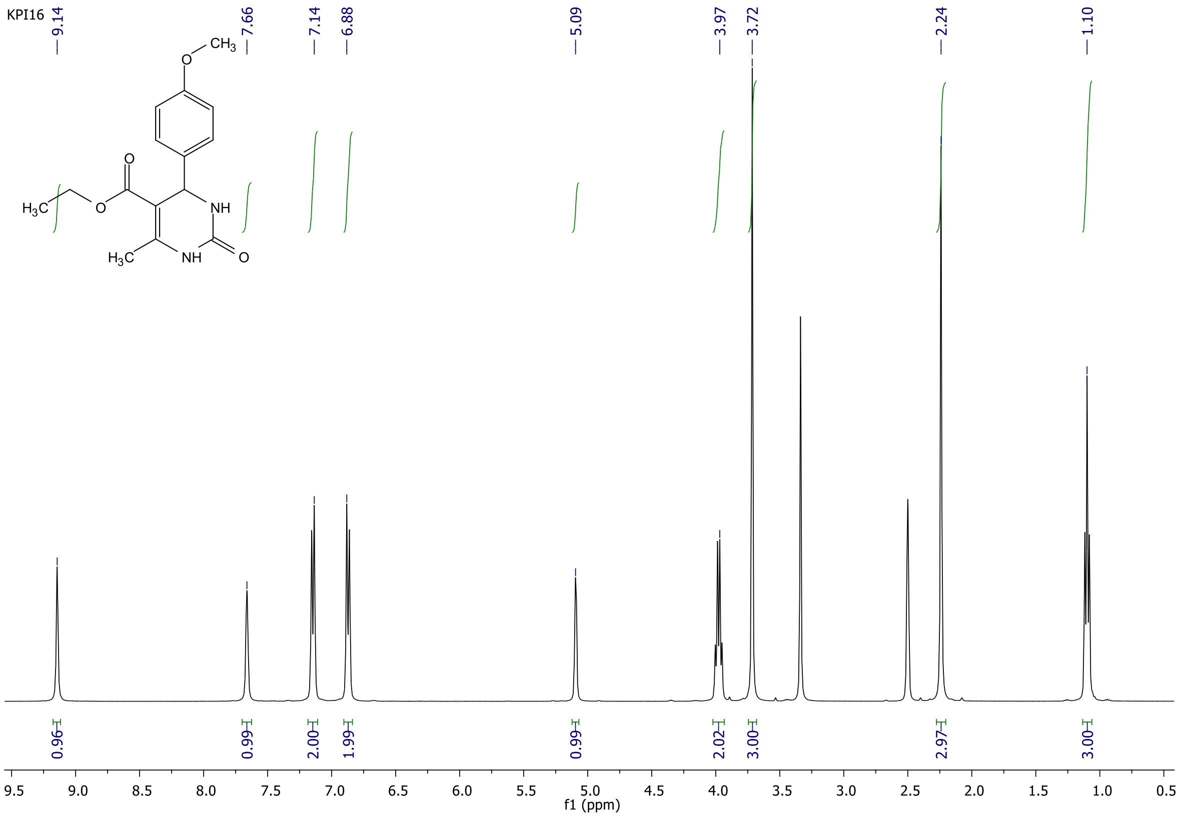

Figure 7. ^{1}H NMR of **3** in DMSO-d6

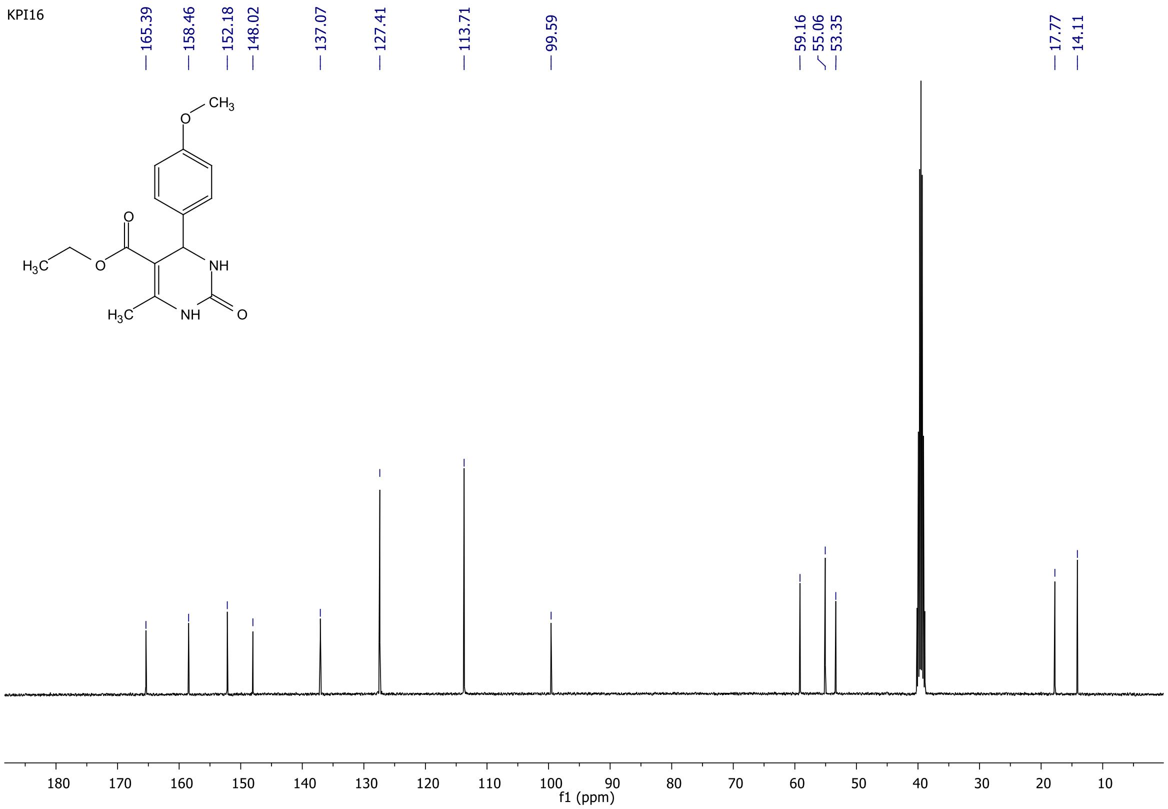

Figure 9. ^{13}C NMR of **3** in DMSO-d6

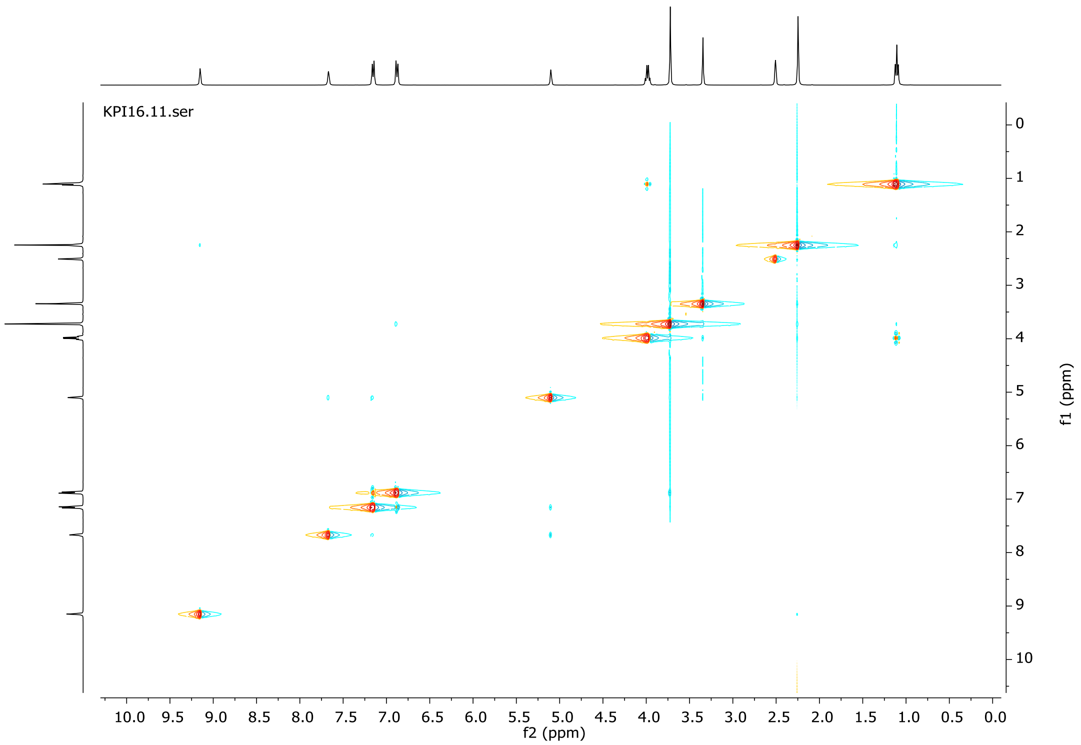
KPI16.11.ser
f1 (ppm)
f2 (ppm)
0
1
2
3
4
5
6
7
8
9
10
10.0 9.5 9.0 8.5 8.0 7.5 7.0 6.5 6.0 5.5 5.0 4.5 4.0 3.5 3.0 2.5 2.0 1.5 1.0 0.5 0.0

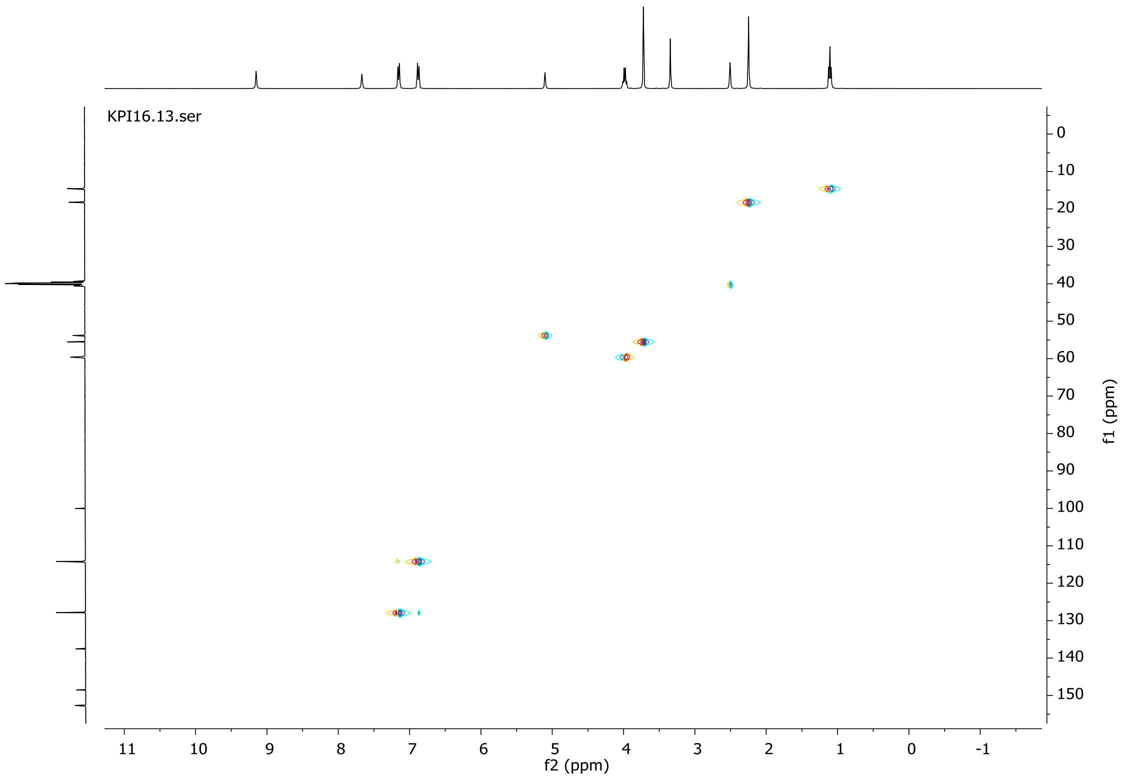
KPI16.13.ser
f1 (ppm)
f2 (ppm)
0
10
20
30
40
50
60
70
80
90
100
110
120
130
140
150
11 10 9 8 7 6 5 4 3 2 1 0 -1

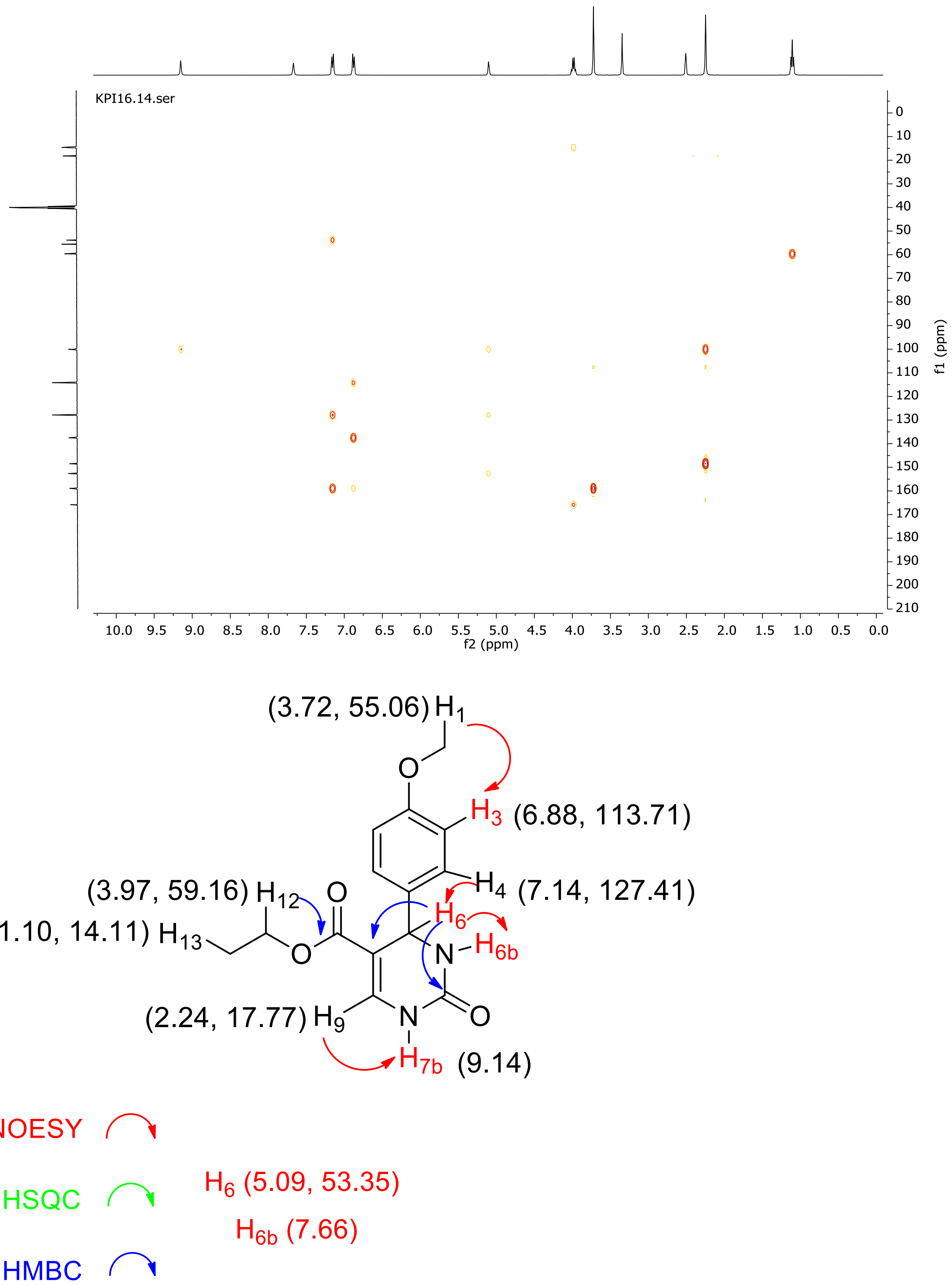

Figure 9. 2D NMR and assignment of **3**

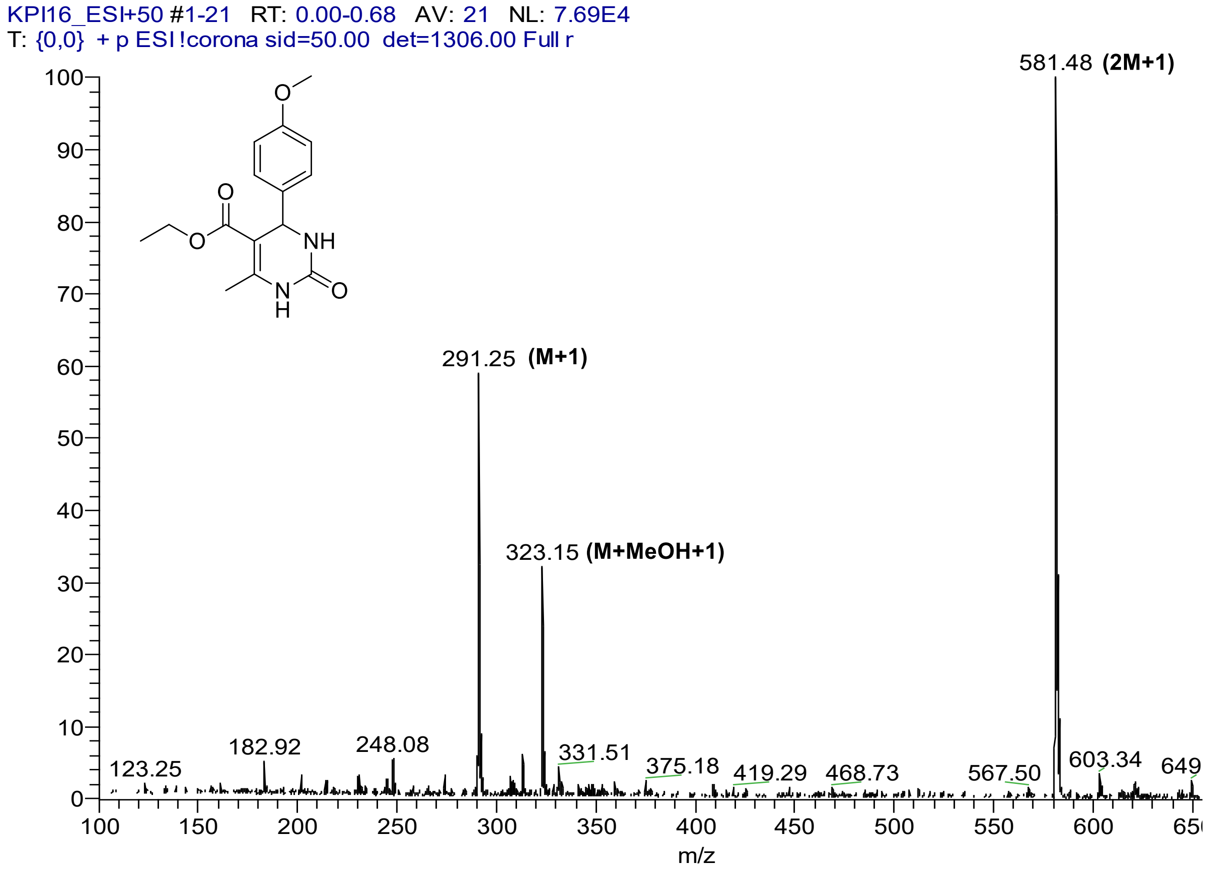

Figure 10. ESI-MS of **3**

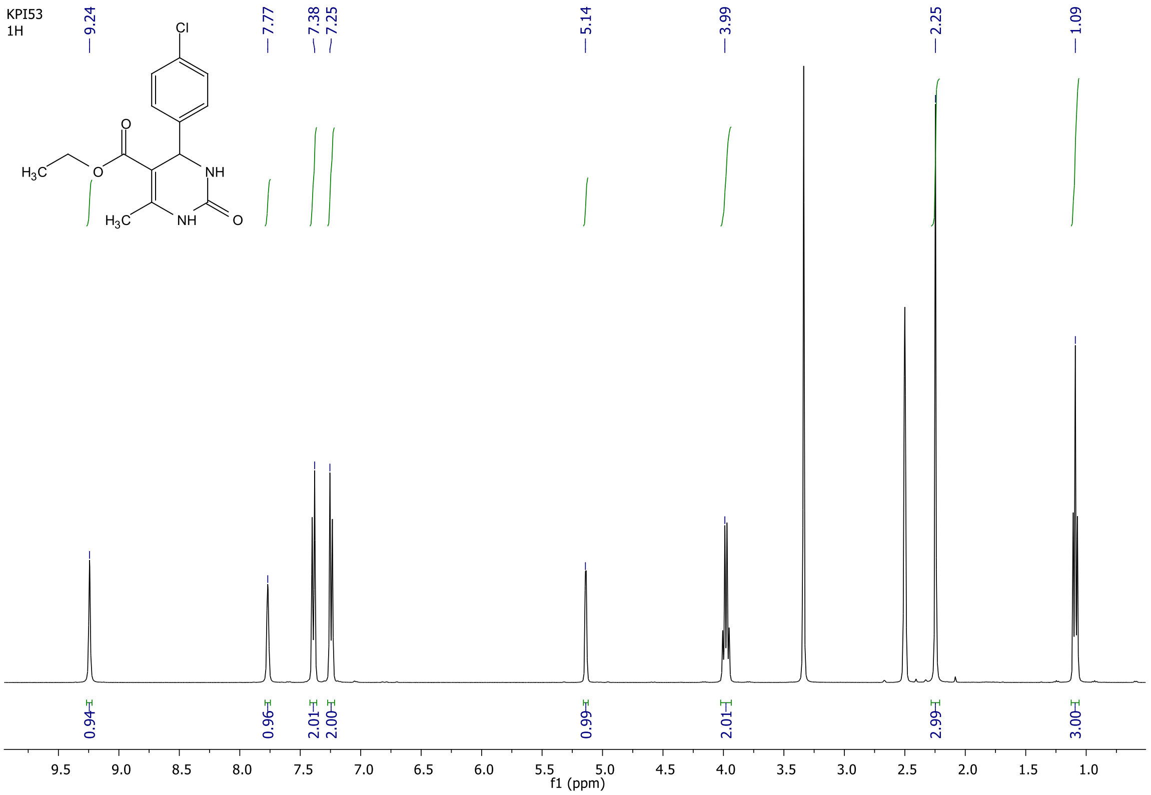

Figure 11 ^{1}H NMR of **4** in DMSO-d6

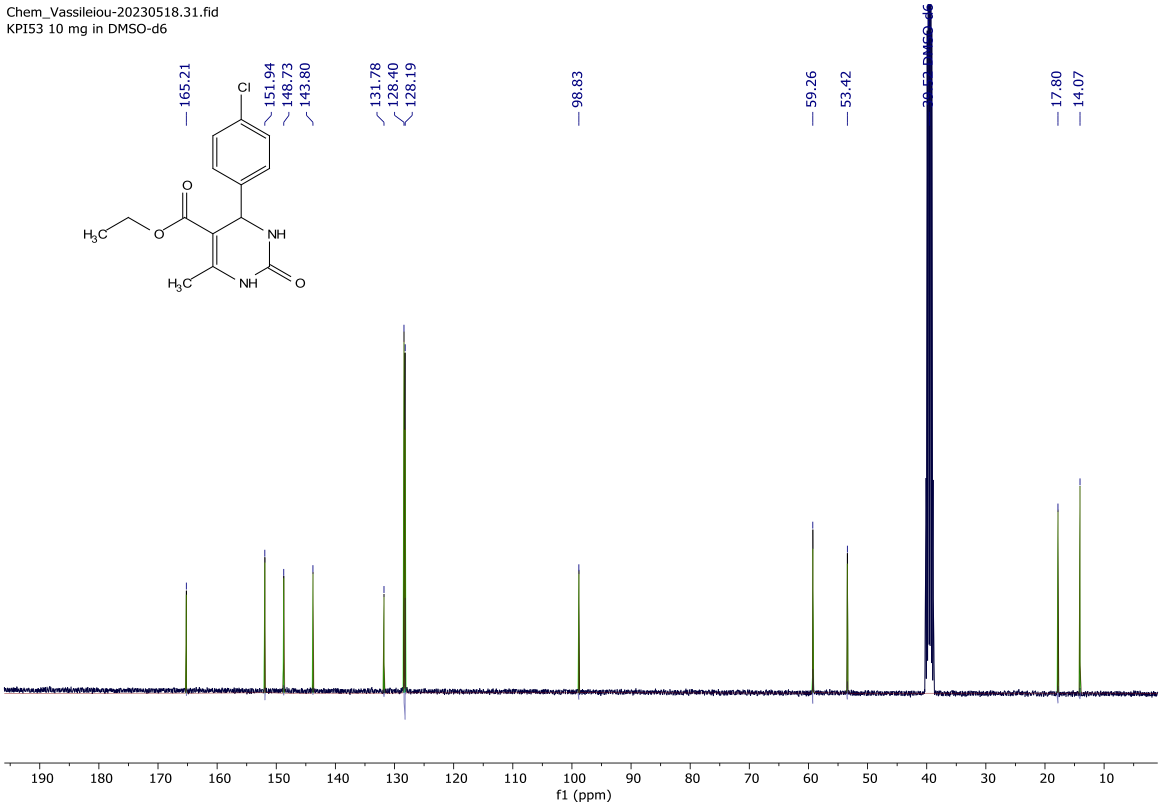

Figure 12 ^{13}C NMR of **4** in DMSO-d6

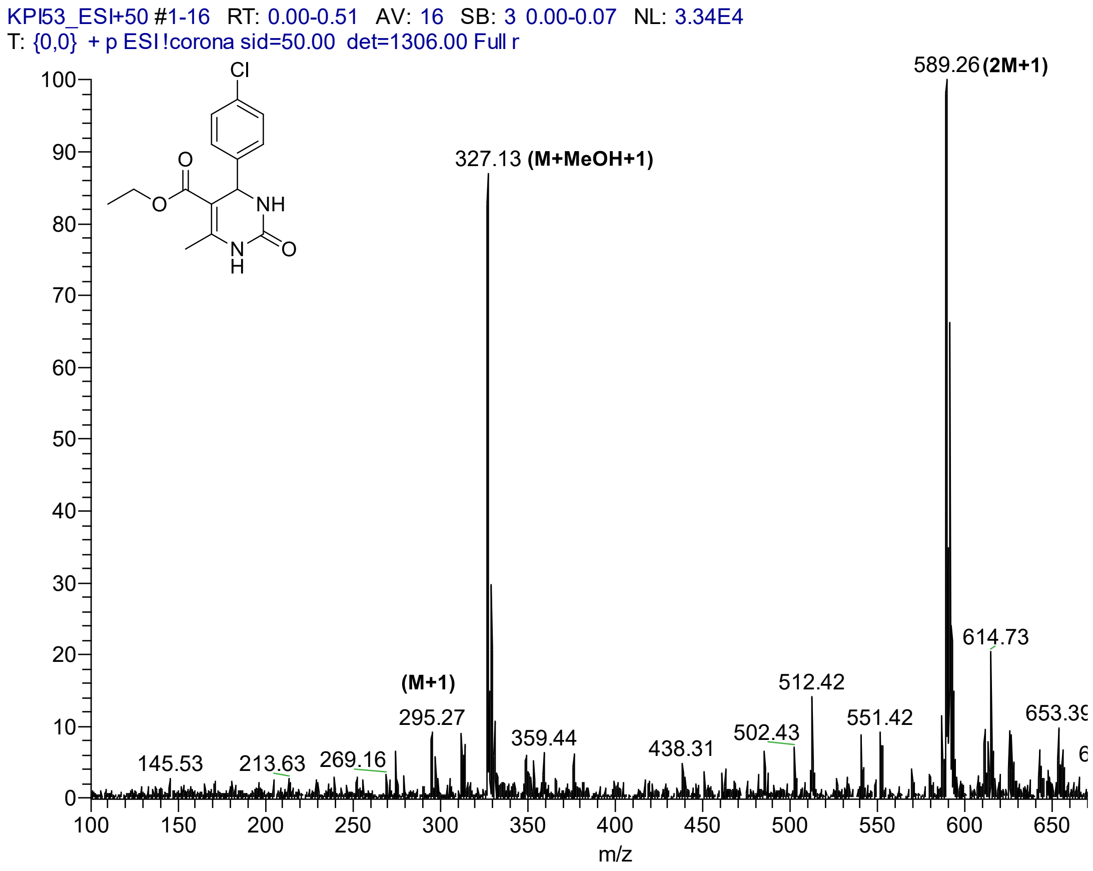

Figure 13 ESI-MS of **4**

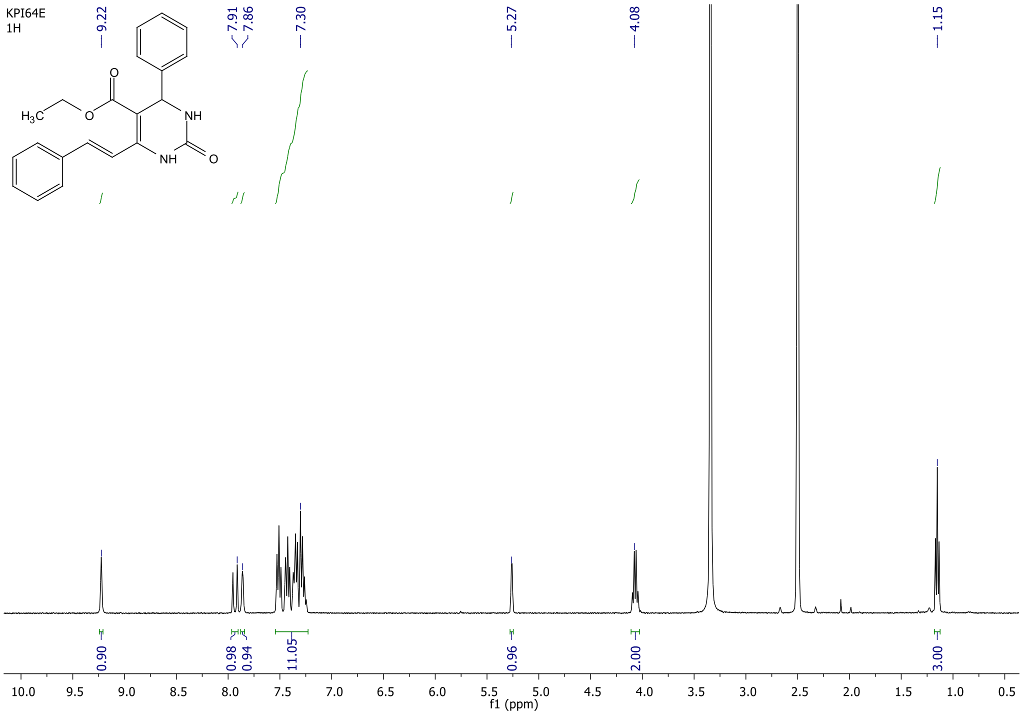

Figure 14 ^{1}H NMR of **10** in DMSO-d6

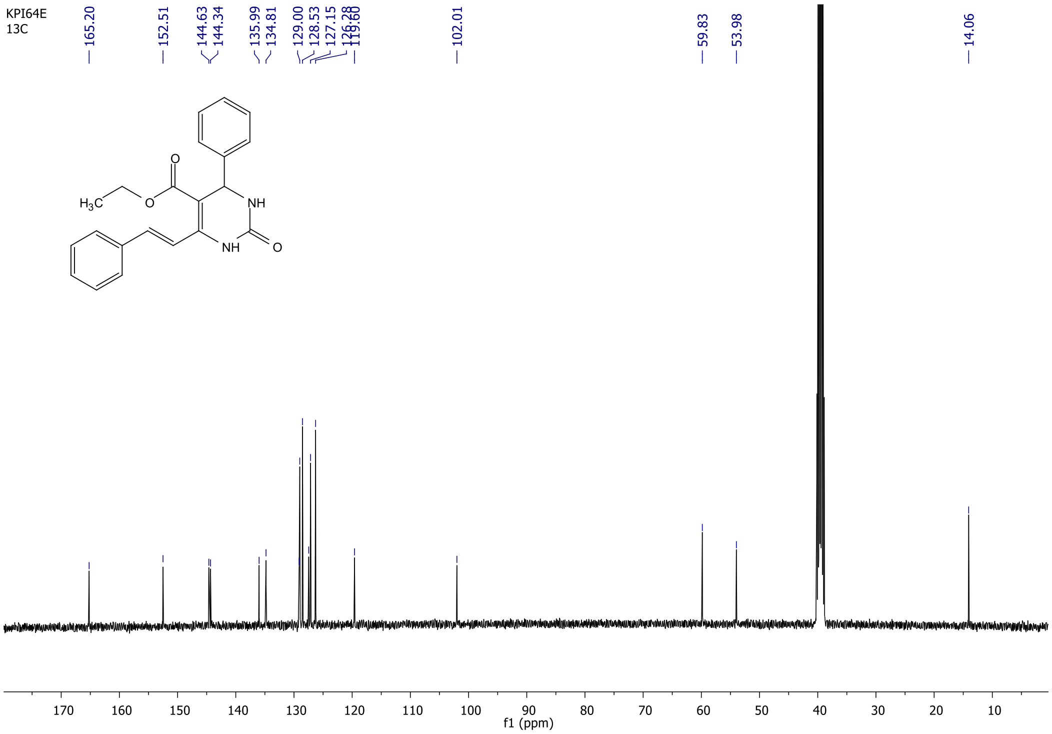

Figure 15 ^{13}C NMR of **10** in DMSO-d6

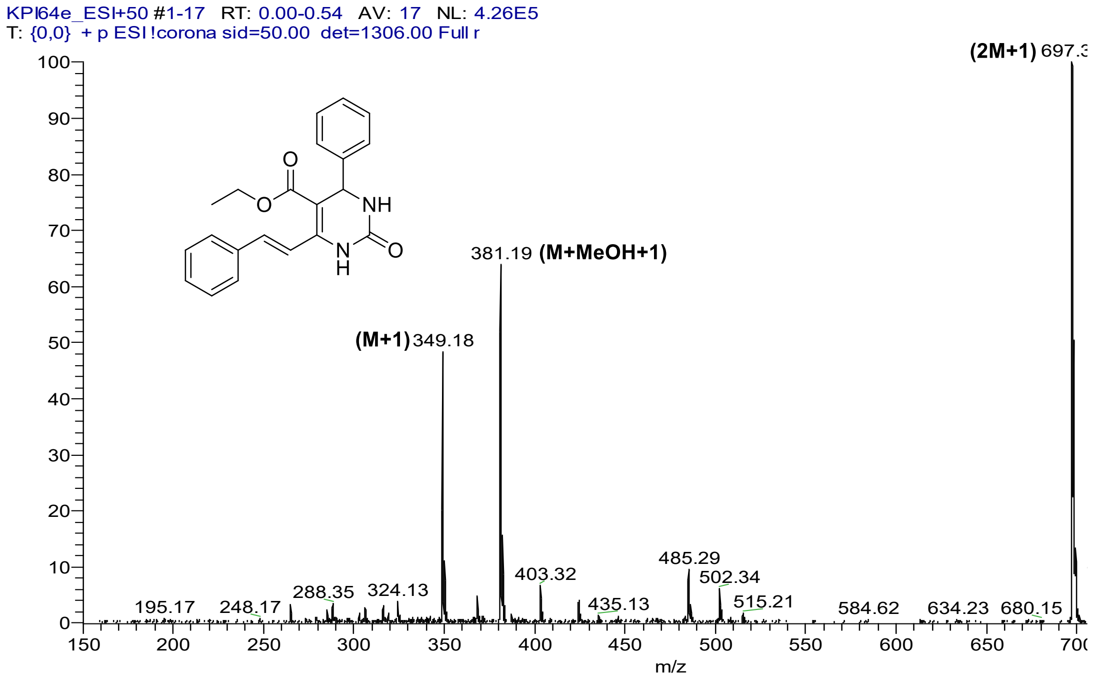

Figure 16 ESI-MS of **10**

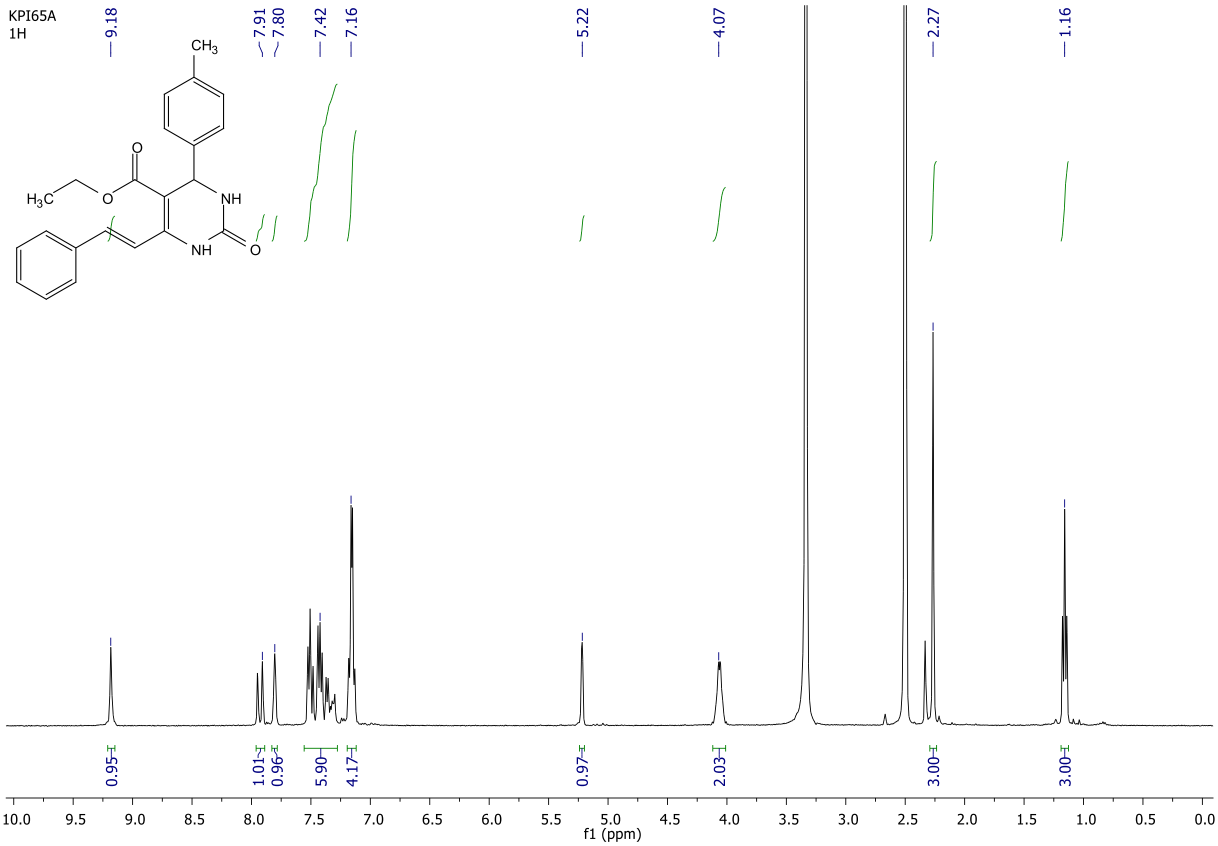

Figure 17 ^{1}H NMR of **11** in DMSO-d6

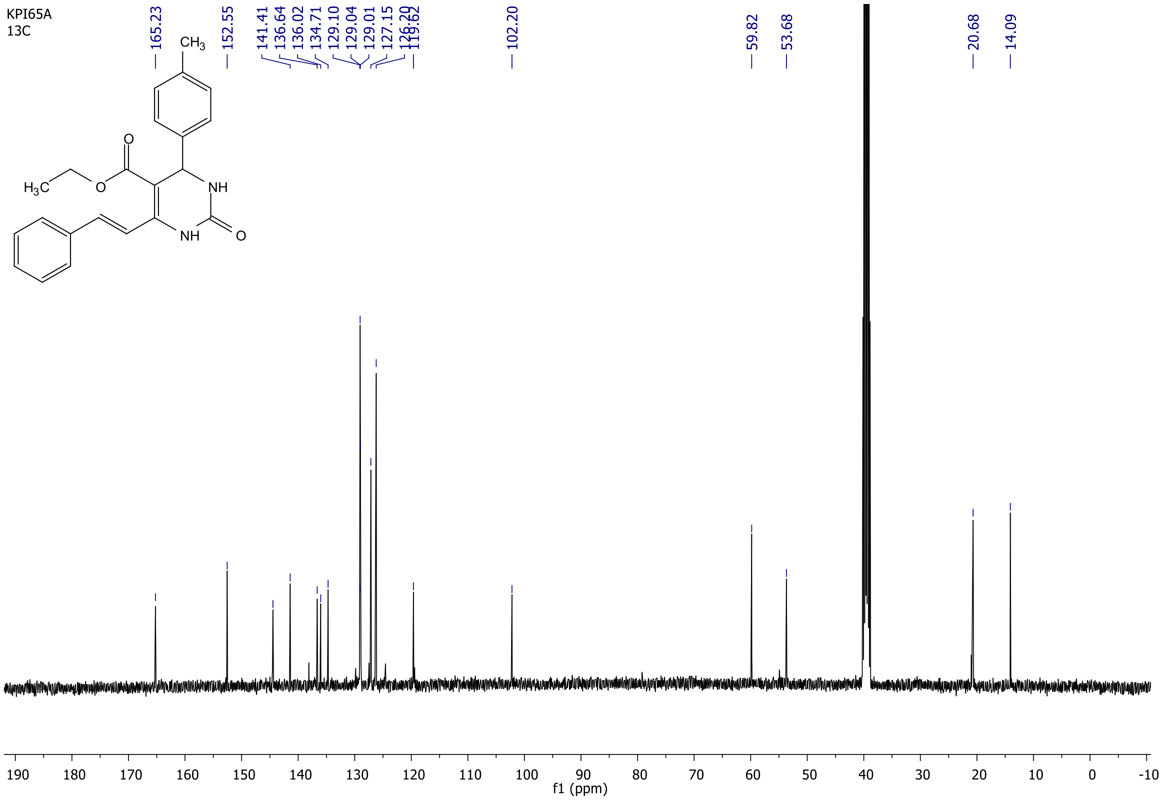

Figure 18 ^{13}C NMR of **11** in DMSO-d6

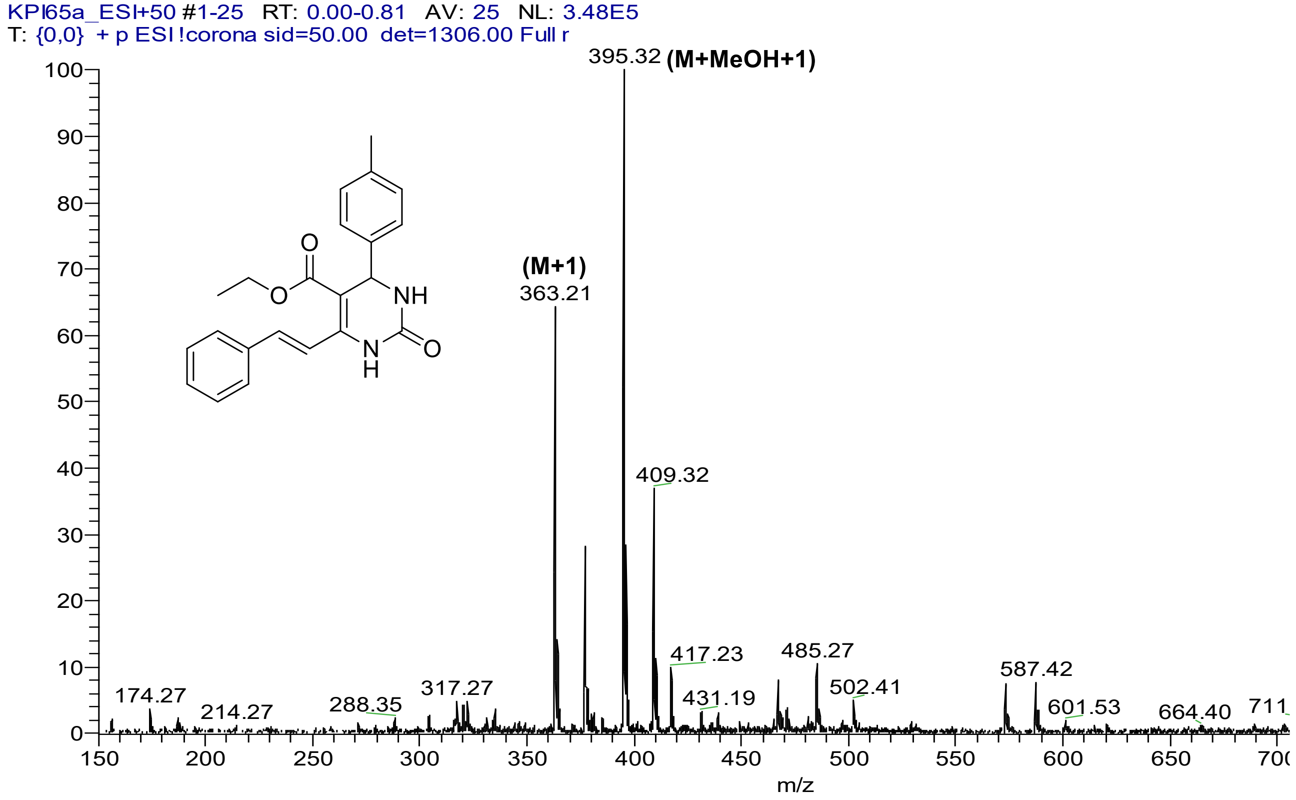

Figure 19 ESI-MS of **11**

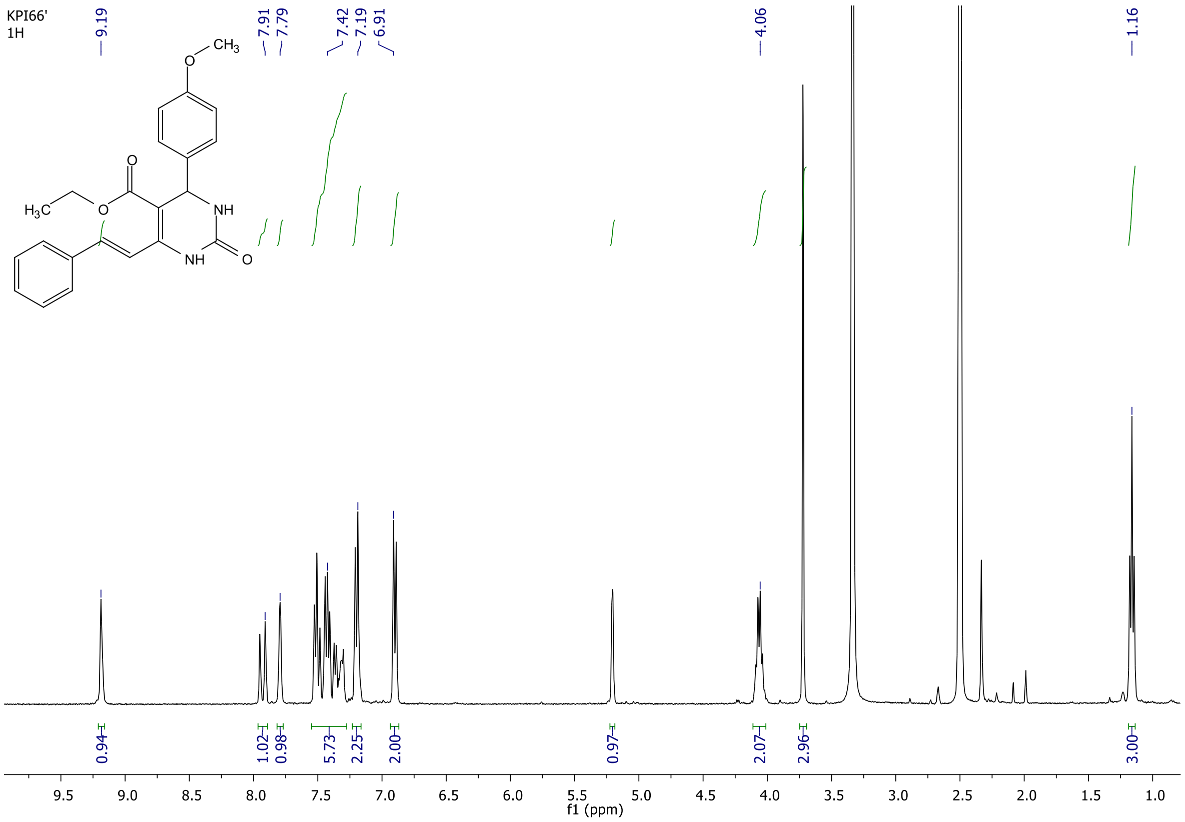

Figure 20 ^{1}H NMR of **12** in DMSO-d6

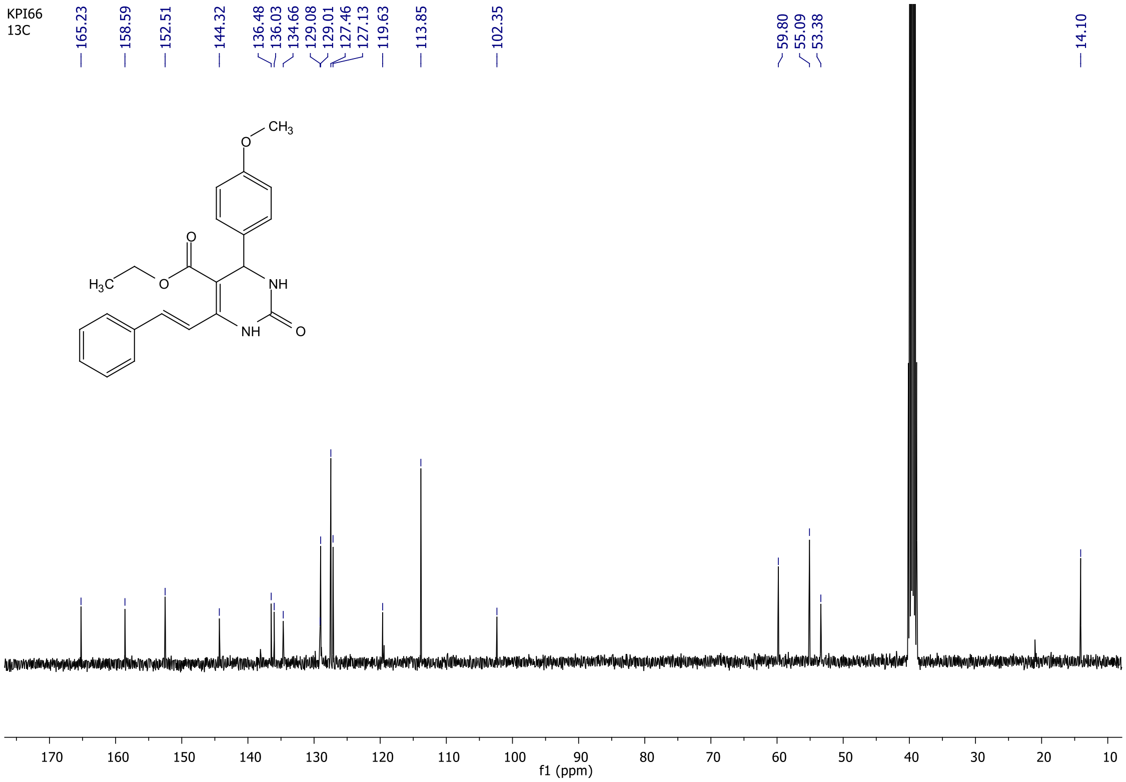

Figure 21 ^{13}C NMR of **12** in DMSO-d6

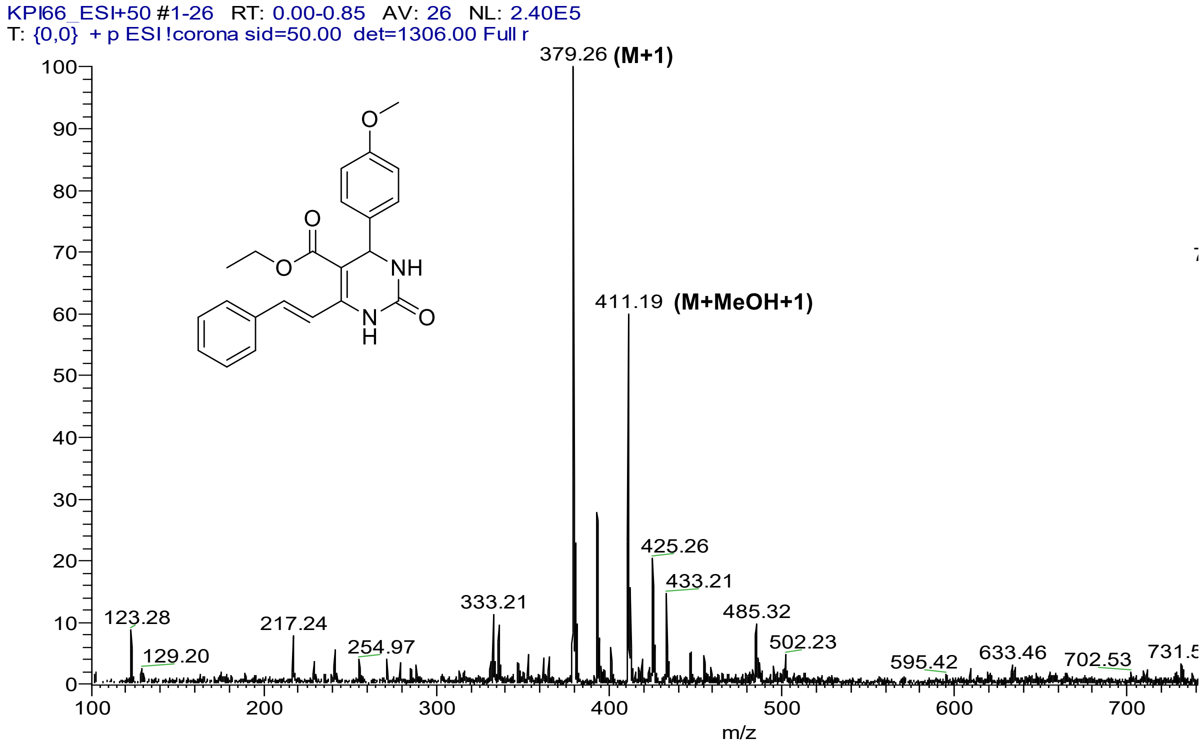

Figure 22 ESI-MS of **12**
