# Supplementary material for: Development of Styryl‐Modified 3,4‐Dihydropyrimidin‐2(1H)‐ones as Potential Antitumor Agents
Source: ChemMedChem. 2026 Apr 7;21(7):e202501073. doi: 10.1002/cmdc.202501073 (PMC13056349; doi:10.1002/cmdc.202501073)
Supplement: Supplementary file 1 — Supplementary Material [file CMDC-21-e202501073-s001.zip › Supporting Information 2_3_02_2026.pdf]

SUPPORTING INFORMATION 2

Development of Styryl-modified 3,4-Dihydropyrimidin-2(1*H*)-ones as Potential Antitumor Agents

*Konstantinos Panagoulias,[a] Dr. Woonghee Kim,[b] Dr. Murat Ozdemir, [c] Dr. Busra Turan, [c] Prof. Adil Mardinoglu,[b, d] Prof. Hasan Turkez,[e] Daniela Trisciuzzi, [f] Orazio Nicolotti, [f] Prof. Antonio Di Stefano, [g] Prof. Stamatia Vassiliou**[a] and Prof. Ivana Cacciatore,* [g]*

[a] K. Panagoulias, Prof. Stamatia Vassiliou**
Laboratory of Organic Chemistry, Department of Chemistry
National and Kapodistrian University of Athens
Athens, GREECE
E-mail: kostas.panagoulias@protonmail.com, svassiliou@chem.uoa.gr

[b] Dr. Woonghee Kim, Prof. Adil Mardinoglu
Science for Life Laboratory
KTH-Royal Institute of Technology,
SE-17121 Stockholm, SWEDEN
E-mail: woonghee.kim@scilifelab.se, adilm@scilifelab.se

[c] Dr. Murat Ozdemir, Dr. Busra Turan
Trustlife Laboratories
Drug Research & Development Center
34774, Istanbul, TURKEY
E-mail: murat.ozdemir@trustlifelabs.com, busra.turan@trustlifelabs.com

[d] Prof. Adil Mardinoglu
Centre for Host-Microbiome Interactions
Faculty of Dentistry, Oral & Craniofacial Sciences
King's College London
London SE1 9RT, UNITED KINGDOM
E-mail: adilm@scilifelab.se

[e] Prof. Hasan Turkez
Department of Medical Biology, Faculty of Medicine
Atatürk University
Erzurum, TURKEY
E-mail: hasan.turkez@gmail.com

[f] Prof. Daniela Trisciuzzi, Prof. Orazio Nicolotti
Department of Pharmacy, Pharmaceutical Sciences,

University of Bari "Aldo Moro"
Bari, ITALY
E-mail: daniela.trisciuzzi@uniba.it, orazio.nicolotti@uniba.it

[g] Prof. Antonio Di Stefano, Prof. Ivana Cacciatore*
Department of Pharmacy
"G. D'Annunzio" University of Chieti-Pescara
66100, Chieti Scalo (Chieti), ITALY
E-mail: antonio.distefano@unich.it, ivana.cacciatore@unich.it

NMR and MS spectra of 13-21

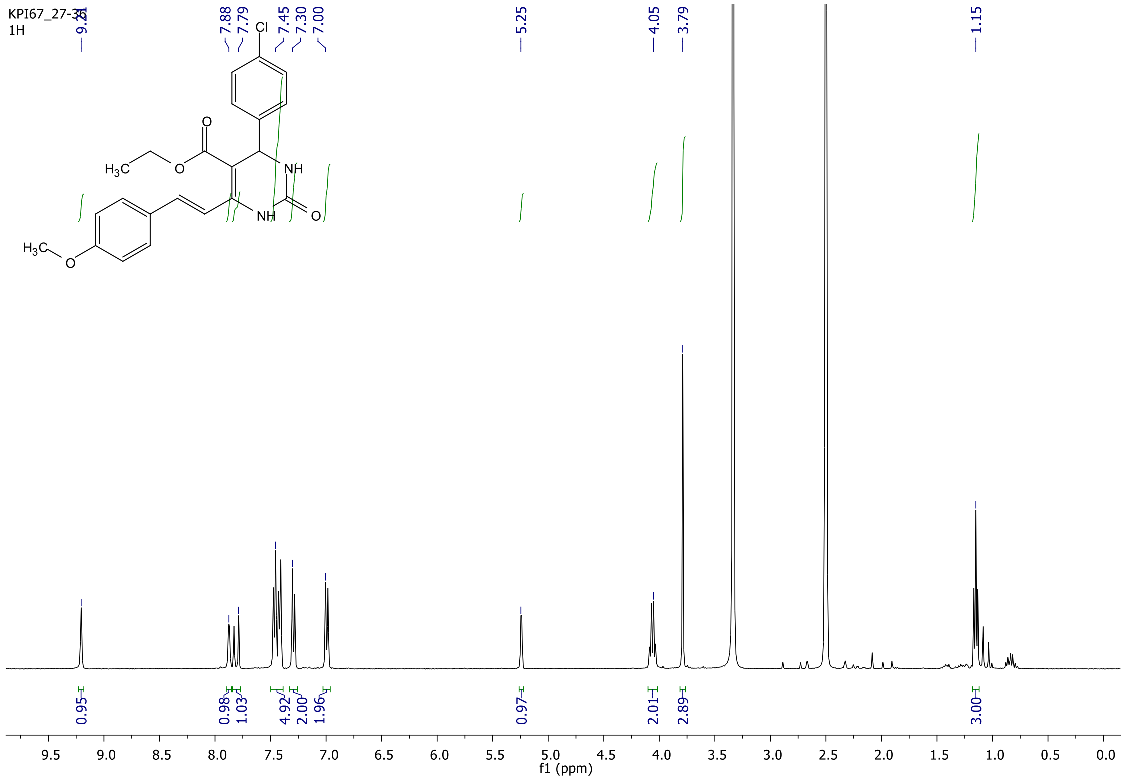

Figure 23 ^{1}H NMR of **13** in DMSO-d6

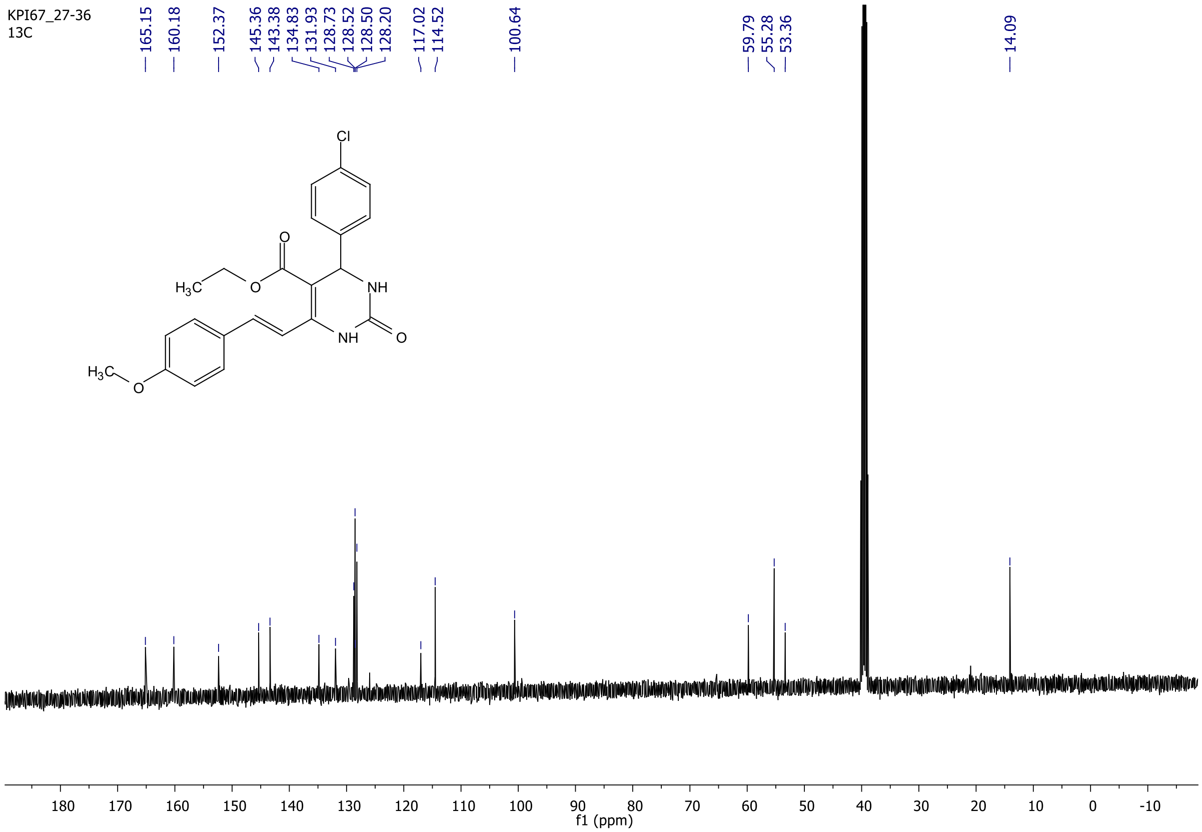

Figure 24 ^{13}C NMR of **13** in DMSO-d6

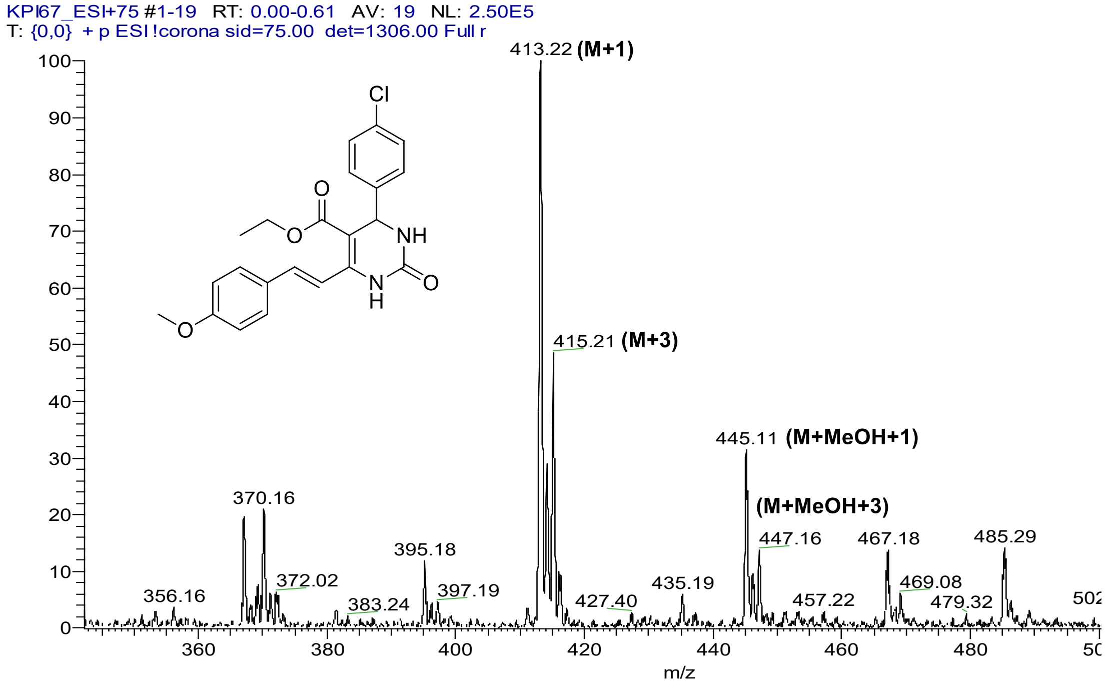

Figure 25 ESI-MS of **13**

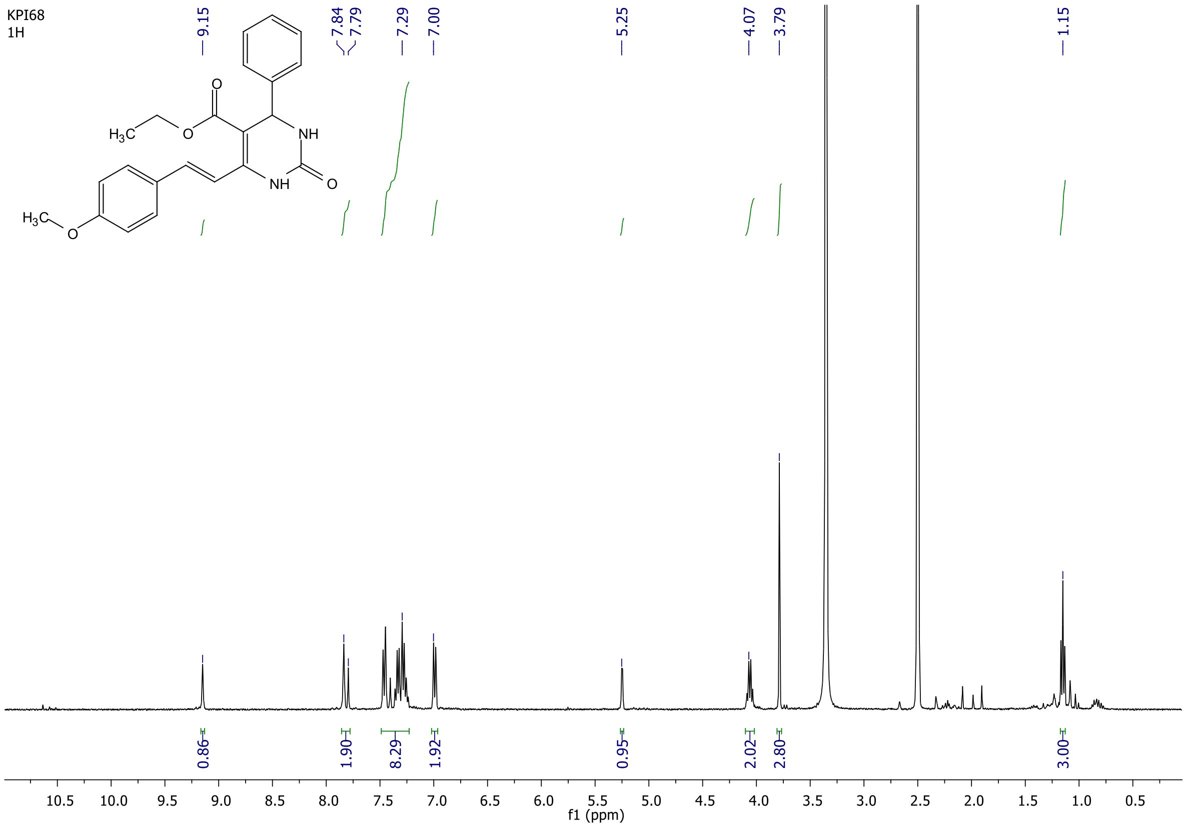

Figure 26 ^{1}H NMR of **14** in DMSO-d6

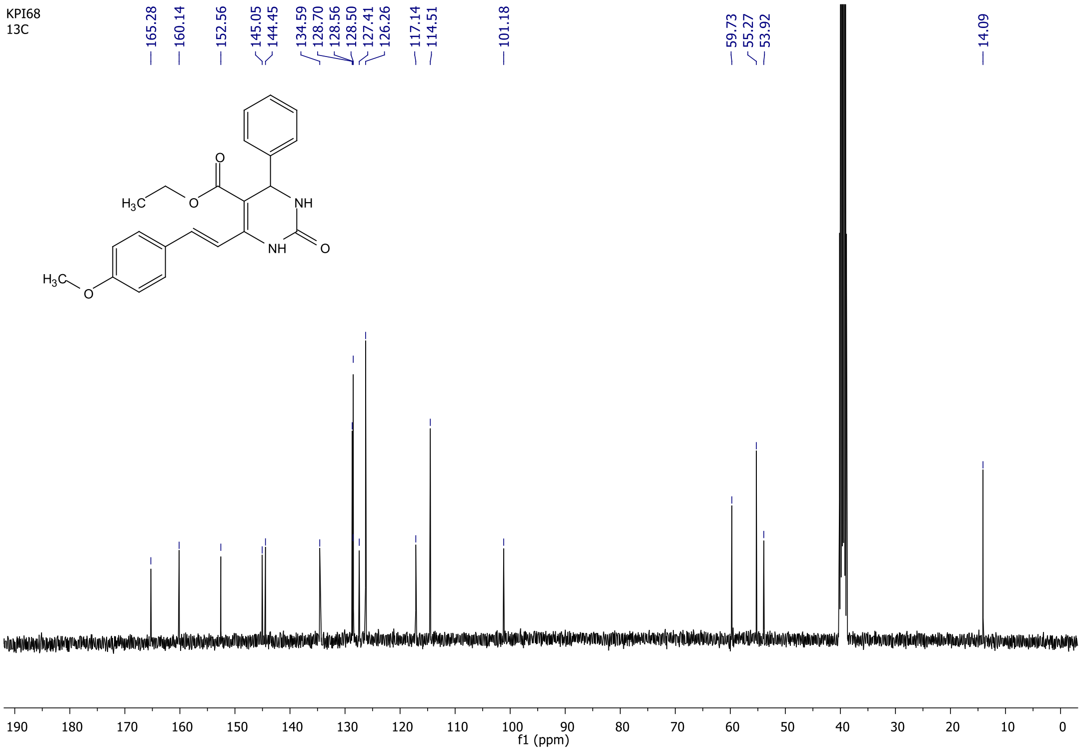

Figure 27 ^{13}C NMR of **14** in DMSO-d6

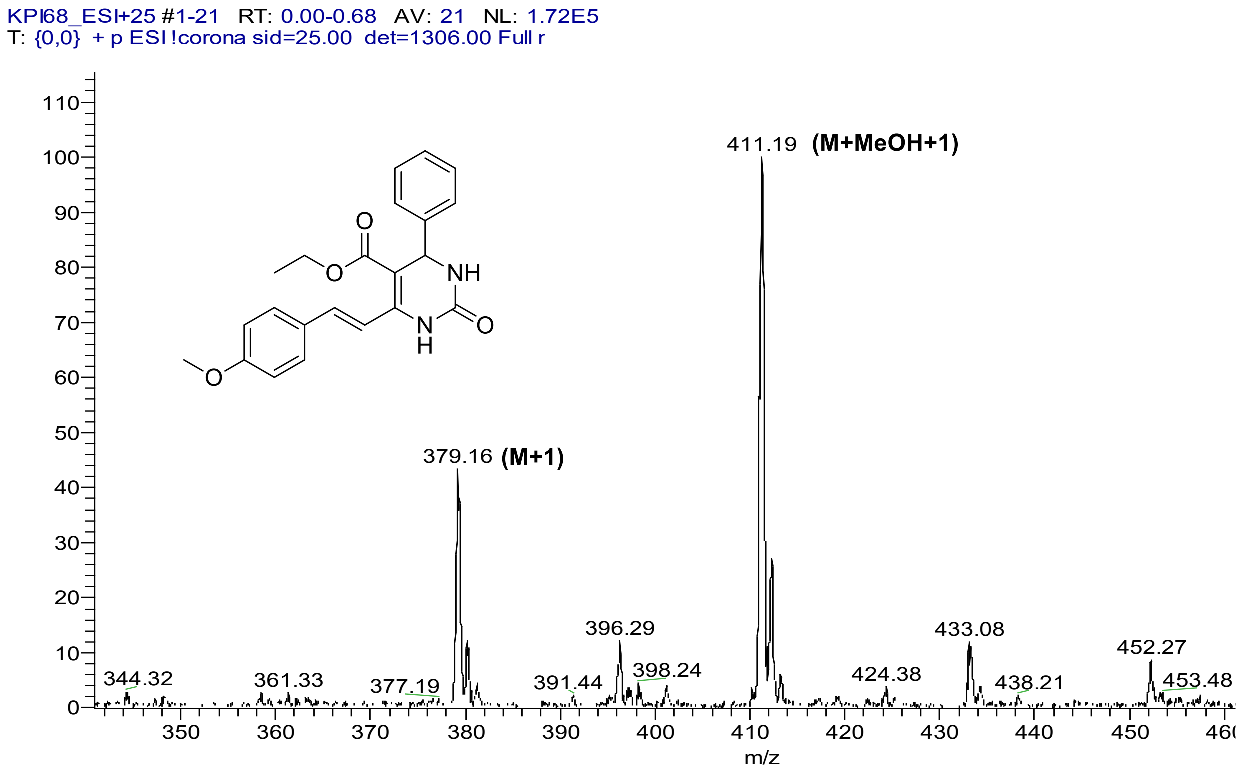

Figure 28 ESI-MS of **14**

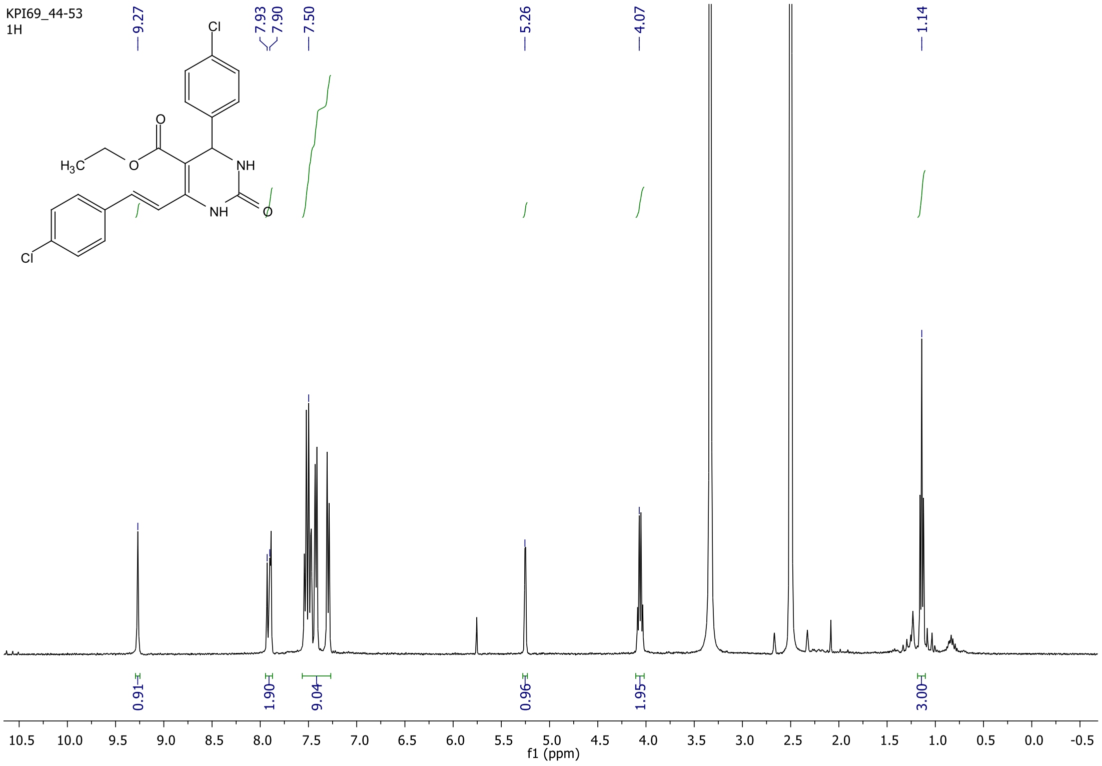

Figure 29 ^{1}H NMR of **15** in DMSO-d6

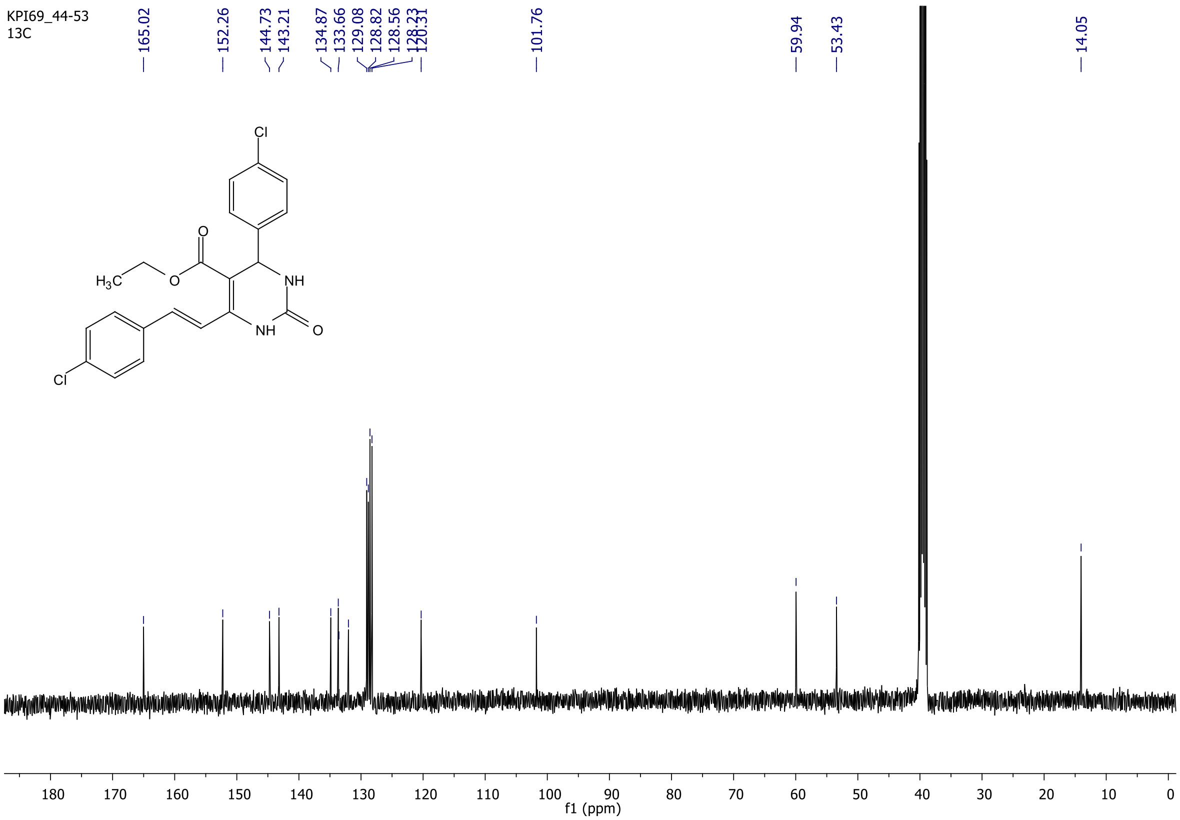

Figure 30 ^{13}C NMR of **15** in DMSO-d6

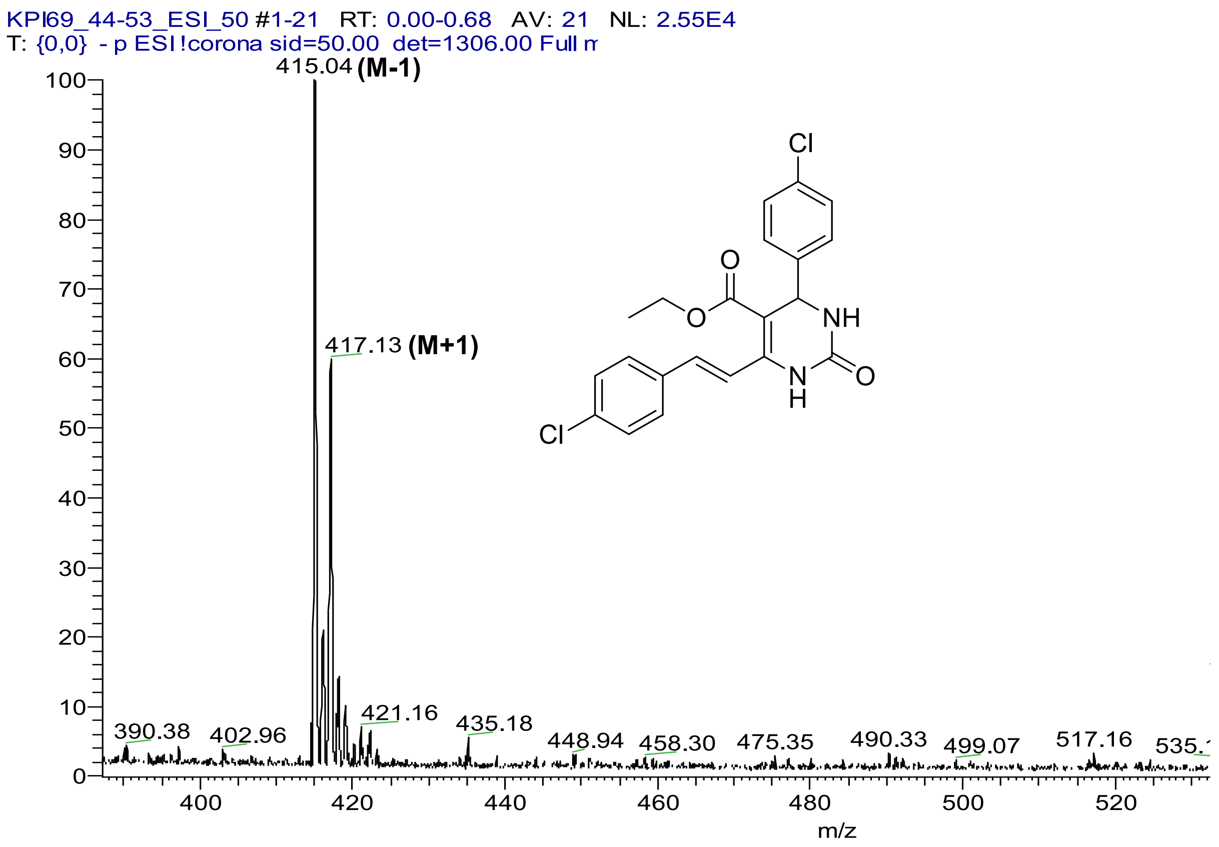

Figure 31 ESI-MS of **15**

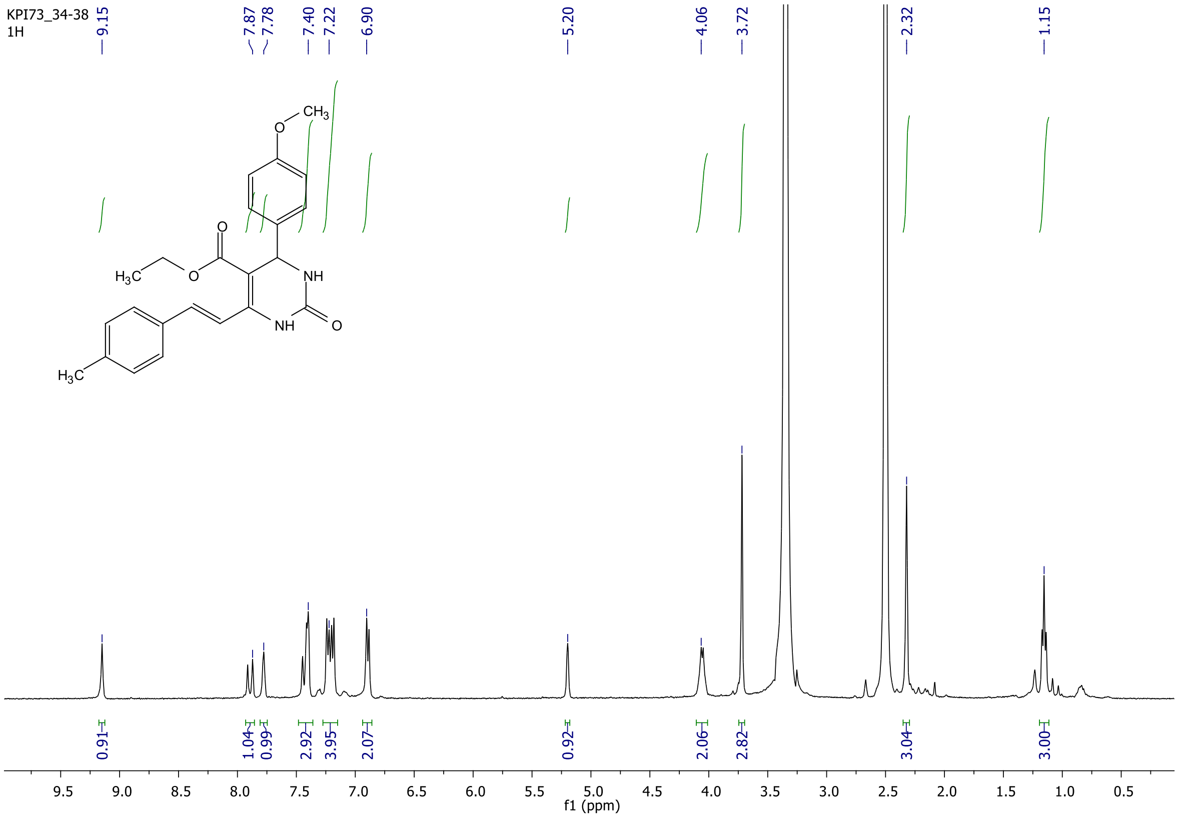

Figure 32 ^{1}H NMR of **16** in DMSO-d6

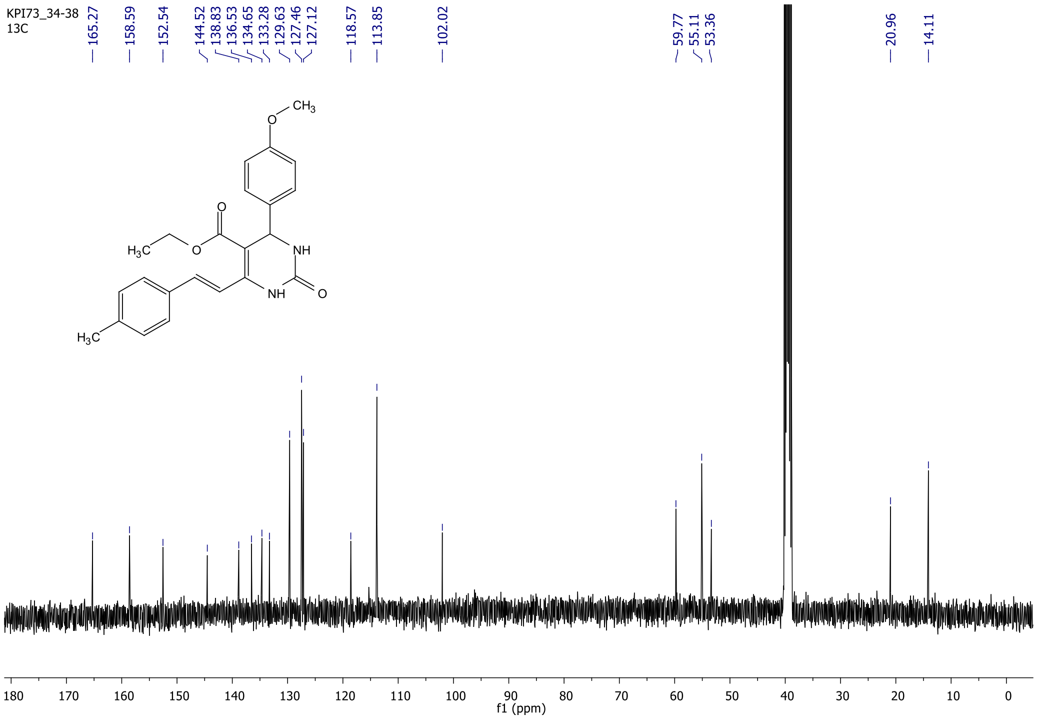

Figure 33 ^{13}C NMR of **16** in DMSO-d6

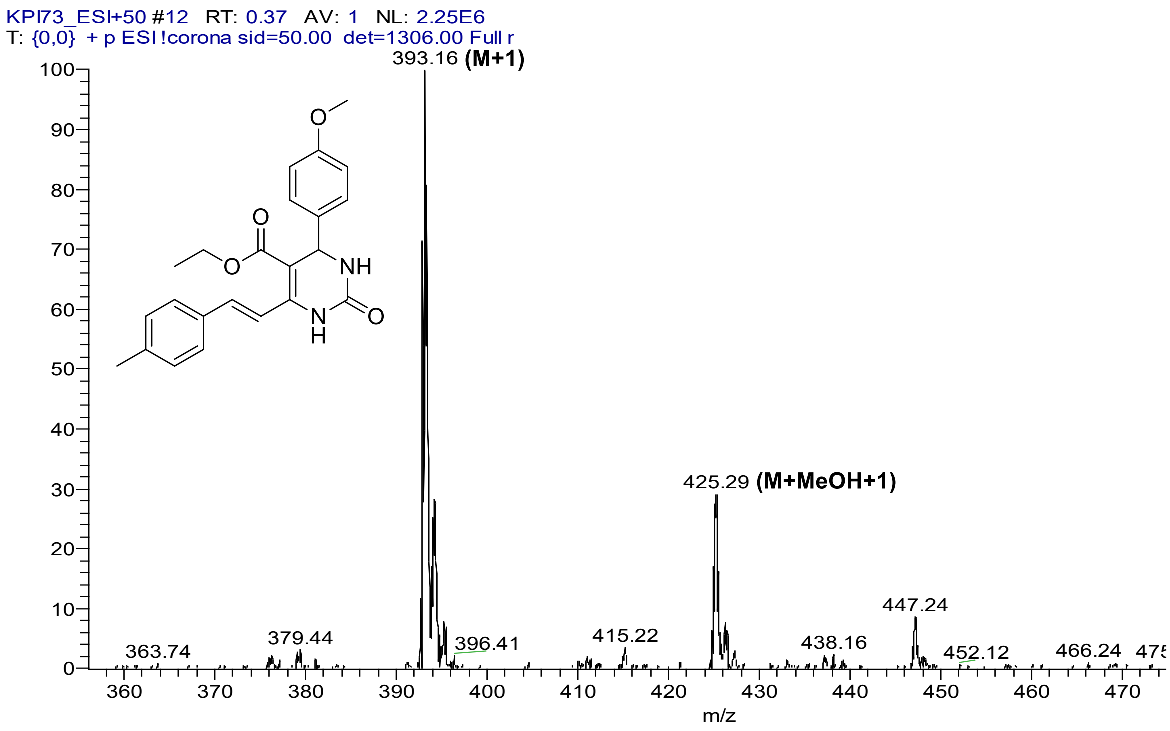

Figure 34 ESI-MS of **16**

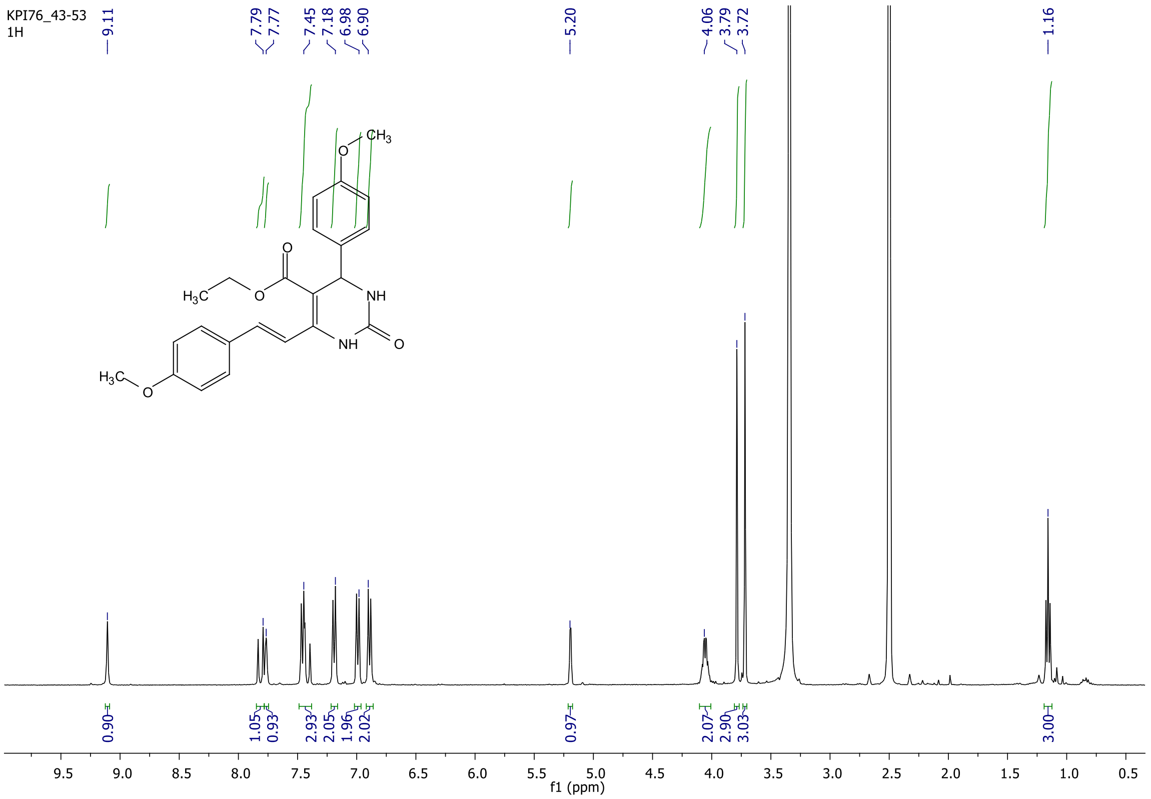

Figure 35 ^{1}H NMR of **17** in DMSO-d6

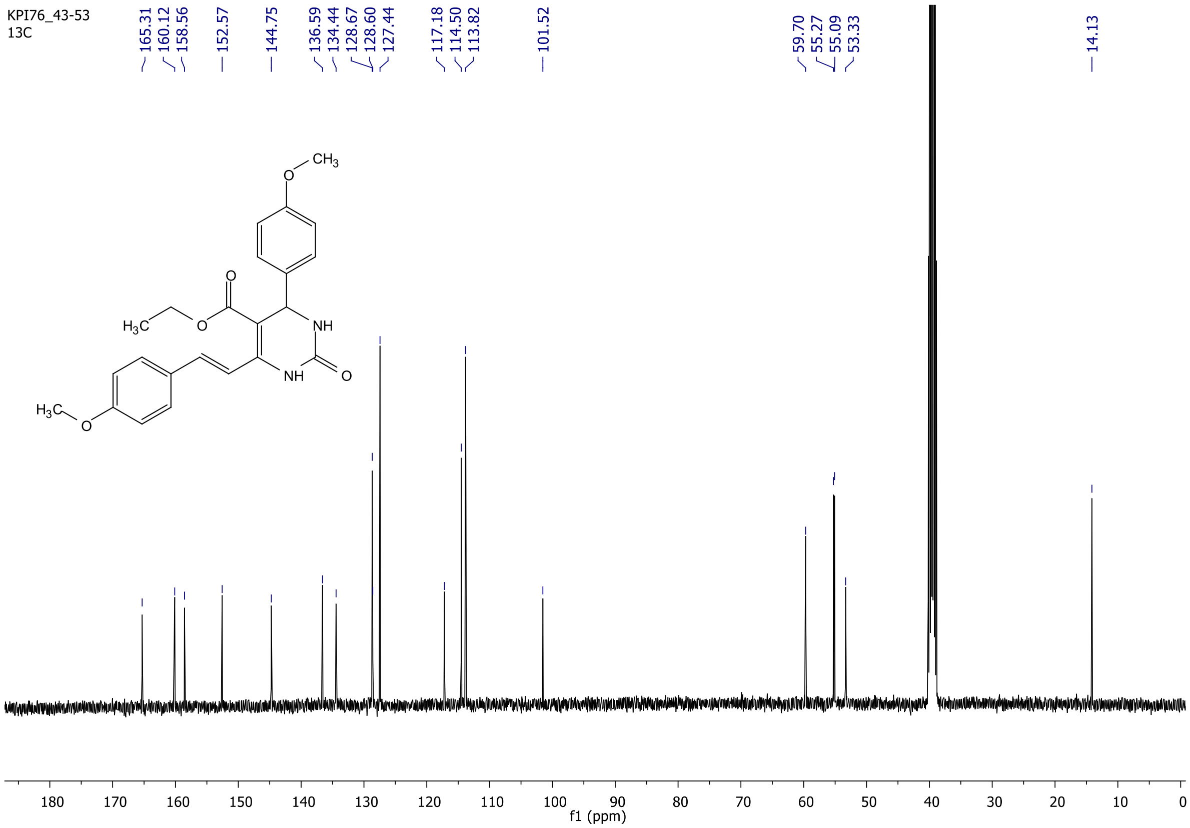

Figure 36 ^{13}C NMR of **17** in DMSO-d6

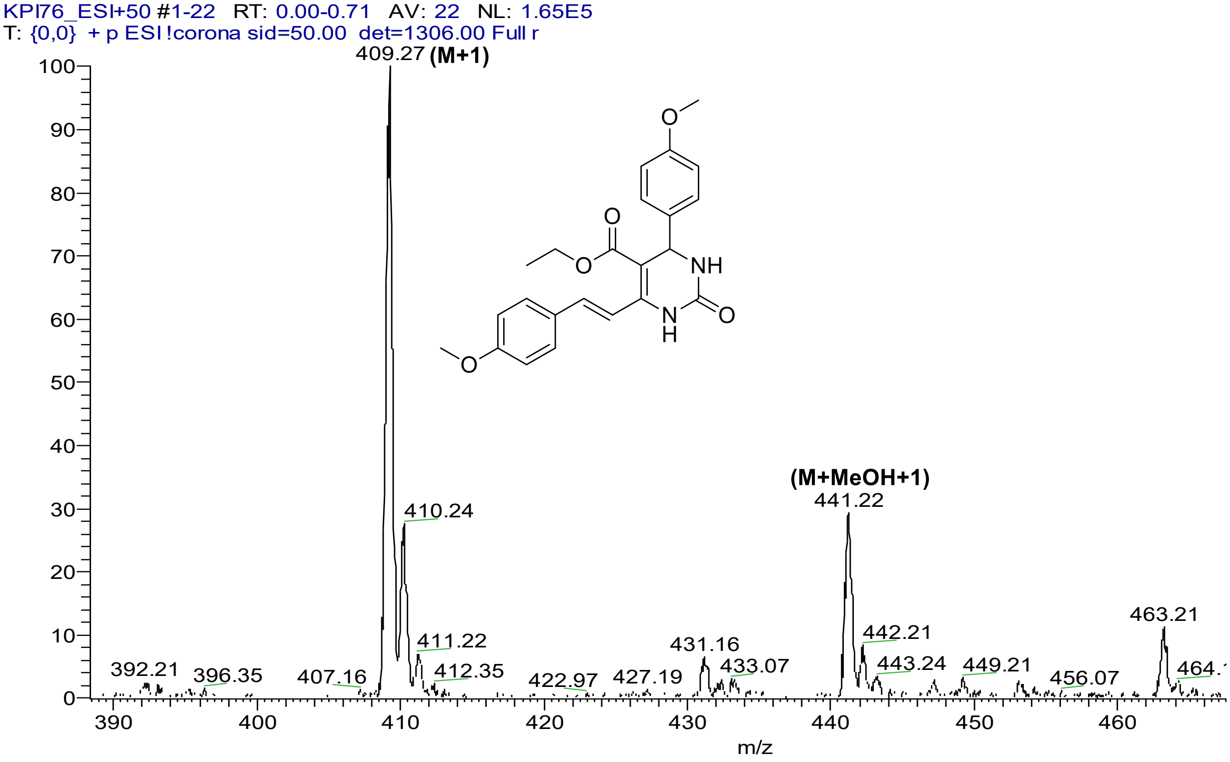

Figure 37 ESI-MS of **17**

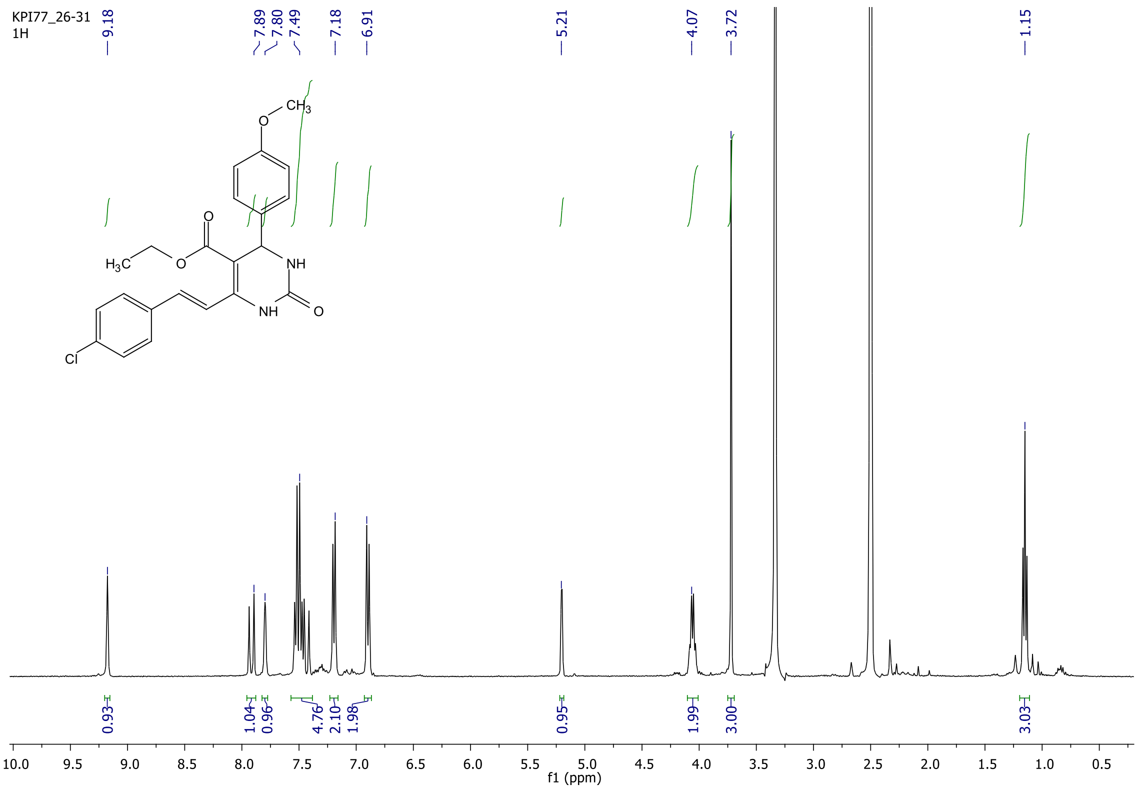

Figure 38 ^{1}H NMR of **18** in DMSO-d6

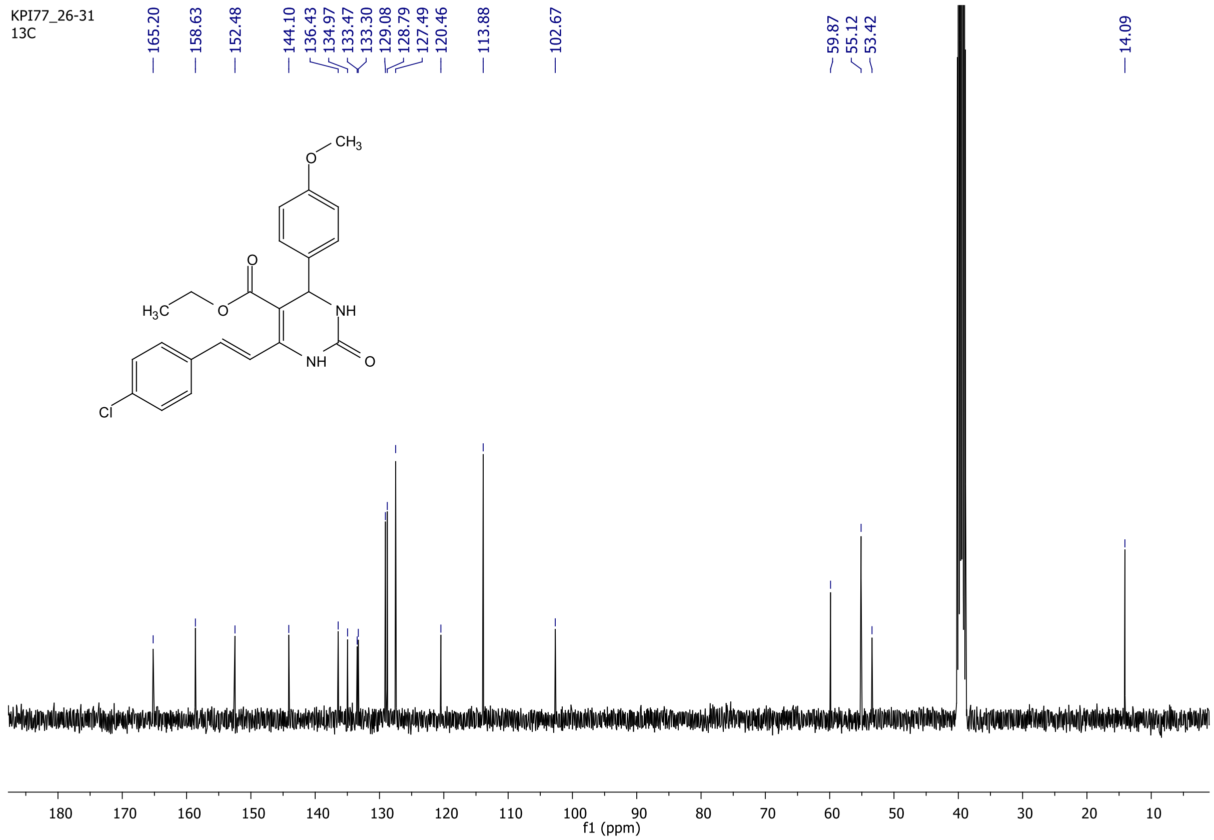

Figure 39 ^{13}C NMR of **18** in DMSO-d6

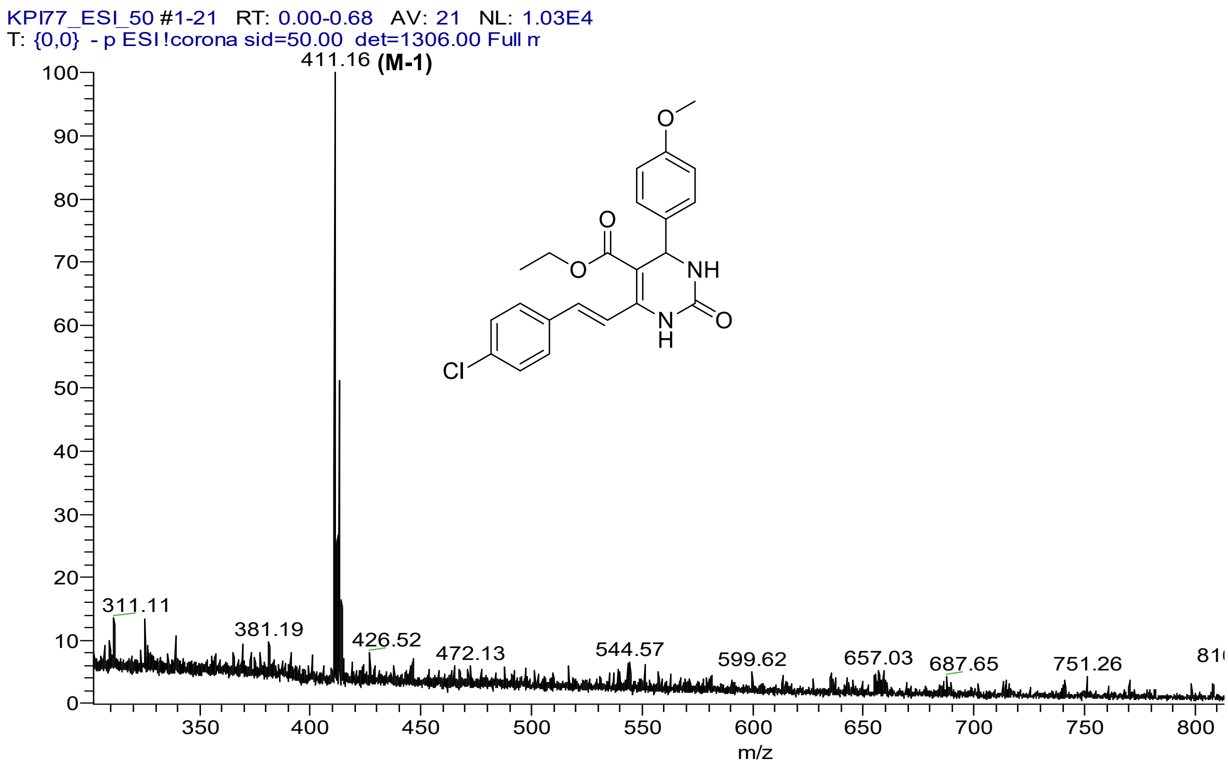

Figure 40 ESI-MS of **18**

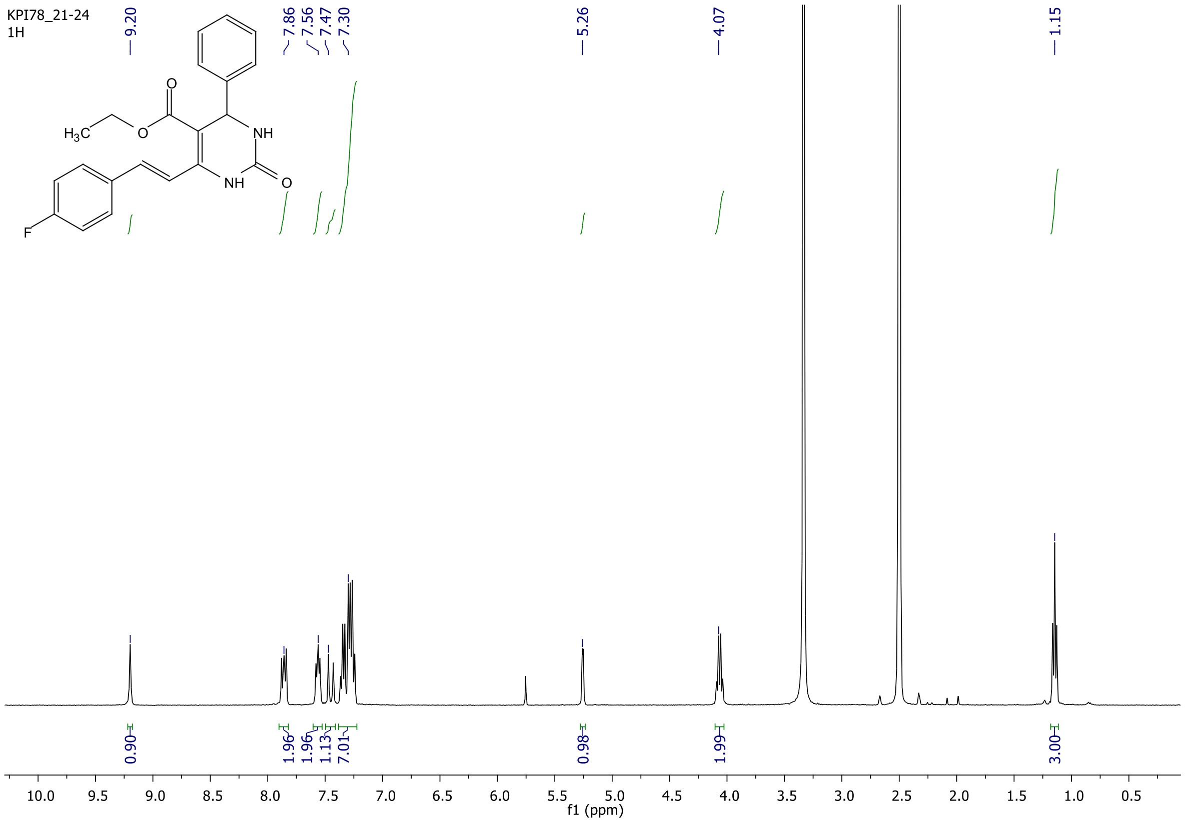

Figure 41 ^{1}H NMR of **19** in DMSO-d6

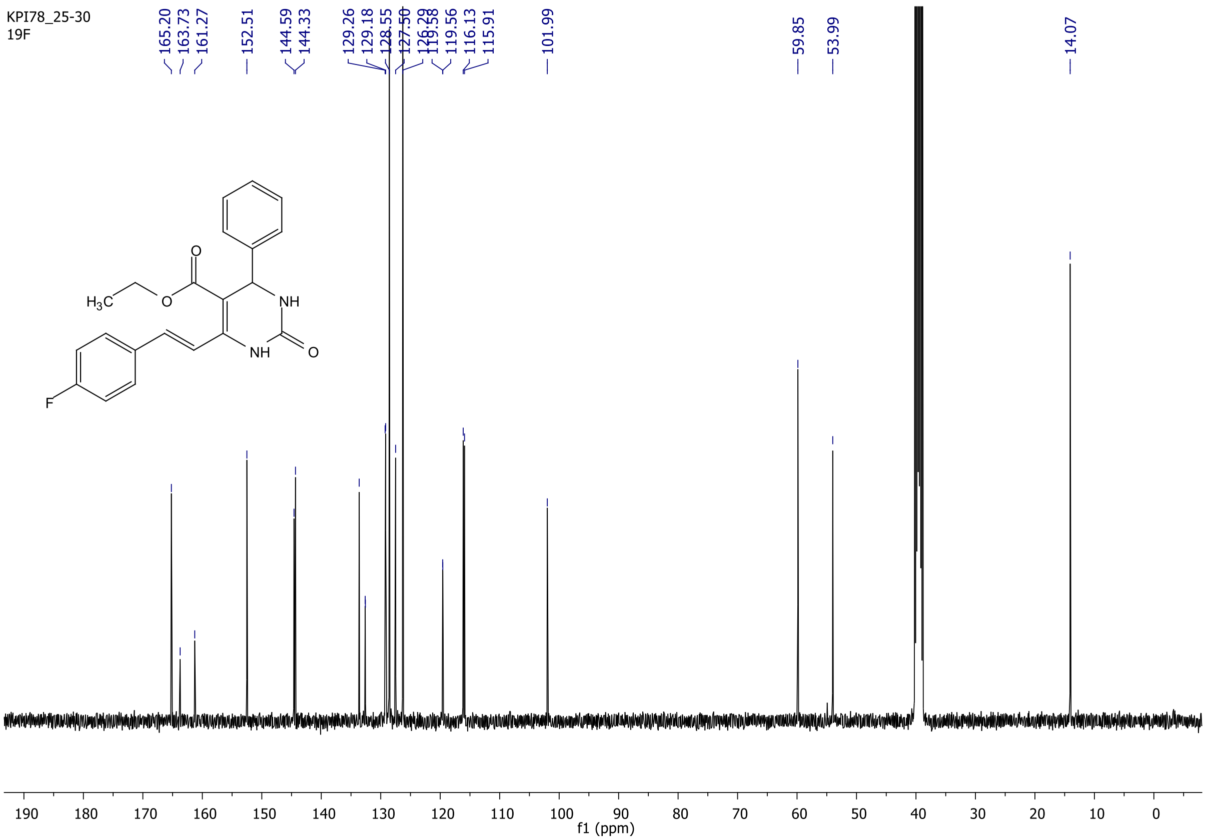

Figure 42 ^{13}C NMR of **19** in DMSO-d6

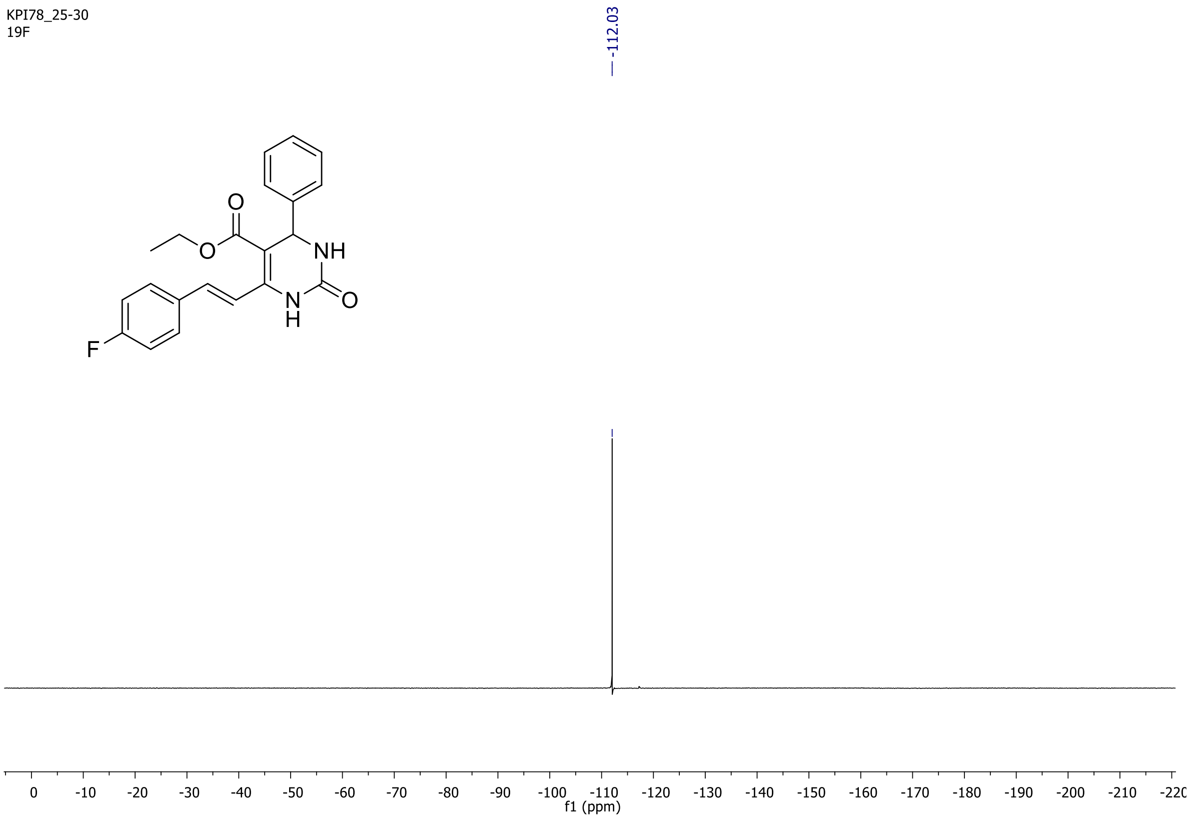

Figure 43 ^{19}F NMR of **19** in DMSO-d6

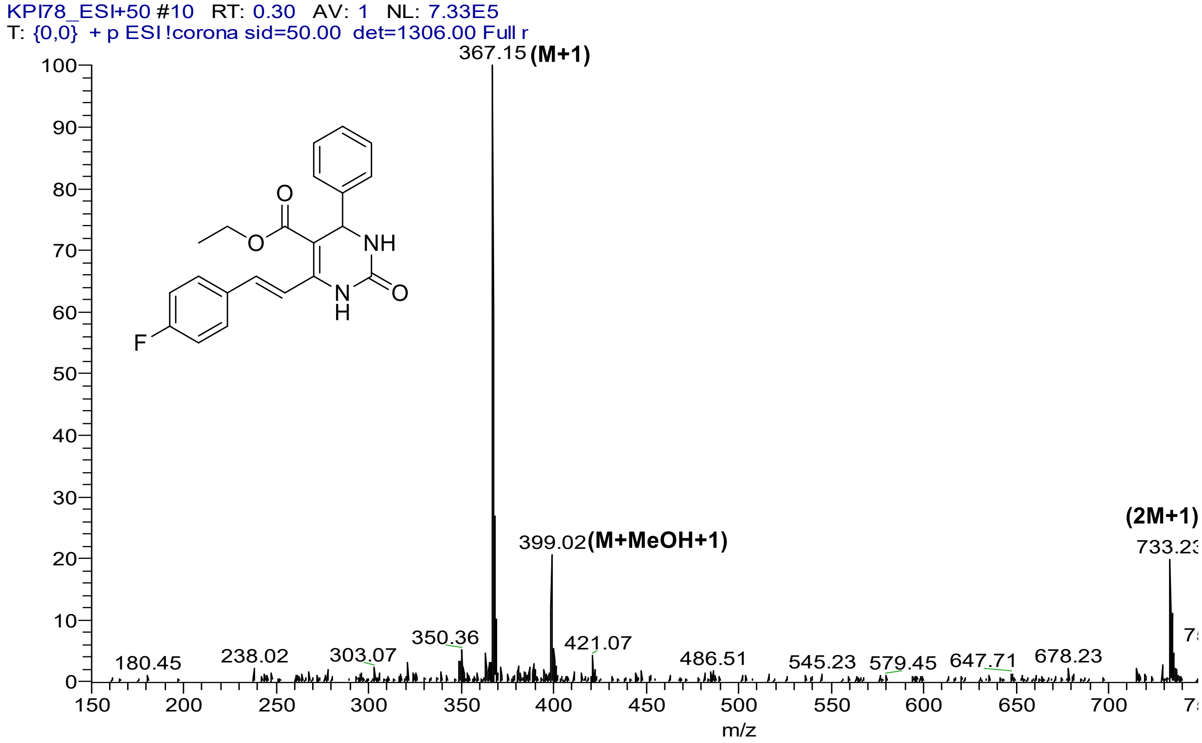

Figure 44 ESI-MS of **19**

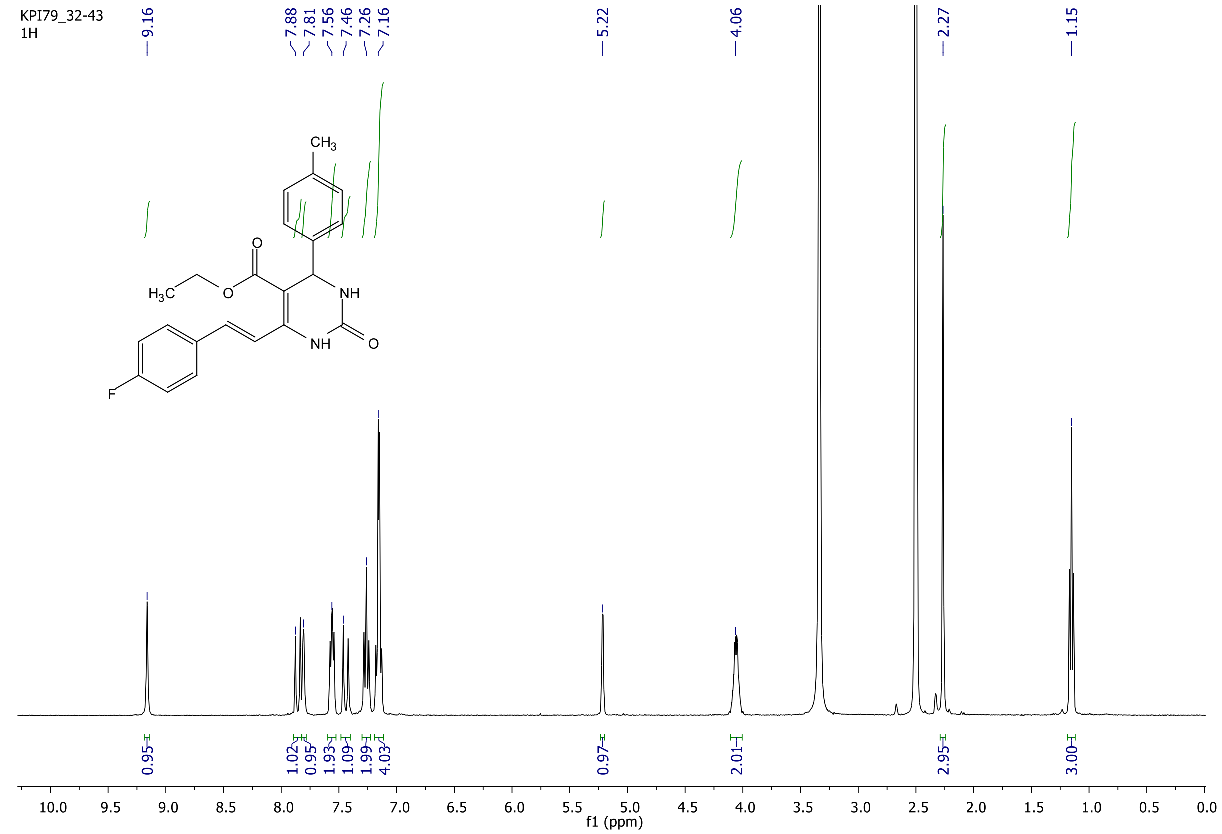

Figure 45 ^{1}H NMR of **20** in DMSO-d6

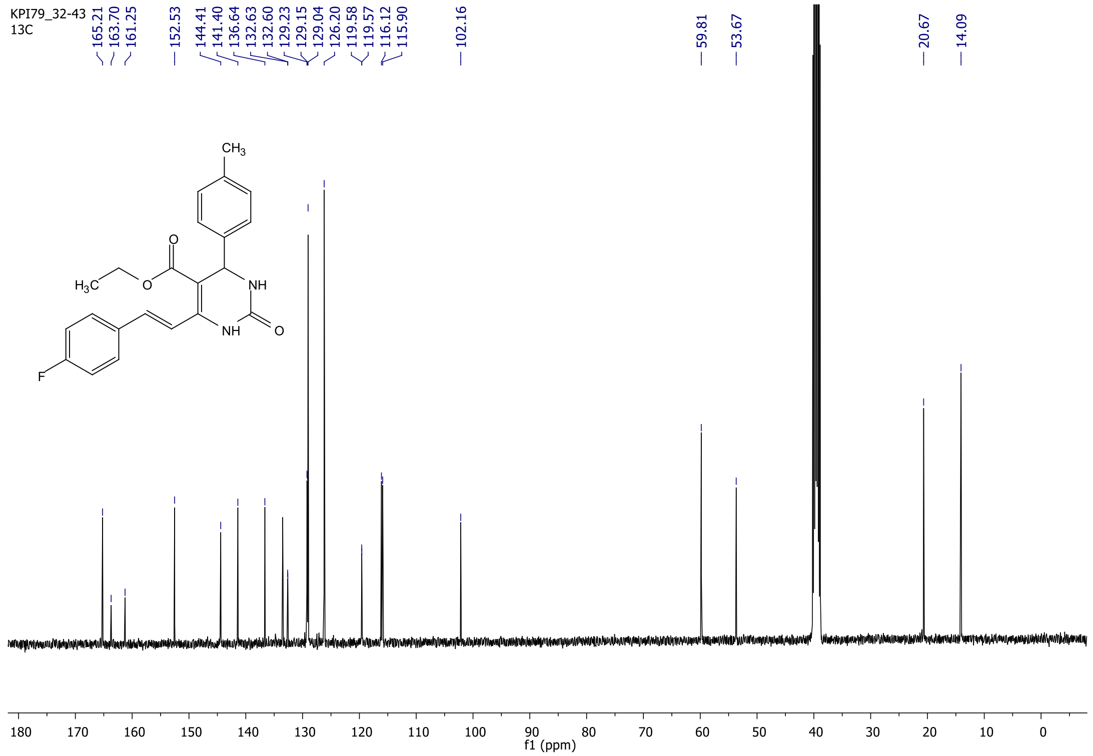

Figure 46 ^{13}C NMR of **20** in DMSO-d6

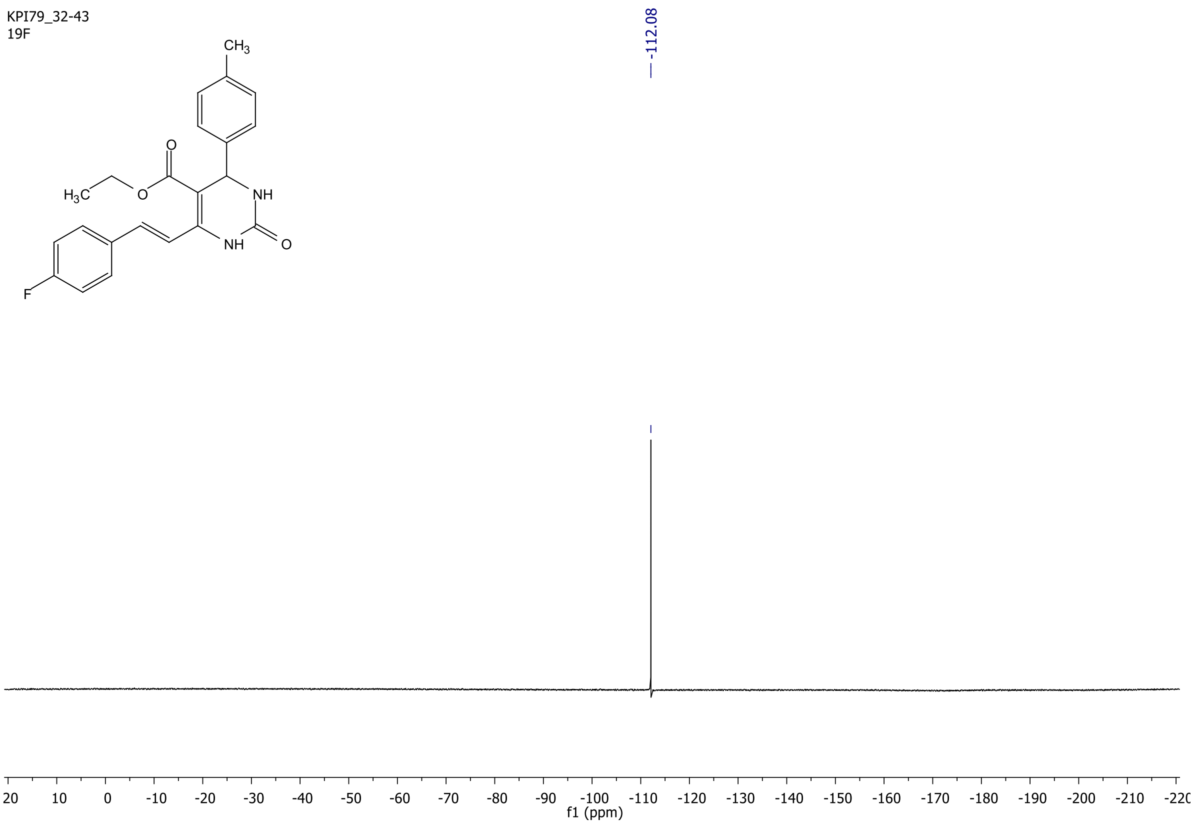

Figure 47 ^{19}F NMR of **20** in DMSO-d6

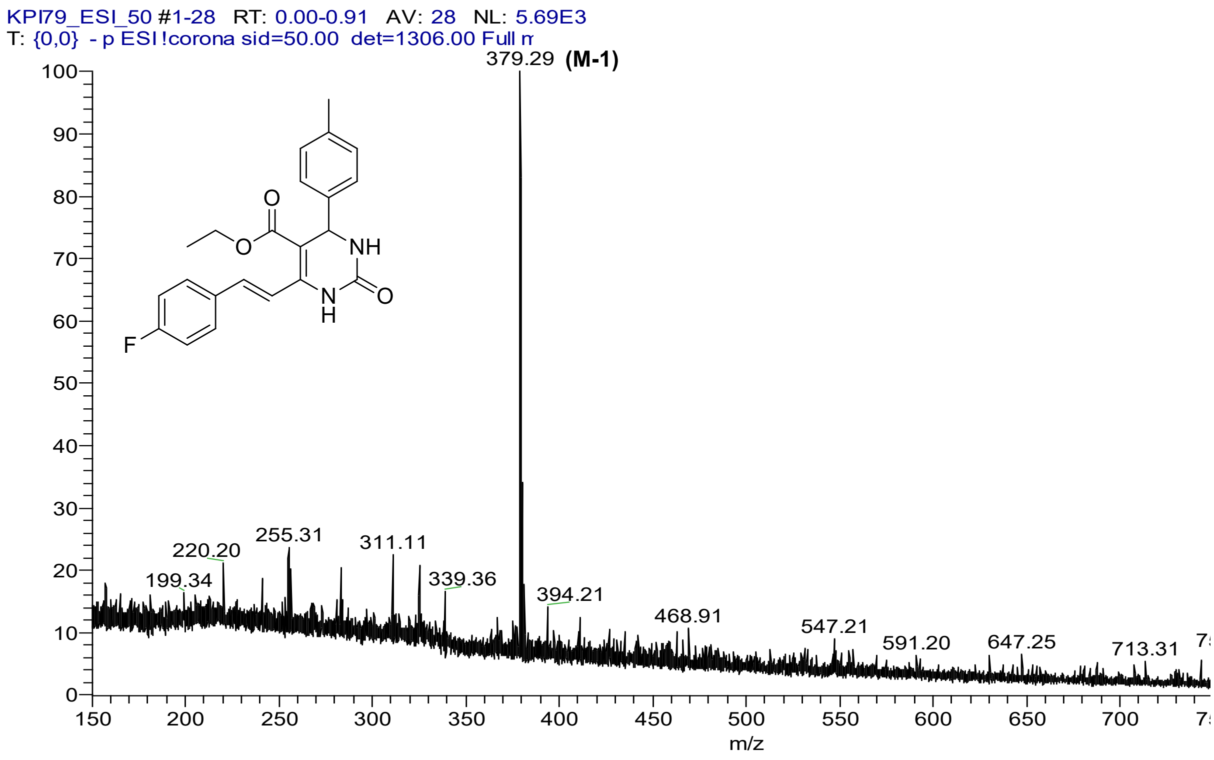

Figure 48 ESI-MS of **20**

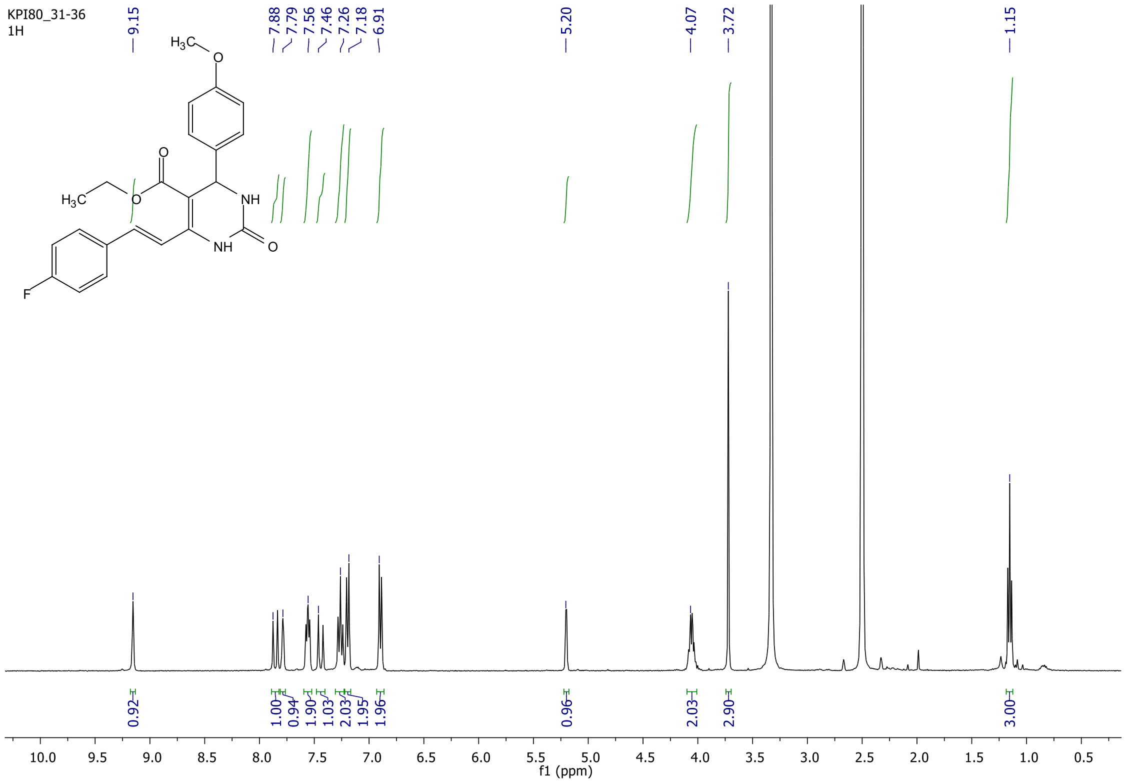

Figure 49 ^{1}H NMR of **21** in DMSO-d6

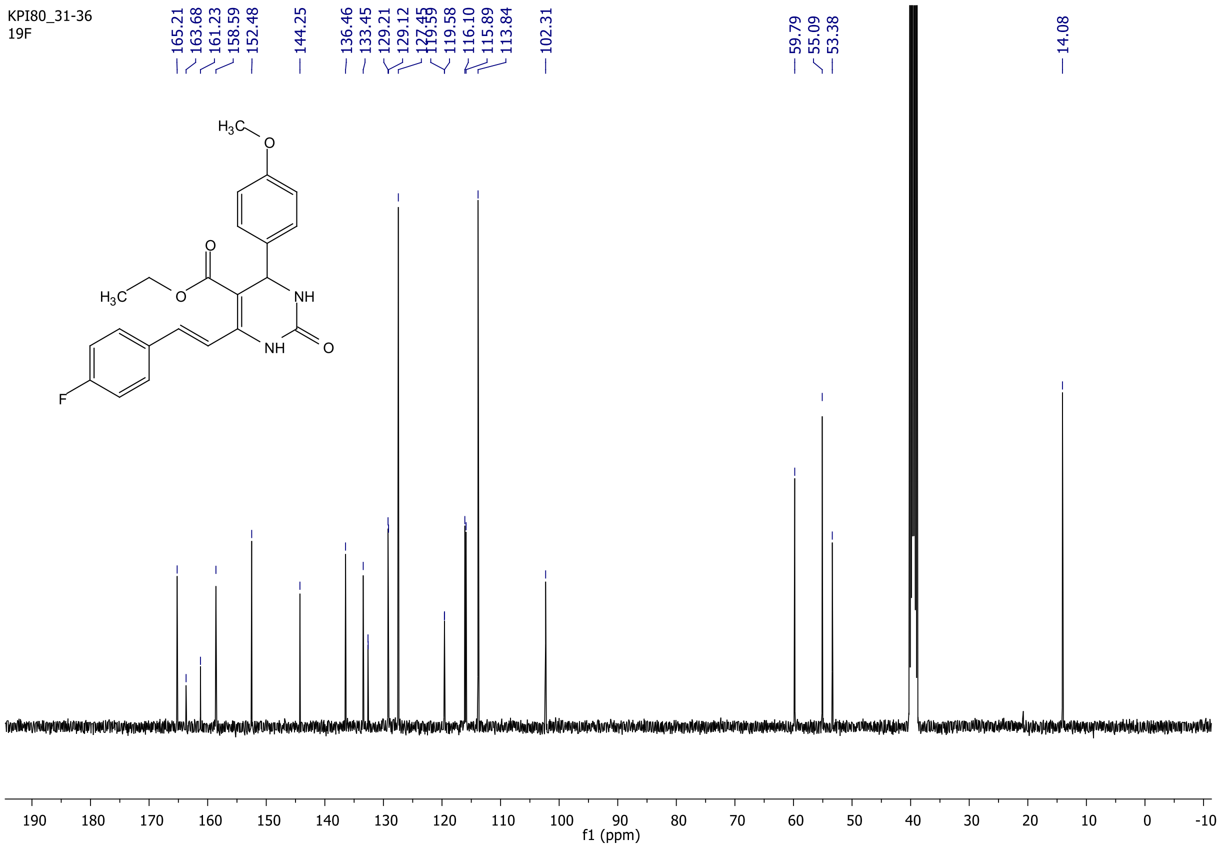

Figure 50 ^{13}C NMR of **21** in DMSO-d6

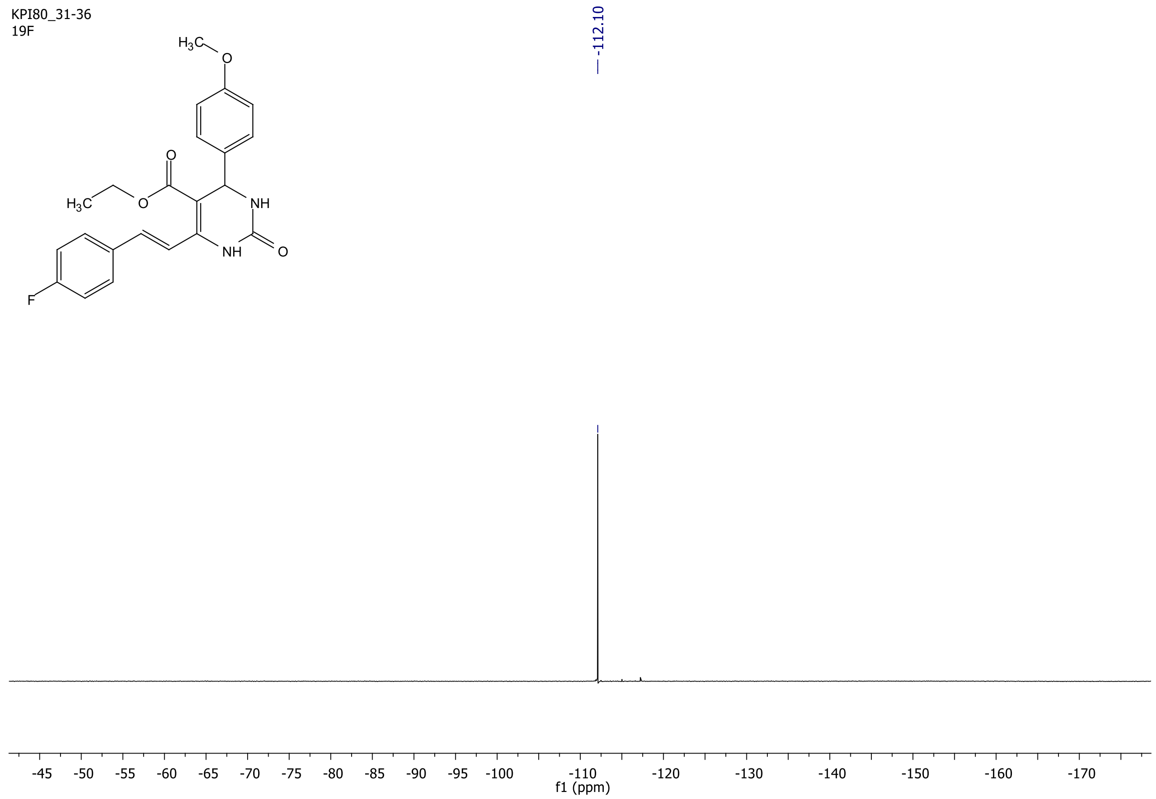

Figure 51 ^{19}F NMR of **21** in DMSO-d6

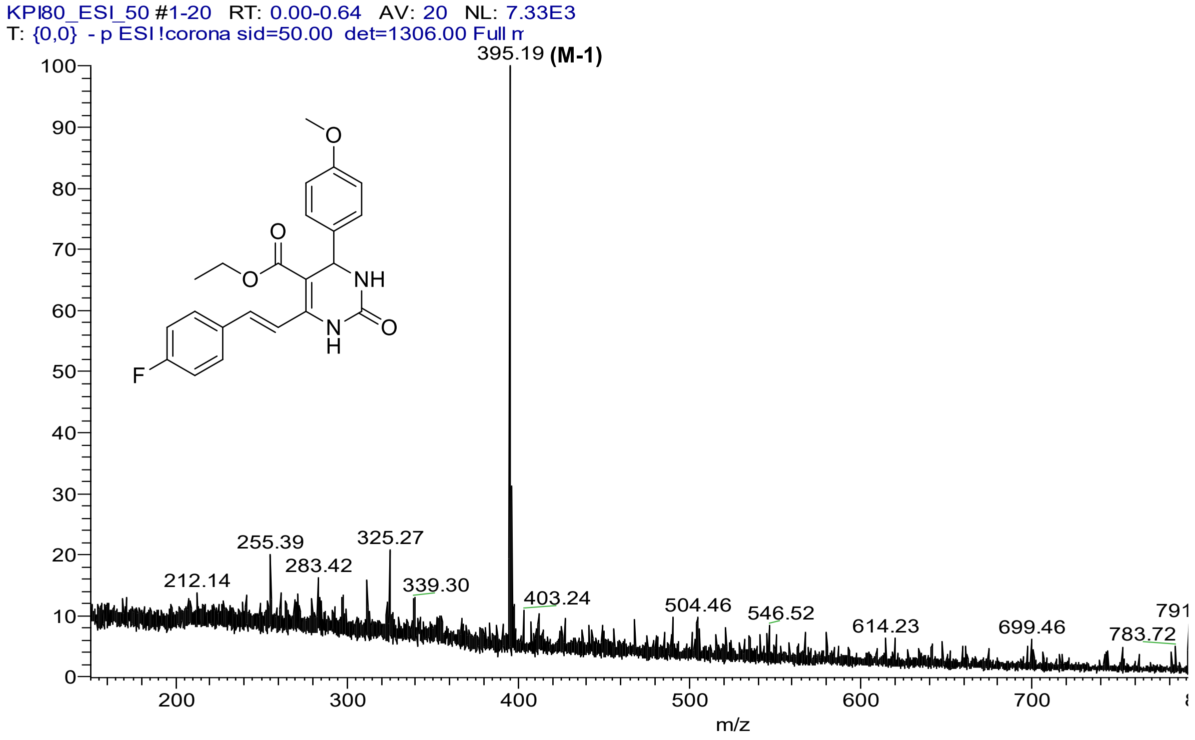

Figure 52 ESI-MS of **21**
